# Supplementary material for: Agalma: an automated phylogenomics workflow
Source: BMC Bioinformatics. 2013 Nov 19;14:330. doi: 10.1186/1471-2105-14-330 (PMC3840672; doi:10.1186/1471-2105-14-330)
Supplement: Additional file 1 — HTML report for assembly of the sample data sets. The HTML report for the assembly of the test data sets from raw reads. The tabular report (index.html) provides an overview across the five assemblies for the ingroup taxa, and includes links (in the Catalog ID column) to detailed reports for the assembly of each species. Fasta files for the annotated transcripts have been removed from the report to reduce file size. [file 1471-2105-14-330-S1.zip › tabular/index.html]

Agalma Diagnostics


| Run | catalog | | | | | *sanitize* | | | | | | | *insert\_size* | | *remove\_rrna* | | | *assemble* | | | | | | | *postassemble* | | | | profile\_aggregate | | | | |
| --- | --- | --- | --- | --- | --- | --- | --- | --- | --- | --- | --- | --- | --- | --- | --- | --- | --- | --- | --- | --- | --- | --- | --- | --- | --- | --- | --- | --- | --- | --- | --- | --- | --- |
| ID | Catalog ID | Species (ITIS ID) | Library ID | Note | Sample Prep | Read pairs examined | Read pairs kept | Percent kept | Illumina quality threshold | Adapter fails | Quality fails | Base composition fails | Mean insert size (bp) | Standard deviation (bp) | Read pairs examined | Read pairs kept | Percent kept | Read pairs examined | Read pairs kept | Percent kept | Illumina quality threshold | Adapter fails | Quality fails | Base composition fails | Read pairs | Pairs mapped | Pairs discordant | Unpaired reads mapped | Wall Time (s) | User Time (s) | System Time (s) | Memory (KB) | Virtual Memory (KB) |
| 8/14-15/42/48-49 | SRX288431 | *Physalia physalis* (None) | SRR871528 | None | Trizol | Illumina TruSeq RNA Sample Prep Kit RNA Purification Beads ; 2 rounds | Illumina TruSeq RNA Sample Prep Kit | 36,481,773 | 34,303,347 | 94.0% | 28 | 24,141 | 2,477,355 | 284,140 | 246.04 | 111.25 | 34,303,347 | 33,128,210 | 96.6% | 31,176,195 | 31,176,195 | 100.0% | 33 | 0 | 0 | 0 | 31,176,195 | 82.9% | 1.3% | 85.8% | 33209.10 | 276715.95 | 8789.03 | 42,452,600 | 44,070,276 |
| 24-26/28-30/44/47/50 | SRX288276 | *Abylopsis tetragona* (None) | SRR871525 | None | Invitrogen Dynabeads mRNA DIRECT kit ; 1 round | Illumina TruSeq RNA Sample Prep Kit | 21,575,176 | 18,806,969 | 87.2% | 28 | 59,900 | 3,172,739 | 471,153 | 291.86 | 44.06 | 18,806,969 | 13,690,073 | 72.8% | 13,690,073 | 11,673,891 | 85.3% | 33 | 0 | 2,669,619 | 0 | 11,673,891 | 62.9% | 2.7% | 71.3% | 38361.50 | 337888.72 | 10166.70 | 36,889,628 | 38,507,308 |
| 10/12-13/52/56/58 | SRX288432 | *Craseoa lathetica* (None) | SRR871529 | None | Invitrogen Dynabeads mRNA DIRECT kit ; 1 round | Illumina TruSeq RNA Sample Prep Kit | 38,233,199 | 31,086,355 | 81.3% | 28 | 91,856 | 7,681,925 | 2,396,996 | 262.23 | 49.78 | 31,086,355 | 24,690,259 | 79.4% | 24,690,259 | 20,235,317 | 82.0% | 33 | 0 | 6,089,251 | 0 | 20,235,317 | 52.8% | 3.0% | 63.4% | 49962.71 | 377348.87 | 10969.68 | 39,331,468 | 40,949,144 |
| 9/16-17/53/55/57 | SRX288430 | *Nanomia bijuga* (None) | SRR871527 | None | Trizol | Invitrogen Dynabeads mRNA Purification Kit ; 2 rounds | Illumina TruSeq RNA Sample Prep Kit | 52,533,443 | 39,953,762 | 76.1% | 28 | 267,995 | 14,085,652 | 2,087,117 | 242.82 | 78.05 | 39,953,762 | 35,622,853 | 89.2% | 31,396,792 | 31,396,792 | 100.0% | 33 | 0 | 0 | 0 | 31,396,792 | 72.9% | 2.0% | 79.4% | 45061.62 | 340238.28 | 9558.68 | 42,515,052 | 44,132,732 |
| 11/21-22/45/51/54 | SRX288285 | *Agalma elegans* (None) | SRR871526 | None | Trizol | Illumina TruSeq RNA Sample Prep Kit RNA Purification Beads ; 2 rounds | Illumina TruSeq RNA Sample Prep Kit | 53,998,182 | 49,584,637 | 91.8% | 28 | 55,487 | 4,403,926 | 1,231,920 | 271.60 | 98.44 | 49,584,637 | 49,389,381 | 99.6% | 46,234,620 | 46,234,620 | 100.0% | 33 | 0 | 0 | 0 | 46,234,620 | 74.8% | 1.7% | 79.6% | 58606.79 | 492787.23 | 14163.88 | 46,747,752 | 48,365,428 |
