# Supplementary material for: Agalma: an automated phylogenomics workflow
Source: BMC Bioinformatics. 2013 Nov 19;14:330. doi: 10.1186/1471-2105-14-330 (PMC3840672; doi:10.1186/1471-2105-14-330)
Supplement: Additional file 1 — HTML report for assembly of the sample data sets. The HTML report for the assembly of the test data sets from raw reads. The tabular report (index.html) provides an overview across the five assemblies for the ingroup taxa, and includes links (in the Catalog ID column) to detailed reports for the assembly of each species. Fasta files for the annotated transcripts have been removed from the report to reduce file size. [file 1471-2105-14-330-S1.zip › tabular/SRX288431/index.html]

Physalia physalis


# *Physalia physalis*

> |  |  |
> | --- | --- |
> | **id** | SRX288431 |
> | **ncbi\_id** | 168775 |
> | **library\_id** | SRR871528 |
> | **library\_type** | TRANSCRIPTOMIC |
> | **sequencer** | Illumina HiSeq 2000 |
> | **seq\_center** | Dunnlab |
> | **sample\_prep** | Trizol | Illumina TruSeq RNA Sample Prep Kit RNA Purification Beads ; 2 rounds | Illumina TruSeq RNA Sample Prep Kit |

## Table of Contents

|  |  |  |
| --- | --- | --- |
| **Run 8** *sanitize* 2013-06-11T12:34:28.015880 node423 | - sanitize | ``` Wall Time (s)      :      1661.84 [sum] User Time (s)      :       500.95 [sum] System Time (s)    :       937.45 [sum] Memory (KB)        :       36,516 [max] Virtual Memory (KB):      297,844 [max] ``` |
| **Run 14** *insert\_size* 2013-06-11T13:02:10.705689 node423 | - insert\_size | ``` Wall Time (s)      :        34.63 [sum] User Time (s)      :       102.70 [sum] System Time (s)    :         6.63 [sum] Memory (KB)        :       43,816 [max] Virtual Memory (KB):      309,000 [max] ``` |
| **Run 15** *remove\_rrna* 2013-06-11T13:02:46.215607 node423 | - remove\_rrna | ``` Wall Time (s)      :      1753.13 [sum] User Time (s)      :      8835.66 [sum] System Time (s)    :       898.99 [sum] Memory (KB)        :       63,492 [max] Virtual Memory (KB):      332,544 [max] ``` |
| **Run 42** *assemble* 2013-06-18T15:24:43.207732 node459 | - assemble | ``` Wall Time (s)      :     25126.54 [sum] User Time (s)      :    144001.33 [sum] System Time (s)    :      6652.64 [sum] Memory (KB)        :       46,464 [max] Virtual Memory (KB):      312,224 [max] ``` |
| **Run 48** *postassemble* 2013-06-19T03:46:29.142811 node424 | - postassemble | ``` Wall Time (s)      :      8513.66 [sum] User Time (s)      :    123722.37 [sum] System Time (s)    :       432.06 [sum] Memory (KB)        :      267,772 [max] Virtual Memory (KB):      537,688 [max] ``` |
| **Run 49** *load* 2013-06-19T06:08:24.641934 node424 |  | ``` Wall Time (s)      :        42.69 [sum] User Time (s)      :         3.66 [sum] System Time (s)    :         1.00 [sum] Memory (KB)        :      115,148 [max] Virtual Memory (KB):      381,608 [max] ``` |

## sanitize (Run 8)

> Filters raw paired-end Illumina data to remove very low quality read pairs,
> read pairs with adapter sequences, and read pairs with highly skewed base
> composition. It then randomizes the order of reads in the files (applying
> the same order of randomization to each file in the pair) to make it simple
> to get random subsets of read pairs in later analyses. Finally, fastqc is
> run to profile the quality of the reads.

#### Illumina Filtering

|  |  |
| --- | --- |
| **Read pairs examined** | 36,481,773 |
| **Read pairs kept** | 34,303,347 |
| **Percent kept** | 94.0% |
| **Illumina quality threshold** | 28 |
| **Adapter fails** | 24,141 |
| **Quality fails** | 2,477,355 |
| **Base composition fails** | 284,140 |


#### FastQC reports

|  |  |
| --- | --- |
| 8.fastqc.1  ``` PASS Basic Statistics  PASS Per base sequence quality  PASS Per sequence quality scores  FAIL Per base sequence content  FAIL Per base GC content  WARN Per sequence GC content  PASS Per base N content  PASS Sequence Length Distribution  PASS Sequence Duplication Levels  PASS Overrepresented sequences  WARN Kmer Content ``` | 8.fastqc.2  ``` PASS Basic Statistics  PASS Per base sequence quality  PASS Per sequence quality scores  FAIL Per base sequence content  FAIL Per base GC content  PASS Per sequence GC content  PASS Per base N content  PASS Sequence Length Distribution  PASS Sequence Duplication Levels  PASS Overrepresented sequences  WARN Kmer Content ``` |

> FastQC is a tool from Babraham Bioinformatics that generates detailed
> quality diagnostics of NGS sequence data.

#### Resourse Usage

| Wall Time (s) | User Time (s) | System Time (s) | Memory (KB) | Virtual Memory (KB) |
| --- | --- | --- | --- | --- |
 1373.05 [sum] | 437.86 [sum] | 818.20 [sum] | 14,931,256 [max] | 15,445,104 [max] |

 Show/hide details

| Command | Stage | Wall Time (s) | User Time (s) | System Time (s) | Memory (KB) | Virtual Memory (KB) |
| --- | --- | --- | --- | --- | --- | --- |
| randomize | randomize | 161.14 | 49.25 | 17.06 | 14,931,256 | 15,445,104 |
| fastqc | fastqc | 5.12 | 3.37 | 0.16 | 346,632 | 6,134,988 |
| filter\_illumina | sanitize | 1206.79 | 385.23 | 800.98 | 1,336 | 16,100 |

 Back to TOC

## insert\_size (Run 14)

> Estimates the insert size distribution of paired-end Illumina data by assembling a subset
> of the data and mapping read pairs to it. The insert size does not include the adapters
> added during library preparation.

|  |  |
| --- | --- |
| **Mean insert size (bp)** | 246.04 |
| **Standard deviation (bp)** | 111.25 |

> A histogram of insert sizes.

#### Resourse Usage

| Wall Time (s) | User Time (s) | System Time (s) | Memory (KB) | Virtual Memory (KB) |
| --- | --- | --- | --- | --- |
 33.34 [sum] | 102.29 [sum] | 6.34 [sum] | 476,604 [max] | 2,137,452 [max] |

 Show/hide details

| Command | Stage | Wall Time (s) | User Time (s) | System Time (s) | Memory (KB) | Virtual Memory (KB) |
| --- | --- | --- | --- | --- | --- | --- |
| filter\_illumina | subset | 3.83 | 1.14 | 2.36 | 1,296 | 16,100 |
| velveth | subset\_oases.oases\_assemblies.k31 | 1.26 | 5.88 | 0.27 | 476,604 | 2,137,452 |
| velvetg | subset\_oases.oases\_assemblies.k31 | 4.16 | 10.94 | 0.17 | 175,652 | 1,522,428 |
| oases | subset\_oases.oases\_assemblies.k31 | 0.71 | 0.63 | 0.05 | 54,084 | 71,504 |
| velveth | subset\_oases.oases\_assemblies.k41 | 1.30 | 6.77 | 0.28 | 440,248 | 1,547,628 |
| velvetg | subset\_oases.oases\_assemblies.k41 | 3.00 | 8.00 | 0.10 | 152,468 | 1,521,156 |
| oases | subset\_oases.oases\_assemblies.k41 | 0.59 | 0.52 | 0.05 | 49,528 | 66,992 |
| velveth | subset\_oases.oases\_assemblies.k51 | 1.29 | 6.36 | 0.27 | 432,608 | 2,137,452 |
| velvetg | subset\_oases.oases\_assemblies.k51 | 2.22 | 5.98 | 0.10 | 110,420 | 1,263,620 |
| oases | subset\_oases.oases\_assemblies.k51 | 0.50 | 0.43 | 0.04 | 46,764 | 64,252 |
| velveth | subset\_oases.oases\_assemblies.k61 | 1.11 | 4.44 | 0.25 | 392,352 | 1,547,628 |
| velvetg | subset\_oases.oases\_assemblies.k61 | 1.86 | 4.78 | 0.08 | 110,168 | 1,263,620 |
| oases | subset\_oases.oases\_assemblies.k61 | 0.45 | 0.38 | 0.04 | 45,708 | 63,192 |
| filter\_illumina | stats\_subset | 0.48 | 0.09 | 0.25 | 1,292 | 16,100 |
| bowtie2-build | bowtie | 4.88 | 4.76 | 0.07 | 104,228 | 178,564 |
| bowtie2 | bowtie | 3.91 | 39.51 | 1.89 | 382,172 | 1,212,624 |
| samtools | bowtie\_to\_bam.bam | 0.65 | 0.61 | 0.02 | 4,700 | 21,400 |
| samtools | bowtie\_to\_bam.sort | 1.00 | 0.94 | 0.04 | 147,068 | 165,616 |
| samtools | bowtie\_to\_bam.index | 0.08 | 0.07 | 0.00 | 2,076 | 19,680 |
| insert\_stats | estimate\_insert | 0.07 | 0.05 | 0.01 | 1,292 | 16,072 |

 Back to TOC

## remove\_rrna (Run 15)

> Assembles and identifies ribosomal RNA (rRNA) sequences, removes read pairs
> that map to these rRNA sequences, and provides a variety of diagnostics about
> rRNA. A single exemplar sequence is presented for each type of rRNA that is
> found, but rRNA read pairs are excluded by mapping to a large set of rRNA
> transcripts that are derived from multiple assemblies over a range of data
> subset sizes.

|  |  |
| --- | --- |
| **Read pairs examined** | 34,303,347 |
| **Read pairs kept** | 33,128,210 |
| **Percent kept** | 96.6% |

#### large-mito-rRNA / 1 target(s) / 4,260 pairs removed (0.01%)

**Locus\_1000000.924\_Transcript\_1/1\_Confidence\_1.000\_Length\_1680:**

|  |  |
| --- | --- |
| **Mean coverage** | 419.3 |
| **Median coverage** | 435 |
| **Min coverage** | 41 |
| **Max coverage** | 895 |

  
  

```
  >large-mito-rRNA|Locus_1000000.924_Transcript_1/1_Confidence_1.000_Length_1680|Run15|SRX288431
  ATTAGGGATCAATTCTCCTATAAAAAAGTCCCCTGTTAGTTTTTAAAATGTATAATACTAAATGAATACATATACTAAAC
  GGAGACGAATAGATGATACTGAACTGAAACATCTTAGTACTGCACAAAGATTGGCTAGATAGTAAAGGCGATTGAACTCA
  AGCCCAGACTTCTTGATCAAATAAGTCCTTGGATTTTATTTATAGCGTGAGTACTGTGAAGGAAATTAATACATTGAACC
  CATAACCCTTGTTTTACAATTACCTTTTGTATAATGGGTTTACAAGAATTAATATAGGCAAGTTTGTATACTATTTAATA
  AGTATAAGTCTATTTAACCCGAAACCGTGTGAAGTCTATTTAACCCGAAACCGTGTGATCTAATTGTGATCAACAGAAAG
  TGTTAATTGATGAGTGTAGAATAACTCTCAAATGAATTGCAATTAGCGGCGAAAAACTAATCGAACTCGGATATAGCTGG
  TTACCTGTGAAACTTATTTTAGTAATAAATCCTTTTAGGATATTCAGATTATTGATTACAACATCAGTACTCGAAAGGGT
  AACAACCTTAACCAAGAAAAAATAAGCTGGTTGAACAATTTGAATAAATGTAAACCATTGAAACCCAAAGAATTTAGGCT
  TAGAAACAGCCAGGTTATTATGATAACGTAAAAGTTCACTTTAATAAAGGGATACCTTATAAATATAGCTCTAGTTCATT
  AAACTTTTTGGATTAAAATAGTAACAGGTCATCCCAATTGTCAAATCTATTGGGTGTGATAATGTAAACATAATTAAAAC
  TTATTTATTTCTGAGATTTCTTATTTTAAGTTTTGACTTATTTTAAGTTTTGATTAAGGTTATACGGCACCTCACTAAAC
  CAGGGTGTGTTATTTCAGATTCTAGAAAATGAATAAAGACTTACCGTATTTAACTAAATCAAGAGGACGTAGATAAAAGG
  TTTAATTTTCTTTAAGGAACTCGGCAAACTAAGATGCCGTCTGTTTACCAAAAACATAGCTTTTATATAAACTCAAAGTG
  ATACCTGCCCAGTGGTTTGTAATATAAGGCATTAACTTTGCTTTTATTAAACTAAACGGACGCGGTAACCTGACCGTGGT
  AATGTAGCATAATCAGTCGCCATTTAATTGTTGGATAGTATGAATGGTTTAACGAGCATCTCACTGTCTTGAAGAGAAAC
  CCTGTGAAATTGGATTAGTAGTTAAGATACTACTTAAAATTGTAAGACGAAAAGACCCTATAGAGCTTAACTAAAGCATC
  TTTCTACTAAATGAATTAATAGATTCTGTTATGATAGGTGTTGTTAGTTTGGTTGGGGCGACCGCCTTCTATAATAAACG
  AAGGTATTCAAAGTTAATATTAGAATTTATTGTATAACCCATGAGTTTAACAATTACTAAAGTAGGTTATAATGACCCGT
  TGTGTGTGTGAAAAAACCCCAACGATAAATAGATAAAAGCTACCTTAGGGATAACAGGATAATTTAATCTTAGAGACCTT
  ATCGAAGCTTAAGTTTGTCACCTCTATGTTGAATTAAGATATCCCAATAGCGCAGAAGTTATTAAAGGTGGGTCTGTTCG
  ACCCTTAAAATCTTACATGATTTGAGTTCATTCCGTCGCGAGACAGGAAGGTTTCTATCTACAATTTGTTAAATTAAAAC
```

Top NCBI nt hit: gnl|BL\_ORD\_ID|267832 gi|283481139|emb|FN424145.1| Staurotheca pachyclada mitochondrial partial 16S rRNA gene, isolated from Antarctica, Ross Sea (E-value: 3.65376e-116)

#### large-nuclear-rRNA / 1 target(s) / 848,994 pairs removed (2.47%)

**Locus\_100000.34\_Transcript\_1/1\_Confidence\_1.000\_Length\_3839:**

|  |  |
| --- | --- |
| **Mean coverage** | 6885.5 |
| **Median coverage** | 6,957 |
| **Min coverage** | 150 |
| **Max coverage** | 14,694 |

  
  

```
  >large-nuclear-rRNA|Locus_100000.34_Transcript_1/1_Confidence_1.000_Length_3839|Run15|SRX288431
  ATCAGACAAGACTACCCGCTGAATTTAAGCATATTAATAAGCGGAGGAAAAGAAACTAACAAGGATTCCCCTAGTAACGG
  CGAGTGAAGCGGGAAGAGCTCAAACTTAAAATCTCCGTTGCTTGCAACGGCGAATTGTAGTCTCGAGAAGCGTTTTCAAG
  GCGAACGCGTAGTACTTAAATTGCTTGGAACGGCATATCGTAGAGGGTGACAATCCCGTACGTGGTACTGCGCGTCGCCG
  AATTATGCGCTTTCGATGAGTCGGGTTGCTTGGGAATGCAGCCCAAAACGGGAGGTAAACTCCTTCTAAAGCTAAATACC
  GGGAACAGCTCAAGCTTAAAATCTCTGTTGCTTGCAACGGCGAATTGTAGCCTCGAGAAGCGTTTTAAAACCGGATGCAT
  TGTGCTTAAGTTGCTAGGAACAGCACATCGTAGAGGGTGATAATCCCGTACGCGGTACAGTGCACCGGTCATGATGCGCT
  TTCTATGAGTCGGGTTGCTTGGGAATGCAGCCCAAAATGGGAGGTAAACTCCTTCTAAAGCTAAATATTGGCACGAGACC
  GATAGCGAACAAGTACCGTGAGGGAAAGATGAAAAGCACTTTGAAAAGAAAGTTAATAGTACGTGAAACCGTTAGAAGGG
  AAACGCATGGAATTAGCAATGCGCTGTCGAGATTCAGACGACGGGCGAGCGGCACCGGCGTTTAACGGATCCGAATGGAC
  CGTTTGCGTCGATCGCTGTTCTCTCGTTGTCGCATTTCCCGGCAGTGCGCGTCAACAGGTGTTGGAACCGGGTGATACGC
  CCTGCGAGAAGGTAGCTGACCTACGGGACAGTGTTATAGCTCGCGGTGTGCTGACTCGGATCCGACAGAGGTGTCTCAGC
  GCGTGCCCTTCTGGGCTGGCTTCCGTCTCTTCGCTCGTGCGTGAGCATGGTGGACTGCGTGCAGTGCACTTGAACTTCGT
  GCGTTCGTCGGGGGCGTGAATGCACACGACGCGCTCTGGTTGTTGGCGGTCATATGGTTTCATGCGACCCGTCTTGTAAC
  ACGGACCAAGGAGTCTAACGTGTGTGCGAGTCTTGGGGTGATAGAAACCCATCGGCGCAATGAAAGTAAAGGTTCCCTTG
  CGGAGCTGAGGTGAGATCCCTCTCGGCTAGCCGAGAGGGCGCATCATCGACCGACCTATTCTACTCTTAGAAAGGTTTGA
  GTAAGAGCACACCTGTTGGGACCCGAAAGATGGTGAACTATGCTTGAGTAGGGCGAAGCCAGAGGAAACTCTGGTGGAGG
  CTCGTAGCGATTCTGACGTGCAAATCGATCGTCAAACTTGAGTATAGGGGCGAAAGACTAATCGAACCATCTAGTAGCTG
  GTTCCCTCCGAAGTTTCCCTTAGGATAGCTGGAACTCGGAACAGTTTTATCAGGTAAAGCGAATGATTAGAGGTCTTAGG
  GTTGAAACAACCTTAACCTATTCTCAAACTTTAAATTGGTAAGAAGCCCGACTTGCTTGATTGAAGTAGGGCACAGAATG
  AGAGTTCTTAGTGGGCCATTTTTGGTAAGCAGAACTGGCGATGCGGGATGAACCGAACGCTGAGTTAAGGCGCCTAAATC
  GACGCTCATCAGACCCCACAAAAGGTGTTGGTTGATCCAGACAGCAGGACGGTGGCCATGGAAGTCGGAATCCGCTAAGG
  AGTGTGTAACAACACACCTGCCGAATCAACTAGCCCTGAAAATGGATGGCGCTCAAGCGTCGTGCCTATACTCAGCCGTC
  GGAGTAAATAGCCAAGCTCCGACGAGTAGGAGGGCGTGGGGGTCGTGACGCAGCCTTTGGCGCGAGCCTGGGTGAAACGG
  CCTCCAGTGAAGATCTTGGTGGTAGTAGCAAATATTCAAATGAGAACTTTGAAGACCGAAGTGGAGAAAGGTTCCATGTG
  AACAGCAGTTGGACATGGGTTAGTCGATCCTAAGAGATAGGGAAACTCCGTTTCAAAGTGTCCGATTCTCTCGGACCCTA
  GATCGAAAGGGAATCGGGTTAAAATTCCCGAACCAGAACGTGGATATTCGCGCGCCGTTCACGCGGTGTGTGACGTGCGG
  TAACGCAACTGAACTCGGAGACGTCGGCAGGAGCCCTGGGAAGAGTTCTCTTTTCTTGTTAACGGCCTGACACCATGGAA
  TCTGATTGCCAGGAGATATGGTCCGATGGCCGGTAAAGCACCACACTTCATGTGGTGTCCGGTGCGCTCCTGAAGGCCCT
  TGAAAATCCGAGGGAAAGATTGATTTTCGCATCTGTTCGTACTCATAACCGCAGCAGGTCTCCAAGGTGAGCAGCCTCTG
  GTCGATAGAACAATGTAGGTAAGGGAAGTCGGCAAAACAGATCCGTAACTTCGGGAAAAGGATTGGCTCTAAGGATTGGG
  TTTGTCGGGCTGAGACTTGAAGCAAGCGGTACCGACCTGGACTGGCTTCGGCTCACCTCCCTCTCACGAGGGCGGTAGGC
  TGTGGCCGGACTGGTGACGAACCGCTCGTGGATTGGCCCAGCTATGCTCGAAAGAGCAGTTCGGCAGACGATTAACAATC
  AACTTAGAACTGGTACGGACAAGGGGAATCCGACTGTTTAATTAAAACAAAGCATTGCGATGGCCGGAAACGGTGTTGAC
  GCAATGTGATTTCTGCCCAGTGCTCTGAATGTCAAAGTGAAGAAATTCAACCAAGCGCGGGTAAACGGCGGGAGTAACTA
  TGACTCTCTTAAGGTAGCCAAATGCCTCGTCATCTAATTAGTGACGCGCATGAATGGATTAACGAGATTCCCACTGTCCC
  TATCTACTATCTAGCGAAACCACAGCCAAGGGAACGGGCTTGGCAAAATCAGCGGGGAAAGAAGACCCTGTTGAGCTTGA
  CTCTAGTCTGACTTTGTGAAAAGACATAGGAGGTGTAGGATAGGTGGGAGCATTCGTGCGACGGTGAAATACCACTACTC
  TTATAGTTTTTTTACTTATTCGATTGAGCGGAAGCGAGCTTCACGGCTCATTTTCTAGAATTAAGGCCCCGTTGGCGGGT
  CGATCCGTGTCGAAGACACTGTCAGGTTGGGAGTTTGGCTGGGGCGGCACATCTGTCAAATGATAACGCAGGTGTCCTAA
  GGTGAGCTCAATGAGAACGGAAATCTCATGTAGAACAAAAGGGTAAAAGCTCACTTGATTTTGATTTTCAGTATGAATAC
  AAACTGTGAAAGCATGGCCTATCGATCCTTTAGTCTTTAGGAGTTTTAAGCTAGAGGTGTCAGAAAAGTTACCACAGGGA
  TAACTGGCTTGTGGCAGCCAAGCGTTCATAGCGACGTTGCTTTTTGATCCTTCGATGTCGGCTCTTCCTATCATTGTGAA
  GCAGAATTCACCAAGTGTTGGATTGTTCACCCACTAATAGGGAACGTGAGCTGGGTTTAGACCGTCGTGAGACAGGTTAG
  TTTTACCCTACTGATGAAGTGTTGTTGCAATAGTAATTCTGCTCAGTACGAGAGGAACCGCAGATTCAGACAATTGGCAT
  TTGCACTTGCTTGAAAAGGCAATGGTGCGAAGCTACCATCTGTTGGATTATGACTGAACGCCTCTAAGTCAGAATCCGTG
  CTAGAAAGCAATGATAATTACCTCTGGATAATCTTAGGCGAATAAGAATAGAGCGGCTTCGGTCGTTCCTGAATCTCAAT
  GCACTGAACGAGAGAAAAACTCGTGTTGTGCTGCAACTATCAAATTCTAAAATTTTCAGAGATAAATCCTATGCAGACGA
  CTTAAACAAGAACGTGGTATTGTAAAAAGCAGAGTAGCCTCTGTGCTACGATCTTCTGAGATTAAGCCTCTGTTCGTAG
```

Top NCBI nt hit: gnl|BL\_ORD\_ID|75271 gi|55859492|emb|AJ864475.1| Orpinomyces sp. OUS1 partial 18S rRNA gene, ITS1, 5.8S rRNA gene, ITS2 and partial 28S rRNA, clone V5-1 (E-value: 0.0)

#### small-mito-rRNA / 1 target(s) / 952 pairs removed (0.00%)

**Locus\_1000000.348\_Transcript\_1/1\_Confidence\_1.000\_Length\_488:**

|  |  |
| --- | --- |
| **Mean coverage** | 302.6 |
| **Median coverage** | 335 |
| **Min coverage** | 9 |
| **Max coverage** | 619 |

  
  

```
  >small-mito-rRNA|Locus_1000000.348_Transcript_1/1_Confidence_1.000_Length_488|Run15|SRX288431
  CTTAACTCTTTTGATTTTGGAGAAGTGAAAACACAGGGATTATTTATACATGTTAGTGGAAGCGAACAAGTGAGTAAAAT
  TAGTAACTATCTAATTGGTATCAGGGTGAGTACAATAACAAGTGCTTTAACCTAAGACACCTAGCTCTTATAGCCACGTT
  CTAGATGTAATAATATGGAATTTCAGACAGCAGTAAAGAGTCCTATACAATTAACTAAAGTTAGATATGGTTACCACCTG
  TAGTTTTGTCTAATTAAGTGCCAGCAGACGCGGTTAAACTTAAGAAGCTAGTTTTCACGTAAAATTAGGTTATTAGATGC
  GTAGGGATTCATAAAAGTATTTTGAAAGTTTTTGATTTGGTTAAATAAACAAAGACCTTAAAACCAAGGTTTAATTAATA
  ATACAATGAACTCTTAAGCATGAATTAAGAAACCTCAAATAGGATTAGATACCCTAGTAGATTCTTATGTAAACGGATAT
  AGTCAACC
```

Top NCBI nt hit: Unknown (E-value: 1.0)

#### small-nuclear-rRNA / 1 target(s) / 320,931 pairs removed (0.94%)

**Locus\_500000.16\_Transcript\_2/4\_Confidence\_0.625\_Length\_1909:**

|  |  |
| --- | --- |
| **Mean coverage** | 4781.7 |
| **Median coverage** | 5,034 |
| **Min coverage** | 3 |
| **Max coverage** | 12,783 |

  
  

```
  >small-nuclear-rRNA|Locus_500000.16_Transcript_2/4_Confidence_0.625_Length_1909|Run15|SRX288431
  TTTGTACGTAGTTACCTGGTTGATCCTGCCAGTAGTCATATGCTTGTCTCAAAGATTAAGCCATGCATGTCTAAGTATAA
  GCACTTGTACTGTGAAACTGCGAATGGCTCATTAAATCAGTTATCGTTTATTTGATTGTACTTTTACTACATGGATACCT
  GTGGTAATTCTAGAGCTAATACATGCGAAAAATCCCGACTTCTGGAAGGGATGTATTTATTAGATTAAAAACCAATGCGG
  GTTCTCTTTTGAGCTCGTTTTCTTGGTGATTCATGATAACTTTTCGAATCGCATGGCCTTTGCGCCGGCGATGTTTCATT
  CAAATTTCTGCCCTATCAACTGTCGATGGTAAGGTAGTGGCTTACCATGGTTGTAACGGGTGACGGAGAATTAGGGTTCG
  ATTCCGGAGAGGGAGCCTGAGAAACGGCTACCACATCTAAGGAAGGCAGCAGGCACGAAAATTACCCAATCCCGACTCGG
  GGAGGTAGTGACAAGAAATAACGATACGGGGTCTTAATAGGTCTCGCAATTGGAATGAGTACAATTTAAATCCTTTAACG
  AGGATCAATTGGAGGGCAAGTCTGGTGCCAGCAGCCGCGGTAATTCCAGCTCCAATAGCGTATATTAAAGTTGTTGCAGT
  TAAAAAGCTCGTAGTTGGATTTCGGAGTGGGCCAGTCGGTCCGCCGCGAGGTGTGTACTGATTGGTCTGCTCTTCTTCGC
  AAAGACTCCGCGTGCGCTTCGCTGTGTGTGCGTAGGATTTGCGACGTTTACTTTGAAAAAATTAGAGTGTTCAAAGCAGG
  CTATCGCTTGAATACATGAGCATGGAATAATGGAATAGGACTTTGGTCCCATTTTGTTGGTTTCTAGGACCGAAGTAATG
  ATTAAGAGGGACAATTGGGGGCATCCGTATTTCGTTGTCAGAGGTGAAATTCTTGGATTTACGAAAGACGAACAACTGCG
  AAAGCATTTGCCAAGAGTGTTTTCATTAATCAAGAACGAAAGTTAGAGGATCGAAGACGATCAGATACCGTCCTAGTTCT
  AACCATAAACGATGTCGACTAGGGATCAGCGGGCGTTATTGTACGACCCCGTTGGCACCTTACGGGAAACCAAAGTTTTT
  GGATTCCGGGGGAAGTATGGTTGCAAAATTGAAACTTAAAGGAATTGACGGAAGGGCACCACCAGGAGTGGAGCCTGTGG
  CTCAATTTGACTCAACACGGGAAAACTTACCAGGTCCAGACATAGTAAGGATTGACAGGTTGAGAGCCCTTTCTTGATTC
  TATGGGTGGTGGTGCATGGCCGTTCTTAGTTGGTGGAGTGATTTGTCTGGTTAATTCCGTTAACGAACGAGACCTTAACC
  AGCTAAATAGTCACACGATTCTCGAATCGTGACTGACTTCTTAGAGGGACTGTTGGTGTTTAACCAAAGTCAGGAAGGCA
  ATAACAGGTCTGTGATGCCCTTAGATGTTCTGGGCCGCACGCGCGCTACACTGTCGGATTCAACGAGTCTTAACCTTAAC
  CGAAAGGTTTGGGTAATCTTTTGAAAGTCCGACGTGATGGGGATTGATCATTGCAATTATTGATCATGAACGAGGAATTC
  CTAGTAAGCGCGAGTCATCAGCTCGCGTTGATTACGTCCCTGCCCTTTGTACACACCGCCCGTCGCTACTACCGATTGAA
  TGATTTAGTGAGATCTTCGGATTGGTATCGTCGCGTCTTCACGGATGCGACGAGGCTGAAAAGTTGCTCAAACTTGATCA
  TTTAGAGGAAGTAAAAGTCGTAACAAGGTTTCCGTAGGTGAACCTGCGGAAGGATCATTACCGTCTACTTGTGTTCTCGT
  CAACTCGTTTTGACGCACACGCCACTGTGAACTGTATTAAGCAAGCGGGGTAACGCAAGCATCGTAGTA
```

Top NCBI nt hit: gnl|BL\_ORD\_ID|17796 gi|5931791|emb|AJ133551.1| Virgularia juncea 18S rRNA gene (E-value: 0.0)

#### Resourse Usage

| Wall Time (s) | User Time (s) | System Time (s) | Memory (KB) | Virtual Memory (KB) |
| --- | --- | --- | --- | --- |
 1745.68 [sum] | 8829.78 [sum] | 898.33 [sum] | 1,199,760 [max] | 2,116,492 [max] |

 Show/hide details

| Command | Stage | Wall Time (s) | User Time (s) | System Time (s) | Memory (KB) | Virtual Memory (KB) |
| --- | --- | --- | --- | --- | --- | --- |
| filter\_illumina | subset\_assemblies.500 | 0.03 | 0.01 | 0.01 | 1,292 | 16,100 |
| velveth | subset\_assemblies.500.oases\_assemblies.k61 | 0.06 | 0.42 | 0.06 | 201,300 | 2,096,752 |
| velvetg | subset\_assemblies.500.oases\_assemblies.k61 | 0.02 | 0.05 | 0.01 | 1,360 | 194,204 |
| oases | subset\_assemblies.500.oases\_assemblies.k61 | 0.01 | 0.00 | 0.00 | 1,240 | 20,612 |
| filter\_illumina | subset\_assemblies.1000 | 0.05 | 0.01 | 0.03 | 1,292 | 16,100 |
| velveth | subset\_assemblies.1000.oases\_assemblies.k61 | 0.06 | 0.42 | 0.07 | 203,508 | 2,097,020 |
| velvetg | subset\_assemblies.1000.oases\_assemblies.k61 | 0.05 | 0.16 | 0.03 | 67,168 | 871,604 |
| oases | subset\_assemblies.1000.oases\_assemblies.k61 | 0.01 | 0.00 | 0.00 | 1,548 | 19,868 |
| filter\_illumina | subset\_assemblies.2500 | 0.10 | 0.03 | 0.05 | 1,292 | 16,100 |
| velveth | subset\_assemblies.2500.oases\_assemblies.k61 | 0.08 | 0.48 | 0.07 | 208,680 | 2,097,564 |
| velvetg | subset\_assemblies.2500.oases\_assemblies.k61 | 0.08 | 0.21 | 0.03 | 67,896 | 1,680,364 |
| oases | subset\_assemblies.2500.oases\_assemblies.k61 | 0.03 | 0.01 | 0.01 | 2,516 | 36,884 |
| filter\_illumina | subset\_assemblies.5000 | 0.41 | 0.05 | 0.12 | 1,292 | 16,100 |
| velveth | subset\_assemblies.5000.oases\_assemblies.k61 | 0.11 | 0.60 | 0.08 | 216,040 | 1,967,648 |
| velvetg | subset\_assemblies.5000.oases\_assemblies.k61 | 0.12 | 0.35 | 0.03 | 68,936 | 1,681,512 |
| oases | subset\_assemblies.5000.oases\_assemblies.k61 | 0.04 | 0.02 | 0.01 | 3,672 | 39,496 |
| filter\_illumina | subset\_assemblies.10000 | 0.54 | 0.11 | 0.23 | 1,292 | 16,100 |
| velveth | subset\_assemblies.10000.oases\_assemblies.k61 | 0.15 | 0.54 | 0.10 | 229,404 | 2,100,648 |
| velvetg | subset\_assemblies.10000.oases\_assemblies.k61 | 0.21 | 0.58 | 0.03 | 71,076 | 1,683,644 |
| oases | subset\_assemblies.10000.oases\_assemblies.k61 | 0.05 | 0.03 | 0.01 | 5,524 | 39,888 |
| filter\_illumina | subset\_assemblies.25000 | 0.98 | 0.25 | 0.59 | 1,292 | 16,100 |
| velveth | subset\_assemblies.25000.oases\_assemblies.k61 | 0.34 | 1.17 | 0.12 | 264,548 | 2,106,748 |
| velvetg | subset\_assemblies.25000.oases\_assemblies.k61 | 0.47 | 1.21 | 0.04 | 77,536 | 1,296,828 |
| oases | subset\_assemblies.25000.oases\_assemblies.k61 | 0.12 | 0.09 | 0.02 | 11,964 | 42,312 |
| filter\_illumina | subset\_assemblies.50000 | 1.91 | 0.53 | 1.15 | 1,292 | 16,100 |
| velveth | subset\_assemblies.50000.oases\_assemblies.k61 | 0.72 | 2.94 | 0.15 | 315,012 | 2,116,492 |
| velvetg | subset\_assemblies.50000.oases\_assemblies.k61 | 0.92 | 2.29 | 0.07 | 88,312 | 1,510,588 |
| oases | subset\_assemblies.50000.oases\_assemblies.k61 | 0.38 | 0.20 | 0.02 | 23,124 | 44,756 |
| filter\_illumina | subset\_assemblies.100000 | 4.00 | 1.08 | 2.27 | 1,296 | 16,100 |
| velveth | subset\_assemblies.100000.oases\_assemblies.k61 | 1.11 | 4.33 | 0.26 | 411,164 | 1,613,164 |
| velvetg | subset\_assemblies.100000.oases\_assemblies.k61 | 1.85 | 4.72 | 0.10 | 112,196 | 1,263,620 |
| oases | subset\_assemblies.100000.oases\_assemblies.k61 | 0.45 | 0.39 | 0.04 | 45,688 | 63,208 |
| filter\_illumina | subset\_assemblies.250000 | 10.31 | 2.72 | 5.74 | 1,292 | 16,100 |
| velveth | subset\_assemblies.250000.oases\_assemblies.k61 | 2.74 | 11.40 | 0.41 | 639,656 | 1,806,852 |
| velvetg | subset\_assemblies.250000.oases\_assemblies.k61 | 4.82 | 13.12 | 0.12 | 175,660 | 1,328,608 |
| oases | subset\_assemblies.250000.oases\_assemblies.k61 | 1.13 | 1.02 | 0.08 | 114,116 | 131,636 |
| filter\_illumina | subset\_assemblies.500000 | 18.23 | 5.51 | 11.68 | 1,292 | 16,100 |
| velveth | subset\_assemblies.500000.oases\_assemblies.k61 | 5.32 | 21.07 | 0.81 | 971,484 | 1,911,348 |
| velvetg | subset\_assemblies.500000.oases\_assemblies.k61 | 10.21 | 30.25 | 0.30 | 276,060 | 1,377,708 |
| oases | subset\_assemblies.500000.oases\_assemblies.k61 | 2.41 | 2.19 | 0.18 | 228,728 | 246,248 |
| filter\_illumina | subset\_assemblies.1000000 | 36.07 | 11.01 | 23.54 | 1,292 | 16,100 |
| velveth | subset\_assemblies.1000000.oases\_assemblies.k61 | 10.91 | 41.81 | 1.42 | 1,199,760 | 1,639,820 |
| velvetg | subset\_assemblies.1000000.oases\_assemblies.k61 | 21.43 | 67.52 | 0.52 | 439,192 | 1,833,784 |
| oases | subset\_assemblies.1000000.oases\_assemblies.k61 | 5.01 | 4.58 | 0.34 | 459,976 | 477,388 |
| makeblastdb | blast\_rrna | 0.03 | 0.01 | 0.01 | 8,172 | 44,988 |
| blastn | blast\_rrna | 7.95 | 7.53 | 0.39 | 199,484 | 2,004,124 |
| blastn | blast\_nt | 1.39 | 1.48 | 0.15 | 127,576 | 2,092,444 |
| bowtie2-build | bowtie | 0.53 | 0.31 | 0.05 | 98,660 | 175,964 |
| bowtie2 | bowtie | 529.13 | 8344.78 | 63.02 | 324,912 | 1,180,628 |
| samtools | bowtie\_to\_bam.bam | 39.18 | 37.76 | 0.81 | 3,404 | 20,112 |
| samtools | bowtie\_to\_bam.sort | 40.73 | 39.51 | 0.58 | 721,448 | 747,140 |
| samtools | bowtie\_to\_bam.index | 1.88 | 1.81 | 0.05 | 936 | 18,744 |
| samtools | bam\_pileup | 19.48 | 18.42 | 0.29 | 11,752 | 28,396 |
| samtools | bam\_extract\_ids.large-nuclear-rRNA | 6.03 | 3.64 | 0.37 | 2,084 | 18,748 |
| samtools | bam\_extract\_ids.small-mito-rRNA | 0.05 | 0.01 | 0.00 | 1,740 | 18,744 |
| samtools | bam\_extract\_ids.small-nuclear-rRNA | 2.28 | 1.32 | 0.07 | 2,072 | 18,748 |
| samtools | bam\_extract\_ids.large-mito-rRNA | 0.08 | 0.02 | 0.00 | 2,072 | 18,744 |
| exclude | exclude\_ids | 952.87 | 137.67 | 781.56 | 594,812 | 609,576 |

 Back to TOC

## assemble (Run 42)

> Assembles reads into transcripts, processes the assembly, and generates
> assembly diagnostics. Read pairs are first filtered at a more stringent
> mean quality threshold. Assemblies are then performed over a range of
> data subset sizes, which provides an indication of how sequencing effort
> impacts assembly results.

#### Illumina Filtering

|  |  |
| --- | --- |
| **Read pairs examined** | 31,176,195 |
| **Read pairs kept** | 31,176,195 |
| **Percent kept** | 100.0% |
| **Illumina quality threshold** | 33 |
| **Adapter fails** | 0 |
| **Quality fails** | 0 |
| **Base composition fails** | 0 |

#### Resourse Usage

| Wall Time (s) | User Time (s) | System Time (s) | Memory (KB) | Virtual Memory (KB) |
| --- | --- | --- | --- | --- |
 21906.50 [sum] | 143982.63 [sum] | 6635.09 [sum] | 42,452,600 [max] | 44,070,276 [max] |

 Show/hide details

| Command | Stage | Wall Time (s) | User Time (s) | System Time (s) | Memory (KB) | Virtual Memory (KB) |
| --- | --- | --- | --- | --- | --- | --- |
| filter\_illumina | quality\_filter | 849.92 | 117.78 | 724.54 | 1,336 | 16,100 |
| trinity | trinity.31176195 | 16034.99 | 80436.33 | 2808.84 | 42,452,600 | 44,070,276 |
| parallel | trinity.31176195 | 5021.59 | 63428.53 | 3101.71 | 1,364,120 | 5,237,244 |

 Back to TOC

## postassemble (Run 48)

> Cleans transcripts to remove any rRNA or vector sequences, then selects a
> single exemplar transcript for each gene. Vector sequences could include
> untrimmed adapters or plasmids (we sometimes find sequences in our data for the
> protein expression vectors used to manufacture the sample preparation enzymes).
> Raw reads are mapped back to the exemplars to estimate coverage and assign RPKM
> values. Finally, transcripts are annotated with blastx hits against SwissProt.

#### Assemblies

| Method | Read pairs | Genes | Mean Length (bp) | N50 Length (bp) | Links to assembly files |
| --- | --- | --- | --- | --- | --- |
| trinity | 31,176,195 | 42692 | 741.065843718 | 903 | [annotated transcripts], [rrna], [vectors] |

##### trinity

|  |  |
| --- | --- |
| **Read pairs** | 31,176,195 |
| **Pairs mapped** | 82.9% |
| **Pairs discordant** | 1.3% |
| **Unpaired reads mapped** | 85.8% |

Number of exemplar transcripts in full assembly with blastx hits: 15,351


#### Resourse Usage

| Wall Time (s) | User Time (s) | System Time (s) | Memory (KB) | Virtual Memory (KB) |
| --- | --- | --- | --- | --- |
 8150.54 [sum] | 123363.39 [sum] | 431.07 [sum] | 370,404 [max] | 1,214,408 [max] |

 Show/hide details

| Command | Stage | Wall Time (s) | User Time (s) | System Time (s) | Memory (KB) | Virtual Memory (KB) |
| --- | --- | --- | --- | --- | --- | --- |
| makeblastdb | clean\_rrna.assembly\_31176195\_trinity | 0.02 | 0.01 | 0.01 | 8,164 | 45,008 |
| blastn | clean\_rrna.assembly\_31176195\_trinity | 8.83 | 50.09 | 14.40 | 140,692 | 198,816 |
| blastn | clean\_univec.assembly\_31176195\_trinity | 9.47 | 51.66 | 19.95 | 140,484 | 200,212 |
| dustmasker | dustmasker.assembly\_31176195\_trinity | 11.35 | 10.59 | 0.60 | 8,260 | 43,572 |
| bowtie2-build | coverage.assembly\_31176195\_trinity | 23.15 | 22.52 | 0.37 | 185,704 | 236,728 |
| bowtie2 | coverage.assembly\_31176195\_trinity | 1195.57 | 18715.42 | 160.99 | 370,404 | 1,214,408 |
| coverage | coverage.assembly\_31176195\_trinity | 180.26 | 72.39 | 10.62 | 7,044 | 22,804 |
| blastx | nr\_annotate.assembly\_31176195\_trinity | 6721.88 | 104440.71 | 224.12 | 213,096 | 360,968 |

 Back to TOC
