# Supplementary material for: Agalma: an automated phylogenomics workflow
Source: BMC Bioinformatics. 2013 Nov 19;14:330. doi: 10.1186/1471-2105-14-330 (PMC3840672; doi:10.1186/1471-2105-14-330)
Supplement: Additional file 1 — HTML report for assembly of the sample data sets. The HTML report for the assembly of the test data sets from raw reads. The tabular report (index.html) provides an overview across the five assemblies for the ingroup taxa, and includes links (in the Catalog ID column) to detailed reports for the assembly of each species. Fasta files for the annotated transcripts have been removed from the report to reduce file size. [file 1471-2105-14-330-S1.zip › tabular/SRX288276/index.html]

Abylopsis tetragona


# *Abylopsis tetragona*

> |  |  |
> | --- | --- |
> | **id** | SRX288276 |
> | **ncbi\_id** | 316209 |
> | **library\_id** | SRR871525 |
> | **library\_type** | TRANSCRIPTOMIC |
> | **sequencer** | Illumina HiSeq 2000 |
> | **seq\_center** | Dunnlab |
> | **sample\_prep** | Invitrogen Dynabeads mRNA DIRECT kit ; 1 round | Illumina TruSeq RNA Sample Prep Kit |

## Table of Contents

|  |  |  |
| --- | --- | --- |
| **Run 24** *sanitize* 2013-06-11T15:52:56.291387 smp010 | - sanitize | ``` Wall Time (s)      :      1465.23 [sum] User Time (s)      :       408.99 [sum] System Time (s)    :       935.30 [sum] Memory (KB)        :       36,232 [max] Virtual Memory (KB):      298,132 [max] ``` |
| **Run 25** *insert\_size* 2013-06-11T16:17:25.526290 smp010 | - insert\_size | ``` Wall Time (s)      :        58.31 [sum] User Time (s)      :       189.29 [sum] System Time (s)    :        10.48 [sum] Memory (KB)        :       43,560 [max] Virtual Memory (KB):      309,040 [max] ``` |
| **Run 26** *remove\_rrna* 2013-06-11T16:18:29.588756 smp010 | - remove\_rrna | ``` Wall Time (s)      :      3917.14 [sum] User Time (s)      :     25999.10 [sum] System Time (s)    :       785.96 [sum] Memory (KB)        :       61,924 [max] Virtual Memory (KB):      331,468 [max] ``` |
| **Run 28** *sanitize* 2013-06-11T22:14:01.704745 smp011 | - sanitize | ``` Wall Time (s)      :      1512.86 [sum] User Time (s)      :       410.07 [sum] System Time (s)    :       922.61 [sum] Memory (KB)        :       36,220 [max] Virtual Memory (KB):      297,872 [max] ``` |
| **Run 29** *insert\_size* 2013-06-11T22:39:23.888288 smp011 | - insert\_size | ``` Wall Time (s)      :        77.06 [sum] User Time (s)      :       186.17 [sum] System Time (s)    :        12.30 [sum] Memory (KB)        :       43,556 [max] Virtual Memory (KB):      308,976 [max] ``` |
| **Run 30** *remove\_rrna* 2013-06-11T22:40:45.362539 smp011 | - remove\_rrna | ``` Wall Time (s)      :      3457.29 [sum] User Time (s)      :     21616.62 [sum] System Time (s)    :       794.23 [sum] Memory (KB)        :       60,860 [max] Virtual Memory (KB):      330,920 [max] ``` |
| **Run 44** *assemble* 2013-06-18T21:08:39.890297 node428 | - assemble | ``` Wall Time (s)      :     21358.54 [sum] User Time (s)      :    121913.52 [sum] System Time (s)    :      6582.68 [sum] Memory (KB)        :       46,480 [max] Virtual Memory (KB):      312,300 [max] ``` |
| **Run 47** *postassemble* 2013-06-19T03:04:46.351230 node428 | - postassemble | ``` Wall Time (s)      :     11410.13 [sum] User Time (s)      :    167800.79 [sum] System Time (s)    :       413.74 [sum] Memory (KB)        :      346,948 [max] Virtual Memory (KB):      619,812 [max] ``` |
| **Run 50** *load* 2013-06-19T06:14:59.095241 node428 |  | ``` Wall Time (s)      :        45.31 [sum] User Time (s)      :         4.99 [sum] System Time (s)    :         1.41 [sum] Memory (KB)        :      137,140 [max] Virtual Memory (KB):      403,480 [max] ``` |

## sanitize (Run 24)

> Filters raw paired-end Illumina data to remove very low quality read pairs,
> read pairs with adapter sequences, and read pairs with highly skewed base
> composition. It then randomizes the order of reads in the files (applying
> the same order of randomization to each file in the pair) to make it simple
> to get random subsets of read pairs in later analyses. Finally, fastqc is
> run to profile the quality of the reads.

#### Illumina Filtering

|  |  |
| --- | --- |
| **Read pairs examined** | 21,575,176 |
| **Read pairs kept** | 18,806,969 |
| **Percent kept** | 87.2% |
| **Illumina quality threshold** | 28 |
| **Adapter fails** | 59,900 |
| **Quality fails** | 3,172,739 |
| **Base composition fails** | 471,153 |


#### FastQC reports

|  |  |
| --- | --- |
| 24.fastqc.1  ``` PASS Basic Statistics  PASS Per base sequence quality  PASS Per sequence quality scores  FAIL Per base sequence content  FAIL Per base GC content  PASS Per sequence GC content  PASS Per base N content  PASS Sequence Length Distribution  WARN Sequence Duplication Levels  WARN Overrepresented sequences  WARN Kmer Content ``` | 24.fastqc.2  ``` PASS Basic Statistics  PASS Per base sequence quality  PASS Per sequence quality scores  FAIL Per base sequence content  FAIL Per base GC content  PASS Per sequence GC content  PASS Per base N content  PASS Sequence Length Distribution  WARN Sequence Duplication Levels  WARN Overrepresented sequences  WARN Kmer Content ``` |

> FastQC is a tool from Babraham Bioinformatics that generates detailed
> quality diagnostics of NGS sequence data.

#### Resourse Usage

| Wall Time (s) | User Time (s) | System Time (s) | Memory (KB) | Virtual Memory (KB) |
| --- | --- | --- | --- | --- |
 1212.09 [sum] | 344.96 [sum] | 799.13 [sum] | 8,782,036 [max] | 9,019,704 [max] |

 Show/hide details

| Command | Stage | Wall Time (s) | User Time (s) | System Time (s) | Memory (KB) | Virtual Memory (KB) |
| --- | --- | --- | --- | --- | --- | --- |
| randomize | randomize | 80.01 | 35.89 | 16.54 | 8,782,036 | 9,019,704 |
| fastqc | fastqc | 8.54 | 8.02 | 0.35 | 596,724 | 8,162,536 |
| filter\_illumina | sanitize | 1123.54 | 301.05 | 782.25 | 1,324 | 16,104 |

 Back to TOC

## insert\_size (Run 25)

> Estimates the insert size distribution of paired-end Illumina data by assembling a subset
> of the data and mapping read pairs to it. The insert size does not include the adapters
> added during library preparation.

|  |  |
| --- | --- |
| **Mean insert size (bp)** | 314.94 |
| **Standard deviation (bp)** | 136.04 |

> A histogram of insert sizes.

#### Resourse Usage

| Wall Time (s) | User Time (s) | System Time (s) | Memory (KB) | Virtual Memory (KB) |
| --- | --- | --- | --- | --- |
 57.11 [sum] | 188.51 [sum] | 10.02 [sum] | 564,016 [max] | 2,137,456 [max] |

 Show/hide details

| Command | Stage | Wall Time (s) | User Time (s) | System Time (s) | Memory (KB) | Virtual Memory (KB) |
| --- | --- | --- | --- | --- | --- | --- |
| filter\_illumina | subset | 6.38 | 1.66 | 4.12 | 1,284 | 16,104 |
| velveth | subset\_oases.oases\_assemblies.k31 | 2.64 | 11.11 | 0.64 | 564,016 | 2,137,456 |
| velvetg | subset\_oases.oases\_assemblies.k31 | 9.21 | 26.36 | 0.33 | 175,092 | 1,650,140 |
| oases | subset\_oases.oases\_assemblies.k31 | 2.04 | 1.41 | 0.09 | 61,744 | 79,168 |
| velveth | subset\_oases.oases\_assemblies.k41 | 2.76 | 10.38 | 0.56 | 531,252 | 2,137,456 |
| velvetg | subset\_oases.oases\_assemblies.k41 | 6.38 | 20.75 | 0.22 | 137,988 | 1,290,328 |
| oases | subset\_oases.oases\_assemblies.k41 | 1.51 | 1.01 | 0.08 | 52,772 | 70,276 |
| velveth | subset\_oases.oases\_assemblies.k51 | 2.31 | 9.23 | 0.54 | 493,148 | 1,678,704 |
| velvetg | subset\_oases.oases\_assemblies.k51 | 4.09 | 13.15 | 0.15 | 112,520 | 1,517,120 |
| oases | subset\_oases.oases\_assemblies.k51 | 0.88 | 0.77 | 0.08 | 47,624 | 65,108 |
| velveth | subset\_oases.oases\_assemblies.k61 | 2.20 | 7.91 | 0.47 | 450,976 | 1,744,240 |
| velvetg | subset\_oases.oases\_assemblies.k61 | 3.36 | 10.32 | 0.14 | 110,284 | 1,263,608 |
| oases | subset\_oases.oases\_assemblies.k61 | 0.73 | 0.65 | 0.07 | 45,388 | 62,872 |
| filter\_illumina | stats\_subset | 0.77 | 0.15 | 0.41 | 1,276 | 16,104 |
| bowtie2-build | bowtie | 2.46 | 2.31 | 0.08 | 101,072 | 176,780 |
| bowtie2 | bowtie | 6.87 | 69.02 | 1.92 | 366,268 | 1,212,016 |
| samtools | bowtie\_to\_bam.bam | 0.95 | 0.90 | 0.03 | 3,780 | 20,556 |
| samtools | bowtie\_to\_bam.sort | 1.33 | 1.25 | 0.05 | 139,500 | 158,372 |
| samtools | bowtie\_to\_bam.index | 0.12 | 0.09 | 0.01 | 1,276 | 19,000 |
| insert\_stats | estimate\_insert | 0.13 | 0.09 | 0.02 | 1,272 | 16,076 |

 Back to TOC

## remove\_rrna (Run 26)

> Assembles and identifies ribosomal RNA (rRNA) sequences, removes read pairs
> that map to these rRNA sequences, and provides a variety of diagnostics about
> rRNA. A single exemplar sequence is presented for each type of rRNA that is
> found, but rRNA read pairs are excluded by mapping to a large set of rRNA
> transcripts that are derived from multiple assemblies over a range of data
> subset sizes.

|  |  |
| --- | --- |
| **Read pairs examined** | 18,806,969 |
| **Read pairs kept** | 13,582,087 |
| **Percent kept** | 72.2% |

#### large-mito-rRNA / no targets

#### large-nuclear-rRNA / 1 target(s) / 802,464 pairs removed (4.27%)

**Locus\_500000.48\_Transcript\_1/1\_Confidence\_1.000\_Length\_3944:**

|  |  |
| --- | --- |
| **Mean coverage** | 5671.9 |
| **Median coverage** | 4,103 |
| **Min coverage** | 1 |
| **Max coverage** | 34,680 |

  
  

```
  >large-nuclear-rRNA|Locus_500000.48_Transcript_1/1_Confidence_1.000_Length_3944|Run26|SRX288276
  TTTCGACCGGCCCCACTTGTGCAACCATTTGTGTATTTTTACGCACTTGTTTTATCATGTATGTTATTAACTATGTTAAT
  AACGTGAAATAGAAACGTGACAACTTCTAACGGTGGATCTCTTGGCTCGTGCATCGATGAAGAACGTAGCCAGTTGCGAT
  AAGTAGTGTGAATTGCAGAATTCAGTGAATCATCGAATCTTTGAACGCAAATTGCGCTTTCTGGATATCCAGGGAGCATG
  CCTGTCTGAGCGTCGTTTAATAATCTACACACCTTGGTGTGGTTAGGGAGCTCATGCAATCTCGTTTGCCTGTCTCTTGA
  TGTATCTGAACCTGTAGAACACTTGTGTACTGCTTGGAAGGCTCAACTTGCTTCTGTCCTTCTAAAGTAACTACTGATAG
  ACCGTTTTGTATGGTTCTTTGAATTCTTTGAATTCACTTCTTGATTTTTGACCTCAGATCAGACAAGACTACCCGCTGAA
  TTTAAGCATATTAATAAGCGGAGGAAAAGAAACTAACAAGGATTCCCCTAGTAACGGCGAGTGAAGCGGGATCAGCTCAA
  ACTTAAAATCTGCGTTGCTTGCAACGCCGAATTGTAGTCTAGAGATTCGTTTTCAAGGCGAATGCGCAGTACTTAAGTTG
  CTTGGAACGGCACATCGTAGAGGGTGACAATCCCGTACGTGGTACTGTGCATCGTTCACGATGCGTTTTCTGTGAGTCGG
  GTTGCTTGGTAATGCAGCCCAAAATTGGAGGTAAACTCCTTCTAAAGCTAAATATTGGCACGAGACCGATAGCGAACAAG
  TACCGTGAGGGAAAGATGAAAAGCACTTTGAAAAGAAAGTTAATAGTACGTGAAACCGTTAGGAGGGAAGCGCATGGAAT
  TAGCAATGCTCTGTCGAGATTCAGACGATCGGCTCTGTTTACTTTGGTCGTACGGATTCGAATGGACCGTTGGCTTTGGT
  CAAGCAGTGCTGTTTGTCGCATTTCCCGGCAGTGTGCGTCAACAGGTATTGGATCTGGGTGATACGCCCCGCAAGAATGT
  GGCAGGCTTCGGTTTGTGTTATAGCTTGTGGTGTGCTAGCTCGGATTCGATAGAGGCGTCGCAGCACATGCCCTCGGGCT
  GGCTTCTGTTTCCTCGGCTCGGTGTGACCATAACGGACTGCATGCAGTGCGTTTGAACTTCATCGGGTCGTCGGGGGCAA
  GAATGCACACTATGTGCTTAGGTTGTTGGCGATCATATGGTTTCATGCGACCCGTCTTGGAACACGGACCAAGGAGTCTA
  ACATGTGTGCGAGTCTTAGGGTGATTGAAACCCGCAGGCACAATGAAAGTAAAGGCTCTTCGGAGCTGAGGTGAGATCTC
  TTCGGTTTCGGCTGGAGAGCGCATCATCGACCGACCTCTTCTAATCCTAGAAAGGTTTGAGTAAGAGCACATCTGTTGGG
  ACCCGAAAGATGGTGAACTATGCTTGAGTAGGGCGAAGCCAGAGGAAACTCTGGTGGAGGCTCGTAGCGATTCTGACGTG
  CAAATCGATCGTCAAACTTGAGTATAGGGGCGAAAGACTAATCGAACCATCTAGTAGCTGGTTCCCTCCGAAGTTTCCCT
  TAGGATAGCTGGAACTCGGAACAGTTTTATCAGGTAAAGCGAATGATTAGAGGTCTTAGGGTTGAAACAACCTTAACCTA
  TTCTCAAACTTTAAATTGGTAAGAAGCCCGACTTGCTTGATTGAAGTAGGGCACAGAATGAGAGTTCTTAGTGGGCCATT
  TTTGGTAAGCAGAACTGGCGATGCGGGATGAACCGAACGCTGAGTTAAGGCGCCTAAATCGACGCTCATCAGACCCCACA
  AAAGGTGTTGGTTGATCCAGACAGCAGGACGGTGGCCATGGAAGTCGGAATCCGCTAAGGAGTGTGTAACAACACACCTG
  CCGAATCAACTAGCCCTGAAAATGGATGGCGCTCAAGCGTCGTGCCTATACTCAGCCGTCAGAGTAAATAGCGAAGCTCT
  GACGAGTAGGAGGGCGTGGGGGTCGTGACGCAGCCTTTGGCGTGAGCCTGGGTGAAACGGCCTCTAGTGAAGATCTTGGT
  GGTAGTAGCAAATATTCAAATGAGAACTTTGAAGACCGAAGTGGAGAAAGGTTCCATGTGAACAGCAGTTGGACATGGGT
  TAGTCGATCCTAAGAGATAGGGAAATTCCGTTTCAAAGTGTCCAATCTTGGACCATCGATCGAAAGGGAATCGGGTTAAA
  ATTCCCGAACCAGAACGTGGATATTGTACCCCTCCGGGGTTTACATGTGCGGCAACGCAACTGAACTCGGAGACGTCGGC
  AGGAGCCCTGGGAAGAGTTCTCTTTTCTTGTTAACGGCCTGACACCATGGAATCTGATTGCCAGGAGATATGGTTTGATG
  GCCGGTAAAGCACCACACTTCATGTGGTGTCCGGTGCGCTCCTGAAGGCCCTTGAAAATCCGAGGGAAAGAGTGATTTTC
  ACGTCTGTTCGTACTCATAACCGCAGCAGGTCTCCAAGGTGAGCAGCCTCTGGTCGATAGAACAATGTAGGTAAGGGAAG
  TCGGCAAAATAGATCCGTAACTTCGGGAAAAGGATTGGCTCTAAGGGTTGGGTCTGTCGGGCTGAGACTTGAAGCGAGTG
  GATCCAACCTGGACTGGCTAGGGCCTCTCGGGGCTTTGGTCGGACTCGGAAGGAACTACTCGTGGATTGGCCCAGCTATG
  CTCGCAAGGGCAGTTCGGCAGGCAATTAACAATCAACTTAGAACTGGTACGGACAAGGGGAATCCGACTGTTTAATTAAA
  ACAAAGCATTGCGATGGCCGGAAACGGTGTTGACGCAATGTGATTTCTGCCCAGTGCTCTGAATGTCAAAGTGAAGAAAT
  TCAACCAAGCGCGGGTAAACGGCGGGAGTAACTATGACTCTCTTAAGGTAGCCAAATGCCTCGTCATCTAATTAGTGACG
  CGCATGAATGGATTAACGAGATTCCCACTGTCCCTATCTACTATCTAGCGAAACCACAGCCAAGGGAACGGGCTTGGCAA
  AATCAGCGGGGAAAGAAGACCCTGTTGAGCTTGACTCTAGTCTGACTTTGTGAAAAGACATAGGAGGTGTAGAATAGGTG
  GGAGCAGTAATGCAAAAGTGAAATACCACTACTCTTATAGTTTTTTTACTTATTCGATTGAGCGGAAGCGAGCTTCACGG
  CTCATTTTCTAGAATTAAGGCCCCGTTGGCGGGTCGATCCGTGTCGAAGACACTGTCAGGTTGGGAGTTTGGCTGGGGCG
  GCACATCTGTCAAATGATAACGCAGGTGTCCTAAGGTGAGCTCAATGAGAACGGAAATCTCATGTAGAACAAAAGGGTAA
  AAGCTCACTTGATTTTGATTTTCAGTATGAATACAAACCGTGAAAGCGTGGCCTATCGATCCTTTAGTCTTTAGGAGTTT
  TTAAGCTAGAGGTGTCAGAAAAGTTACCACAGGGATAACTGGCTTGTGGCAGCCAAGCGTTCATAGCGACGTTGCTTTTT
  GATCCTTCGATGTCGGCTCTTCCTATCATTGCGAAGCAGAATTCGCCAAGTGTTGGATTGTTCACCCACTAATAGGGAAC
  GTGAGCTGGGTTTAGACCGTCGTGAGACAGGTTAGTTTTACCCTACTGATGAAGTGTTGTTGCAATAGTAATTCTGCTCA
  GTACGAGAGGAACCGCAGATTCAGACAATTGGCATTTGCACTTGCTTGAAAAGGCAATGGTGCGAAGCTACCATCTGTTG
  GATTATGACTGAACGCCTCTAAGTCAGAATCCGTGCTAGAAAGCAATGATAATTACCTCTGGATAATCTTAGGCGAATAA
  GAATAGAATGGCTTCGGCTGTTCCTGAATCTCAATGCACTGACTCAGAGAAAAACTGATGAGGTGCTGCAACTATCAAAA
  TCTAATATTTTCAGAGATAAATCC
```

Top NCBI nt hit: gnl|BL\_ORD\_ID|26394 gi|12381826|emb|AJ271116.1| Diphyscium foliosum partial 25S rRNA gene (E-value: 0.0)

#### small-mito-rRNA / no targets

#### small-nuclear-rRNA / 1 target(s) / 4,422,418 pairs removed (23.51%)

**Locus\_5000.1\_Transcript\_1/1\_Confidence\_1.000\_Length\_1850:**

|  |  |
| --- | --- |
| **Mean coverage** | 59844.9 |
| **Median coverage** | 60,442 |
| **Min coverage** | 312 |
| **Max coverage** | 134,904 |

  
  

```
  >small-nuclear-rRNA|Locus_5000.1_Transcript_1/1_Confidence_1.000_Length_1850|Run26|SRX288276
  CCTGCCAGTAGTCATATGCTTGTCTCAAAGATTAAGCCATGCATGTCTAAGTATAAGCACTTGTACTGTGAAACTGCGAA
  TGGCTCATTAAATCAGTTATCGTTTATTTGATTGTACTTTACTACATGGATACCTGTGGTAATTCTAGAGCTAATACATG
  CGAAAAATCCCGACTTCTGGAAGGGATGTATTTATTAGATTAAAAACCAATGCGAGTCTTCGGGCTCGTTTTCTTGGTGA
  TTCATGATAACTTTTCGAATCGCATGGCCTAGCGCCGGCGATATTTCATTCAAATTTCTGCCCTATCAACTGTCGATGGT
  AAGGTAGTGGCTTACCATGGTTATAACGGGTGACGGAGAATTAGGGTTCGATTCCGGAGAGGGAGCCTGAGAAACGGCTA
  CCACATCTAAGGAAGGCAGCAGGCTCGAAAATTACCCAATCCCAATTCGGGGAGGTAGTGACAAGAAATAACGATACGGG
  GTCTTAATAGGTCTCGCAATTGGAATGAGTACAATTTAAATCCTTTAACGAGGATCAATTGGAGGGCAAGTCTGGTGCCA
  GCAGCCGCGGTAATTCCAGCTCCAATAGCGTATATTAAAGTTGTTGCAGTTAAAAAGCTCGTAGTTGGATTTCGGAGTGG
  GCCAGTTGGTCCGCCGCAAGGTGTGTTACTGACTGGTTTGCTCTTCTTCGCAAAGACTGCGTGTGCTCTTTGTTGAGTGT
  GCGTAGGATTTACGACGTTTACTTTGAAAAAATTAGAGTGTTCAAAGCAGGCTATTGCTTGAATACATGAGCATGGAATA
  ATGGAATAGGACTTTGGTCCCATTTTGTTGGTTTCTAGGACCGAAGTAATGATTAAGAGGGACAATTGGGGGCATCCGTA
  TTTCGTTGTCAGAGGTGAAATTCTTGGATTTACGAAAGACGAACAACTGCGAAAGCACTTGCCAAGAGTGTTTTCATTAA
  TCAAGAACGAAAGTTAGAGGATCGAAGACGATCAGATACCGTCCTAGTTCTAACCATAAACGATGTCGACTAGGGATCAG
  CGGGCGTTATCGTACGACCCCGTTGGCACCTTACGGGAAACCAAAGTTTCTGGATTCCGGGGGAAGTATAGTCGCAAGGC
  CGAAACTTAAAGGAATTGACGGAAGGGCACCACCAGGAGTGGAGCCTGTGGCTCAATTTGACCCAACACGGGAAAACTTA
  CCAGGTCCAGACATAGTAAGGATTGACGACGGAAGGGCACCACCAGGAGTGGAGCCTGTGGCTCAATTTGACTCAACACG
  GGAAAACTTACCAGGTCCAGACATAGTAAGGATTGACAGGTTGAGAGCCCTTTCTTGATTCTATGGGTGGTGGTGCATGG
  CCGTTCTTAGTTGGTGGAGTGATTTGTCTGGTTAATTCCGTTAACGAACGAGACCTTAACCGGCTAAATAGTCATGCGAT
  TCTCGAATCGTAACTGACTTCTTAGAGGGACTGTTGGTGTTTAACCAAAGTCAGGAAGGCAATAACAGGTCTGTGATGCC
  CTTAGATGTTCTGGGCCGCACGCGCGCTACACTGTCGGATTCAGCGAGTCTTAACCTTAACCGAAAGGTTTGGGTAATCT
  TTTGAAAGTCCGACGTGATGGGGATTGATCATTGCAATTATTGATCATGAACGAGGAATTCCTAGTAAATGCGAGTCATC
  AGCTCGCGTTGATTACGTCCCTGCCCTTTGTACACACCGCCCGTCGCTACTACCGATTGAATGGTTTAGTGAGATCTTCG
  GATTGGCACAATCGCGGCCTAACGGAAGTGATGGTGCCGAAAAGTTGCTCAAACTTGATCATTTAGAGGAAGTAAAAGTC
  GTAACAAGGA
```

Top NCBI nt hit: gnl|BL\_ORD\_ID|17793 gi|5931757|emb|AJ133553.1| Paracondylactis hertwigi 18S rRNA gene (E-value: 0.0)

#### Resourse Usage

| Wall Time (s) | User Time (s) | System Time (s) | Memory (KB) | Virtual Memory (KB) |
| --- | --- | --- | --- | --- |
 3905.63 [sum] | 25990.44 [sum] | 784.86 [sum] | 2,633,812 [max] | 2,760,584 [max] |

 Show/hide details

| Command | Stage | Wall Time (s) | User Time (s) | System Time (s) | Memory (KB) | Virtual Memory (KB) |
| --- | --- | --- | --- | --- | --- | --- |
| filter\_illumina | subset\_assemblies.500 | 0.06 | 0.01 | 0.02 | 1,288 | 16,104 |
| velveth | subset\_assemblies.500.oases\_assemblies.k61 | 0.08 | 0.45 | 0.10 | 201,016 | 1,572,468 |
| velvetg | subset\_assemblies.500.oases\_assemblies.k61 | 0.27 | 2.86 | 0.04 | 67,060 | 1,417,444 |
| oases | subset\_assemblies.500.oases\_assemblies.k61 | 0.03 | 0.00 | 0.01 | 1,300 | 35,980 |
| filter\_illumina | subset\_assemblies.1000 | 0.08 | 0.01 | 0.05 | 1,284 | 16,104 |
| velveth | subset\_assemblies.1000.oases\_assemblies.k61 | 0.12 | 0.62 | 0.13 | 203,072 | 1,769,344 |
| velvetg | subset\_assemblies.1000.oases\_assemblies.k61 | 0.32 | 3.10 | 0.04 | 67,272 | 1,286,652 |
| oases | subset\_assemblies.1000.oases\_assemblies.k61 | 0.03 | 0.01 | 0.01 | 1,548 | 36,272 |
| filter\_illumina | subset\_assemblies.2500 | 0.16 | 0.04 | 0.10 | 1,284 | 16,104 |
| velveth | subset\_assemblies.2500.oases\_assemblies.k61 | 0.15 | 0.73 | 0.15 | 207,840 | 1,835,424 |
| velvetg | subset\_assemblies.2500.oases\_assemblies.k61 | 0.39 | 3.79 | 0.04 | 67,908 | 1,287,272 |
| oases | subset\_assemblies.2500.oases\_assemblies.k61 | 0.03 | 0.01 | 0.01 | 2,528 | 36,780 |
| filter\_illumina | subset\_assemblies.5000 | 0.40 | 0.08 | 0.21 | 1,284 | 16,104 |
| velveth | subset\_assemblies.5000.oases\_assemblies.k61 | 0.21 | 0.89 | 0.14 | 215,072 | 1,582,692 |
| velvetg | subset\_assemblies.5000.oases\_assemblies.k61 | 0.66 | 5.43 | 0.03 | 68,916 | 1,353,836 |
| oases | subset\_assemblies.5000.oases\_assemblies.k61 | 0.05 | 0.03 | 0.01 | 3,660 | 37,952 |
| filter\_illumina | subset\_assemblies.10000 | 0.88 | 0.18 | 0.41 | 1,284 | 16,104 |
| velveth | subset\_assemblies.10000.oases\_assemblies.k61 | 0.34 | 1.28 | 0.15 | 229,600 | 1,904,044 |
| velvetg | subset\_assemblies.10000.oases\_assemblies.k61 | 0.87 | 7.56 | 0.06 | 77,240 | 1,224,896 |
| oases | subset\_assemblies.10000.oases\_assemblies.k61 | 0.09 | 0.06 | 0.01 | 5,464 | 39,876 |
| filter\_illumina | subset\_assemblies.25000 | 2.05 | 0.44 | 1.02 | 1,284 | 16,104 |
| velveth | subset\_assemblies.25000.oases\_assemblies.k61 | 0.84 | 2.23 | 0.19 | 270,404 | 1,844,608 |
| velvetg | subset\_assemblies.25000.oases\_assemblies.k61 | 1.05 | 5.47 | 0.07 | 77,544 | 1,362,364 |
| oases | subset\_assemblies.25000.oases\_assemblies.k61 | 0.19 | 0.15 | 0.03 | 11,724 | 42,160 |
| filter\_illumina | subset\_assemblies.50000 | 3.98 | 0.85 | 2.02 | 1,284 | 16,104 |
| velveth | subset\_assemblies.50000.oases\_assemblies.k61 | 1.65 | 4.54 | 0.31 | 333,360 | 1,592,208 |
| velvetg | subset\_assemblies.50000.oases\_assemblies.k61 | 1.91 | 7.65 | 0.12 | 88,384 | 1,307,484 |
| oases | subset\_assemblies.50000.oases\_assemblies.k61 | 0.45 | 0.38 | 0.05 | 22,748 | 44,508 |
| filter\_illumina | subset\_assemblies.100000 | 6.48 | 1.72 | 4.10 | 1,284 | 16,104 |
| velveth | subset\_assemblies.100000.oases\_assemblies.k61 | 2.20 | 8.02 | 0.48 | 450,980 | 1,613,168 |
| velvetg | subset\_assemblies.100000.oases\_assemblies.k61 | 3.58 | 10.92 | 0.16 | 110,308 | 1,384,888 |
| oases | subset\_assemblies.100000.oases\_assemblies.k61 | 0.75 | 0.65 | 0.08 | 45,392 | 62,920 |
| filter\_illumina | subset\_assemblies.250000 | 16.24 | 4.17 | 10.24 | 1,284 | 16,104 |
| velveth | subset\_assemblies.250000.oases\_assemblies.k61 | 5.89 | 21.49 | 0.98 | 768,632 | 1,741,320 |
| velvetg | subset\_assemblies.250000.oases\_assemblies.k61 | 9.77 | 28.02 | 0.32 | 175,892 | 1,328,596 |
| oases | subset\_assemblies.250000.oases\_assemblies.k61 | 2.19 | 1.98 | 0.16 | 119,804 | 137,332 |
| filter\_illumina | subset\_assemblies.500000 | 32.08 | 8.41 | 20.64 | 1,284 | 16,104 |
| velveth | subset\_assemblies.500000.oases\_assemblies.k61 | 11.22 | 40.36 | 1.60 | 1,242,436 | 1,845,788 |
| velvetg | subset\_assemblies.500000.oases\_assemblies.k61 | 22.80 | 68.22 | 0.51 | 234,528 | 1,577,968 |
| oases | subset\_assemblies.500000.oases\_assemblies.k61 | 4.90 | 4.44 | 0.38 | 246,144 | 263,672 |
| filter\_illumina | subset\_assemblies.1000000 | 60.11 | 16.57 | 40.65 | 1,288 | 16,104 |
| velveth | subset\_assemblies.1000000.oases\_assemblies.k61 | 23.64 | 82.73 | 2.94 | 1,740,372 | 2,760,584 |
| velvetg | subset\_assemblies.1000000.oases\_assemblies.k61 | 55.83 | 163.31 | 1.05 | 447,680 | 1,597,492 |
| oases | subset\_assemblies.1000000.oases\_assemblies.k61 | 12.03 | 10.99 | 0.88 | 530,376 | 547,836 |
| makeblastdb | blast\_rrna | 0.08 | 0.02 | 0.01 | 8,128 | 45,012 |
| blastn | blast\_rrna | 14.07 | 13.51 | 0.48 | 167,140 | 2,004,124 |
| blastn | blast\_nt | 0.76 | 3.88 | 0.17 | 135,460 | 2,092,452 |
| bowtie2-build | bowtie | 1.01 | 0.69 | 0.08 | 98,680 | 176,008 |
| bowtie2 | bowtie | 1758.89 | 24249.05 | 113.72 | 310,320 | 1,180,376 |
| samtools | bowtie\_to\_bam.bam | 292.45 | 283.52 | 6.94 | 3,364 | 20,108 |
| samtools | bowtie\_to\_bam.sort | 312.05 | 304.33 | 3.05 | 627,636 | 751,256 |
| samtools | bowtie\_to\_bam.index | 16.21 | 15.06 | 0.65 | 896 | 18,748 |
| samtools | bam\_pileup | 437.94 | 430.74 | 2.08 | 61,572 | 82,120 |
| samtools | bam\_extract\_ids.large-nuclear-rRNA | 8.80 | 6.03 | 0.38 | 2,056 | 18,752 |
| samtools | bam\_extract\_ids.small-nuclear-rRNA | 45.64 | 32.61 | 1.52 | 2,044 | 18,748 |
| exclude | exclude\_ids | 730.71 | 130.11 | 565.10 | 2,633,812 | 2,648,688 |

 Back to TOC

## sanitize (Run 28)

> Filters raw paired-end Illumina data to remove very low quality read pairs,
> read pairs with adapter sequences, and read pairs with highly skewed base
> composition. It then randomizes the order of reads in the files (applying
> the same order of randomization to each file in the pair) to make it simple
> to get random subsets of read pairs in later analyses. Finally, fastqc is
> run to profile the quality of the reads.

#### Illumina Filtering

|  |  |
| --- | --- |
| **Read pairs examined** | 21,575,176 |
| **Read pairs kept** | 18,806,969 |
| **Percent kept** | 87.2% |
| **Illumina quality threshold** | 28 |
| **Adapter fails** | 59,900 |
| **Quality fails** | 3,172,739 |
| **Base composition fails** | 471,153 |


#### FastQC reports

|  |  |
| --- | --- |
| 28.fastqc.1  ``` PASS Basic Statistics  PASS Per base sequence quality  PASS Per sequence quality scores  FAIL Per base sequence content  FAIL Per base GC content  PASS Per sequence GC content  PASS Per base N content  PASS Sequence Length Distribution  WARN Sequence Duplication Levels  WARN Overrepresented sequences  WARN Kmer Content ``` | 28.fastqc.2  ``` PASS Basic Statistics  PASS Per base sequence quality  PASS Per sequence quality scores  FAIL Per base sequence content  FAIL Per base GC content  PASS Per sequence GC content  PASS Per base N content  PASS Sequence Length Distribution  WARN Sequence Duplication Levels  WARN Overrepresented sequences  WARN Kmer Content ``` |

> FastQC is a tool from Babraham Bioinformatics that generates detailed
> quality diagnostics of NGS sequence data.

#### Resourse Usage

| Wall Time (s) | User Time (s) | System Time (s) | Memory (KB) | Virtual Memory (KB) |
| --- | --- | --- | --- | --- |
 1254.32 [sum] | 350.74 [sum] | 794.45 [sum] | 8,782,796 [max] | 9,019,704 [max] |

 Show/hide details

| Command | Stage | Wall Time (s) | User Time (s) | System Time (s) | Memory (KB) | Virtual Memory (KB) |
| --- | --- | --- | --- | --- | --- | --- |
| randomize | randomize | 114.10 | 43.68 | 17.91 | 8,782,796 | 9,019,704 |
| fastqc | fastqc | 7.76 | 8.02 | 0.44 | 591,424 | 8,162,540 |
| filter\_illumina | sanitize | 1132.46 | 299.04 | 776.10 | 1,328 | 16,104 |

 Back to TOC

## insert\_size (Run 29)

> Estimates the insert size distribution of paired-end Illumina data by assembling a subset
> of the data and mapping read pairs to it. The insert size does not include the adapters
> added during library preparation.

|  |  |
| --- | --- |
| **Mean insert size (bp)** | 291.86 |
| **Standard deviation (bp)** | 44.06 |

> A histogram of insert sizes.

#### Resourse Usage

| Wall Time (s) | User Time (s) | System Time (s) | Memory (KB) | Virtual Memory (KB) |
| --- | --- | --- | --- | --- |
 65.86 [sum] | 185.33 [sum] | 11.11 [sum] | 564,756 [max] | 2,006,384 [max] |

 Show/hide details

| Command | Stage | Wall Time (s) | User Time (s) | System Time (s) | Memory (KB) | Virtual Memory (KB) |
| --- | --- | --- | --- | --- | --- | --- |
| filter\_illumina | subset | 6.62 | 1.75 | 4.14 | 1,288 | 16,104 |
| velveth | subset\_oases.oases\_assemblies.k31 | 3.02 | 12.47 | 0.71 | 564,756 | 1,613,168 |
| velvetg | subset\_oases.oases\_assemblies.k31 | 9.26 | 24.72 | 0.39 | 175,340 | 1,325,556 |
| oases | subset\_oases.oases\_assemblies.k31 | 2.07 | 1.39 | 0.13 | 61,012 | 78,500 |
| velveth | subset\_oases.oases\_assemblies.k41 | 2.85 | 10.82 | 0.65 | 531,852 | 2,006,384 |
| velvetg | subset\_oases.oases\_assemblies.k41 | 6.40 | 17.55 | 0.25 | 137,660 | 1,517,804 |
| oases | subset\_oases.oases\_assemblies.k41 | 1.68 | 1.16 | 0.08 | 55,520 | 72,988 |
| velveth | subset\_oases.oases\_assemblies.k51 | 2.71 | 9.39 | 0.63 | 493,640 | 1,940,844 |
| velvetg | subset\_oases.oases\_assemblies.k51 | 4.48 | 11.76 | 0.23 | 110,576 | 1,319,940 |
| oases | subset\_oases.oases\_assemblies.k51 | 1.39 | 0.82 | 0.12 | 49,076 | 66,600 |
| velveth | subset\_oases.oases\_assemblies.k61 | 2.66 | 8.22 | 0.52 | 451,348 | 2,006,380 |
| velvetg | subset\_oases.oases\_assemblies.k61 | 3.71 | 11.10 | 0.18 | 118,532 | 1,319,200 |
| oases | subset\_oases.oases\_assemblies.k61 | 1.17 | 0.65 | 0.08 | 45,516 | 62,992 |
| filter\_illumina | stats\_subset | 1.08 | 0.17 | 0.41 | 1,276 | 16,104 |
| bowtie2-build | bowtie | 3.66 | 2.79 | 0.07 | 101,224 | 176,856 |
| bowtie2 | bowtie | 8.97 | 68.32 | 2.38 | 352,916 | 1,192,072 |
| samtools | bowtie\_to\_bam.bam | 1.42 | 0.78 | 0.04 | 3,820 | 20,468 |
| samtools | bowtie\_to\_bam.sort | 1.71 | 1.28 | 0.06 | 137,868 | 158,516 |
| samtools | bowtie\_to\_bam.index | 0.51 | 0.10 | 0.01 | 1,320 | 19,120 |
| insert\_stats | estimate\_insert | 0.50 | 0.10 | 0.02 | 1,272 | 16,076 |

 Back to TOC

## remove\_rrna (Run 30)

> Assembles and identifies ribosomal RNA (rRNA) sequences, removes read pairs
> that map to these rRNA sequences, and provides a variety of diagnostics about
> rRNA. A single exemplar sequence is presented for each type of rRNA that is
> found, but rRNA read pairs are excluded by mapping to a large set of rRNA
> transcripts that are derived from multiple assemblies over a range of data
> subset sizes.

|  |  |
| --- | --- |
| **Read pairs examined** | 18,806,969 |
| **Read pairs kept** | 13,690,073 |
| **Percent kept** | 72.8% |

#### large-mito-rRNA / no targets

#### large-nuclear-rRNA / 1 target(s) / 1,952,236 pairs removed (10.38%)

**Locus\_100000.54\_Transcript\_1/1\_Confidence\_1.000\_Length\_1645:**

|  |  |
| --- | --- |
| **Mean coverage** | 6198.6 |
| **Median coverage** | 4,961 |
| **Min coverage** | 9 |
| **Max coverage** | 21,807 |

  
  

```
  >large-nuclear-rRNA|Locus_100000.54_Transcript_1/1_Confidence_1.000_Length_1645|Run30|SRX288276
  AGGAGATATGGTTTGATGGCCGGTAAAGCACCACACTTCATGTGGTGTCCGGTGCGCTCCTGAAGGCCCTTGAAAATCCG
  AGGGAAAGAGTGATTTTCACGTCTGTTCGTACTCATAACCGCAGCAGGTCTCCAAGGTGAGCAGCCTCTGGTCGATAGAA
  CAATGTAGGTAAGGGAAGTCGGCAAAATAGATCCGTAACTTCGGGAAAAGGATTGGCTCTAAGGGTTGGGTCTGTCGGGC
  TGAGACTTGAAGCGAGTGGATCCAACCTGGACTGGCTAGGGCCTCTCGGGGCTTTGGTCGGACTCGGAAGGAACTACTCG
  TGGATTGGCCCAGCTATGCTCGCAAGGGCAGTTCGGCAGGCAATTAACAATCAACTTAGAACTGGTACGGACAAGGGGAA
  TCCGACTGTTTAATTAAAACAAAGCATTGCGATGGCCGGAAACGGTGTTGACGCAATGTGATTTCTGCCCAGTGCTCTGA
  ATGTCAAAGTGAAGAAATTCAACCAAGCGCGGGTAAACGGCGGGAGTAACTATGACTCTCTTAAGGTAGCCAAATGCCTC
  GTCATCTAATTAGTGACGCGCATGAATGGATTAACGAGATTCCCACTGTCCCTATCTACTATCTAGCGAAACCACAGCCA
  AGGGAACGGGCTTGGCAAAATCAGCGGGGAAAGAAGACCCTGTTGAGCTTGACTCTAGTCTGACTTTGTGAAAAGACATA
  GGAGGTGTAGAATAGGTGGGAGCAGTAATGCAAAAGTGAAATACCACTACTCTTATAGTTTTTTTACTTATTCGATTGAG
  CGGAAGCGAGCTTCACGGCTCATTTTCTAGAATTAAGGCCCCGTTGGCGGGTCGATCCGTGTCGAAGACACTGTCAGGTT
  GGGAGTTTGGCTGGGGCGGCACATCTGTCAAATGATAACGCAGGTGTCCTAAGGTGAGCTCAATGAGAACGGAAATCTCA
  TGTAGAACAAAAGGGTAAAAGCTCACTTGATTTTGATTTTCAGTATGAATACAAACCGTGAAAGCGTGGCCTATCGATCC
  TTTAGTCTTTAGGAGTTTTTAAGCTAGAGGTGTCAGAAAAGTTACCACAGGGATAACTGGCTTGTGGCAGCCAAGCGTTC
  ATAGCGACGTTGCTTTTTGATCCTTCGATGTCGGCTCTTCCTATCATTGCGAAGCAGAATTCGCCAAGTGTTGGATTGTT
  CACCCACTAATAGGGAACGTGAGCTGGGTTTAGACCGTCGTGAGACAGGTTAGTTTTACCCTACTGATGAAGTGTTGTTG
  CAATAGTAATTCTGCTCAGTACGAGAGGAACCGCAGATTCAGACAATTGGCATTTGCACTTGCTTGAAAAGGCAATGGTG
  CGAAGCTACCATCTGTTGGATTATGACTGAACGCCTCTAAGTCAGAATCCGTGCTAGAAAGCAATGATAATTACCTCTGG
  ATAATCTTAGGCGAATAAGAATAGAATGGCTTCGGCTGTTCCTGAATCTCAATGCACTGACTCAGAGAAAAACTGATGAG
  GTGCTGCAACTATCAAAATCTAATATTTTCAGAGATAAATCCTATGCAGACGACTTAAACAAGAACGTGGTATTGTAAAA
  AGCAGAGTAGCCTCTGTGCTACGATCTTCTGAGATTAAGCCTCTG
```

Top NCBI nt hit: gnl|BL\_ORD\_ID|42700 gi|28375676|dbj|AB101601.1| Anadara antiquata gene for ITS2, 28S rRNA, partial sequence, country:Philippines: Mactan (E-value: 0.0)

#### small-mito-rRNA / no targets

#### small-nuclear-rRNA / 1 target(s) / 3,164,660 pairs removed (16.83%)

**Locus\_50000.1\_Transcript\_1/1\_Confidence\_1.000\_Length\_1812:**

|  |  |
| --- | --- |
| **Mean coverage** | 30137.8 |
| **Median coverage** | 30,163 |
| **Min coverage** | 92 |
| **Max coverage** | 62,918 |

  
  

```
  >small-nuclear-rRNA|Locus_50000.1_Transcript_1/1_Confidence_1.000_Length_1812|Run30|SRX288276
  CACCTACGGAAACCTTGTTACGACTTTTACTTCCTCTAAATGATCAAGTTTGAGCAACTTTTCGGCACCATCACTTCCGT
  TAGGCCGCGATTGTGCCAATCCGAAGATCTCACTAAACCATTCAATCGGTAGTAGCGACGGGCGGTGTGTACAAAGGGCA
  GGGACGTAATCAACGCGAGCTGATGACTCGCATTTACTAGGAATTCCTCGTTCATGATCAATAATTGCAATGATCAATCC
  CCATCACGTCGGACTTTCAAAAGATTACCCAAACCTTTCGGTTAAGGTTAAGACTCGCTGAATCCGACAGTGTAGCGCGC
  GTGCGGCCCAGAACATCTAAGGGCATCACAGACCTGTTATTGCCTTCCTGACTTTGGTTAAACACCAACAGTCCCTCTAA
  GAAGTCAGTTACGATTCGAGAATCGCATGACTATTTAGCCGGTTAAGGTCTCGTTCGTTAACGGAATTAACCAGACAAAT
  CACTCCACCAACTAAGAACGGCCATGCACCACCACCCATAGAATCAAGAAAGGGCTCTCAACCTGTCAATCCTTACTATG
  TCTGGACCTGGTAAGTTTTCCCGTGTTGGGTCAAATTGAGCCACAGGCTCCACTCCTGGTGGTGCCCTTCCGTCAATTCC
  TTTAAGTTTCGGCCTTGCGACTATACTTCCCCCGGAATCCAGAAACTTTGGTTTCCCGTAAGGTGCCAACGGGGTCGTAC
  GATAACGCCCGCTGATCCCTAGTCGACATCGTTTATGGTTAGAACTAGGACGGTATCTGATCGTCTTCGATCCTCTAACT
  TTCGTTCTTGATTAATGAAAACACTCTTGGCAAGTGCTTTCGCAGTTGTTCGTCTTTCGTAAATCCAAGAATTTCACCTC
  TGACAACGAAATACGGATGCCCCCAATTGTCCCTCTTAATCATTACTTCGGTCCTAGAAACCAACAAAATGGGACCAAAG
  TCCTATTCCATTATTCCATGCTCATGTATTCAAGCAATAGCCTGCTTTGAACACTCTAATTTTTTCAAAGTAAACGTCGT
  AAATCCTACGCACACTCAACAAAGAGCACACGCAGTCTTTGCGAAGAAGAGCAAACCAGTCAGTAACACACCTTGCGGCG
  GACCAACTGGCCCACTCCGAAATCCAACTACGAGCTTTTTAACTGCAACAACTTTAATATACGCTATTGGAGCTGGAATT
  ACCGCGGCTGCTGGCACCAGACTTGCCCTCCAATTGATCCTCGTTAAAGGATTTAAATTGTACTCATTCCAATTGCGAGA
  CCTATTAAGACCCCGTATCGTTATTTCTTGTCACTACCTCCCCGAATTGGGATTGGGTAATTTTCGAGCCTGCTGCCTTC
  CTTAGATGTGGTAGCCGTTTCTCAGGCTCCCTCTCCGGAATCGAACCCTAATTCTCCGTCACCCGTTATAACCATGGTAA
  GCCACTACCTTACCATCGACAGTTGATAGGGCAGAAATTTGAATGAAATATCGCCGGCGCTAGGCCATGCGATTCGAAAA
  GTTATCATGAATCACCAAGAAAACGAGCCCGAAGACTCGCATTGGTTTTTAATCTAATAAATACATCCCTTCCAGAAGTC
  GGGATTTTTCGCATGTATTAGCTCTAGAATTACCACAGGTATCCATGTAGTAAAGTACAATCAAATAAACGATAACTGAT
  TTAATGAGCCATTCGCAGTTTCACAGTACAAGTGCTTATACTTAGACATGCATGGCTTAATCTTTGAGACAAGCATGTCT
  AAGTATAAGCACTTGTACTGTGAAACTGCGAATGGCTCATTAAATCAGTTAT
```

Top NCBI nt hit: gnl|BL\_ORD\_ID|10788 gi|2369808|emb|Z92905.1| Flosmaris mutsuensis 18S rRNA gene (E-value: 0.0)

#### Resourse Usage

| Wall Time (s) | User Time (s) | System Time (s) | Memory (KB) | Virtual Memory (KB) |
| --- | --- | --- | --- | --- |
 3408.46 [sum] | 21607.95 [sum] | 791.69 [sum] | 2,581,176 [max] | 2,695,048 [max] |

 Show/hide details

| Command | Stage | Wall Time (s) | User Time (s) | System Time (s) | Memory (KB) | Virtual Memory (KB) |
| --- | --- | --- | --- | --- | --- | --- |
| filter\_illumina | subset\_assemblies.500 | 0.42 | 0.01 | 0.03 | 1,284 | 16,104 |
| velveth | subset\_assemblies.500.oases\_assemblies.k61 | 0.51 | 0.69 | 0.13 | 201,076 | 1,965,684 |
| velvetg | subset\_assemblies.500.oases\_assemblies.k61 | 0.59 | 0.72 | 0.06 | 67,068 | 1,482,980 |
| oases | subset\_assemblies.500.oases\_assemblies.k61 | 0.43 | 0.00 | 0.01 | 1,304 | 35,984 |
| filter\_illumina | subset\_assemblies.1000 | 0.43 | 0.02 | 0.05 | 1,288 | 16,104 |
| velveth | subset\_assemblies.1000.oases\_assemblies.k61 | 0.54 | 0.72 | 0.13 | 203,004 | 1,965,436 |
| velvetg | subset\_assemblies.1000.oases\_assemblies.k61 | 0.68 | 1.95 | 0.08 | 67,280 | 1,548,800 |
| oases | subset\_assemblies.1000.oases\_assemblies.k61 | 0.45 | 0.01 | 0.01 | 1,556 | 36,276 |
| filter\_illumina | subset\_assemblies.2500 | 0.53 | 0.05 | 0.11 | 1,284 | 16,104 |
| velveth | subset\_assemblies.2500.oases\_assemblies.k61 | 0.59 | 0.89 | 0.16 | 207,776 | 1,573,280 |
| velvetg | subset\_assemblies.2500.oases\_assemblies.k61 | 0.81 | 4.06 | 0.04 | 67,908 | 1,352,804 |
| oases | subset\_assemblies.2500.oases\_assemblies.k61 | 0.45 | 0.02 | 0.01 | 2,524 | 36,780 |
| filter\_illumina | subset\_assemblies.5000 | 0.75 | 0.10 | 0.21 | 1,284 | 16,104 |
| velveth | subset\_assemblies.5000.oases\_assemblies.k61 | 0.58 | 0.87 | 0.16 | 215,164 | 1,639,456 |
| velvetg | subset\_assemblies.5000.oases\_assemblies.k61 | 0.89 | 3.32 | 0.07 | 68,940 | 1,484,908 |
| oases | subset\_assemblies.5000.oases\_assemblies.k61 | 0.50 | 0.03 | 0.02 | 3,656 | 29,756 |
| filter\_illumina | subset\_assemblies.10000 | 1.06 | 0.20 | 0.40 | 1,288 | 16,104 |
| velveth | subset\_assemblies.10000.oases\_assemblies.k61 | 0.67 | 1.23 | 0.22 | 229,460 | 1,598,036 |
| velvetg | subset\_assemblies.10000.oases\_assemblies.k61 | 0.98 | 3.85 | 0.09 | 71,156 | 1,373,908 |
| oases | subset\_assemblies.10000.oases\_assemblies.k61 | 0.52 | 0.06 | 0.02 | 5,476 | 39,880 |
| filter\_illumina | subset\_assemblies.25000 | 2.00 | 0.43 | 1.06 | 1,284 | 16,104 |
| velveth | subset\_assemblies.25000.oases\_assemblies.k61 | 1.01 | 2.47 | 0.28 | 270,392 | 1,639,220 |
| velvetg | subset\_assemblies.25000.oases\_assemblies.k61 | 1.41 | 5.80 | 0.10 | 77,572 | 1,244,368 |
| oases | subset\_assemblies.25000.oases\_assemblies.k61 | 0.62 | 0.15 | 0.03 | 11,740 | 42,176 |
| filter\_illumina | subset\_assemblies.50000 | 3.48 | 0.88 | 2.07 | 1,284 | 16,104 |
| velveth | subset\_assemblies.50000.oases\_assemblies.k61 | 1.49 | 4.14 | 0.40 | 333,564 | 1,592,208 |
| velvetg | subset\_assemblies.50000.oases\_assemblies.k61 | 2.13 | 7.55 | 0.13 | 88,372 | 1,215,032 |
| oases | subset\_assemblies.50000.oases\_assemblies.k61 | 0.81 | 0.31 | 0.05 | 22,692 | 44,452 |
| filter\_illumina | subset\_assemblies.100000 | 6.56 | 1.69 | 4.25 | 1,284 | 16,104 |
| velveth | subset\_assemblies.100000.oases\_assemblies.k61 | 2.73 | 8.26 | 0.54 | 451,360 | 1,678,704 |
| velvetg | subset\_assemblies.100000.oases\_assemblies.k61 | 3.58 | 9.08 | 0.18 | 112,368 | 1,647,412 |
| oases | subset\_assemblies.100000.oases\_assemblies.k61 | 1.16 | 0.66 | 0.10 | 45,880 | 63,380 |
| filter\_illumina | subset\_assemblies.250000 | 15.50 | 4.17 | 10.37 | 1,284 | 16,104 |
| velveth | subset\_assemblies.250000.oases\_assemblies.k61 | 5.39 | 19.39 | 1.04 | 770,428 | 1,675,840 |
| velvetg | subset\_assemblies.250000.oases\_assemblies.k61 | 9.57 | 25.69 | 0.42 | 176,060 | 1,328,592 |
| oases | subset\_assemblies.250000.oases\_assemblies.k61 | 2.64 | 1.96 | 0.23 | 119,748 | 137,276 |
| filter\_illumina | subset\_assemblies.500000 | 34.29 | 8.19 | 20.83 | 1,284 | 16,104 |
| velveth | subset\_assemblies.500000.oases\_assemblies.k61 | 10.84 | 39.77 | 1.95 | 1,243,356 | 1,780,248 |
| velvetg | subset\_assemblies.500000.oases\_assemblies.k61 | 21.69 | 60.94 | 0.75 | 235,640 | 1,385,288 |
| oases | subset\_assemblies.500000.oases\_assemblies.k61 | 5.25 | 4.33 | 0.40 | 244,780 | 262,304 |
| filter\_illumina | subset\_assemblies.1000000 | 59.99 | 16.56 | 41.63 | 1,284 | 16,104 |
| velveth | subset\_assemblies.1000000.oases\_assemblies.k61 | 20.13 | 83.07 | 3.01 | 1,742,176 | 2,695,048 |
| velvetg | subset\_assemblies.1000000.oases\_assemblies.k61 | 50.19 | 141.10 | 1.08 | 447,792 | 1,595,796 |
| oases | subset\_assemblies.1000000.oases\_assemblies.k61 | 12.52 | 10.85 | 1.05 | 528,692 | 546,164 |
| makeblastdb | blast\_rrna | 2.70 | 0.01 | 0.09 | 8,136 | 44,992 |
| blastn | blast\_rrna | 19.03 | 12.71 | 0.64 | 164,888 | 2,004,128 |
| blastn | blast\_nt | 57.13 | 2.76 | 1.46 | 119,896 | 2,076,060 |
| bowtie2-build | bowtie | 1.55 | 0.66 | 0.08 | 98,676 | 176,000 |
| bowtie2 | bowtie | 1333.96 | 20045.12 | 103.65 | 799,780 | 1,169,728 |
| samtools | bowtie\_to\_bam.bam | 291.72 | 276.70 | 6.90 | 3,356 | 20,104 |
| samtools | bowtie\_to\_bam.sort | 306.70 | 296.84 | 3.30 | 628,052 | 751,032 |
| samtools | bowtie\_to\_bam.index | 16.31 | 15.12 | 0.74 | 900 | 18,748 |
| samtools | bam\_pileup | 316.95 | 311.13 | 2.06 | 57,440 | 74,252 |
| samtools | bam\_extract\_ids.large-nuclear-rRNA | 18.17 | 14.19 | 1.30 | 2,052 | 18,752 |
| samtools | bam\_extract\_ids.small-nuclear-rRNA | 29.23 | 23.10 | 2.15 | 2,048 | 18,752 |
| exclude | exclude\_ids | 727.65 | 133.35 | 575.32 | 2,581,176 | 2,596,024 |

 Back to TOC

## assemble (Run 44)

> Assembles reads into transcripts, processes the assembly, and generates
> assembly diagnostics. Read pairs are first filtered at a more stringent
> mean quality threshold. Assemblies are then performed over a range of
> data subset sizes, which provides an indication of how sequencing effort
> impacts assembly results.

#### Illumina Filtering

|  |  |
| --- | --- |
| **Read pairs examined** | 13,690,073 |
| **Read pairs kept** | 11,673,891 |
| **Percent kept** | 85.3% |
| **Illumina quality threshold** | 33 |
| **Adapter fails** | 0 |
| **Quality fails** | 2,669,619 |
| **Base composition fails** | 0 |

#### Resourse Usage

| Wall Time (s) | User Time (s) | System Time (s) | Memory (KB) | Virtual Memory (KB) |
| --- | --- | --- | --- | --- |
 17523.89 [sum] | 121891.25 [sum] | 6562.92 [sum] | 36,889,628 [max] | 38,507,308 [max] |

 Show/hide details

| Command | Stage | Wall Time (s) | User Time (s) | System Time (s) | Memory (KB) | Virtual Memory (KB) |
| --- | --- | --- | --- | --- | --- | --- |
| filter\_illumina | quality\_filter | 312.35 | 44.29 | 265.19 | 1,336 | 16,100 |
| trinity | trinity.11673891 | 11485.03 | 51534.81 | 2833.78 | 36,889,628 | 38,507,308 |
| parallel | trinity.11673891 | 5726.51 | 70312.15 | 3463.95 | 1,393,060 | 5,237,244 |

 Back to TOC

## postassemble (Run 47)

> Cleans transcripts to remove any rRNA or vector sequences, then selects a
> single exemplar transcript for each gene. Vector sequences could include
> untrimmed adapters or plasmids (we sometimes find sequences in our data for the
> protein expression vectors used to manufacture the sample preparation enzymes).
> Raw reads are mapped back to the exemplars to estimate coverage and assign RPKM
> values. Finally, transcripts are annotated with blastx hits against SwissProt.

#### Assemblies

| Method | Read pairs | Genes | Mean Length (bp) | N50 Length (bp) | Links to assembly files |
| --- | --- | --- | --- | --- | --- |
| trinity | 11,673,891 | 58645 | 756.826771251 | 886 | [annotated transcripts], [rrna], [vectors] |

##### trinity

|  |  |
| --- | --- |
| **Read pairs** | 11,673,891 |
| **Pairs mapped** | 62.9% |
| **Pairs discordant** | 2.7% |
| **Unpaired reads mapped** | 71.3% |

Number of exemplar transcripts in full assembly with blastx hits: 15,696


#### Resourse Usage

| Wall Time (s) | User Time (s) | System Time (s) | Memory (KB) | Virtual Memory (KB) |
| --- | --- | --- | --- | --- |
 10934.14 [sum] | 167329.55 [sum] | 412.53 [sum] | 383,880 [max] | 1,248,032 [max] |

 Show/hide details

| Command | Stage | Wall Time (s) | User Time (s) | System Time (s) | Memory (KB) | Virtual Memory (KB) |
| --- | --- | --- | --- | --- | --- | --- |
| makeblastdb | clean\_rrna.assembly\_11673891\_trinity | 0.03 | 0.01 | 0.01 | 8,164 | 45,008 |
| blastn | clean\_rrna.assembly\_11673891\_trinity | 11.56 | 80.88 | 16.70 | 161,264 | 220,136 |
| blastn | clean\_univec.assembly\_11673891\_trinity | 12.20 | 82.86 | 21.89 | 162,248 | 222,056 |
| dustmasker | dustmasker.assembly\_11673891\_trinity | 14.98 | 14.04 | 0.80 | 8,272 | 43,708 |
| bowtie2-build | coverage.assembly\_11673891\_trinity | 37.51 | 36.80 | 0.53 | 198,420 | 243,296 |
| bowtie2 | coverage.assembly\_11673891\_trinity | 405.10 | 6217.04 | 62.12 | 383,880 | 1,248,032 |
| coverage | coverage.assembly\_11673891\_trinity | 57.82 | 23.45 | 3.24 | 9,044 | 24,916 |
| blastx | nr\_annotate.assembly\_11673891\_trinity | 10394.94 | 160874.48 | 307.25 | 220,024 | 366,396 |

 Back to TOC
