# Supplementary material for: Agalma: an automated phylogenomics workflow
Source: BMC Bioinformatics. 2013 Nov 19;14:330. doi: 10.1186/1471-2105-14-330 (PMC3840672; doi:10.1186/1471-2105-14-330)
Supplement: Additional file 1 — HTML report for assembly of the sample data sets. The HTML report for the assembly of the test data sets from raw reads. The tabular report (index.html) provides an overview across the five assemblies for the ingroup taxa, and includes links (in the Catalog ID column) to detailed reports for the assembly of each species. Fasta files for the annotated transcripts have been removed from the report to reduce file size. [file 1471-2105-14-330-S1.zip › tabular/SRX288432/10.fastqc.2/fastqc_report.html]

SRX288432.random100k.2.fastq FastQC Report


FastQC Report

Tue 11 Jun 2013  
SRX288432.random100k.2.fastq

## Summary

- Basic Statistics
- Per base sequence quality
- Per sequence quality scores
- Per base sequence content
- Per base GC content
- Per sequence GC content
- Per base N content
- Sequence Length Distribution
- Sequence Duplication Levels
- Overrepresented sequences
- Kmer Content

## Basic Statistics

| Measure | Value |
| --- | --- |
| Filename | SRX288432.random100k.2.fastq |
| File type | Conventional base calls |
| Encoding | Sanger / Illumina 1.9 |
| Total Sequences | 100000 |
| Filtered Sequences | 0 |
| Sequence length | 100 |
| %GC | 39 |

## Per base sequence quality

## Per sequence quality scores

## Per base sequence content

## Per base GC content

## Per sequence GC content

## Per base N content

## Sequence Length Distribution

## Sequence Duplication Levels

## Overrepresented sequences

| Sequence | Count | Percentage | Possible Source |
| --- | --- | --- | --- |
| CTCCAATTGATCCTCGTTAAAGGATTTAAATTGTACTCATTCCAATTGCG | 229 | 0.22899999999999998 | No Hit |
| CTTACCAGGTCCAGACATAGTAAGGATTGACAGGTTGAGAGCCCTTTCTT | 224 | 0.22399999999999998 | No Hit |
| CCTGCCAGTAGTCATATGCTTGTCTCAAAGATTAAGCCATGCATGTCTAA | 197 | 0.197 | No Hit |
| CCCCAATTGTCCCTCTTAATCATTACTTCGGTCCTAGAAACCAACAAAAT | 181 | 0.181 | No Hit |
| CTCGCTGAATCCGACAGTGTAGCGCGCGTGCGGCCCAGAACATCTAAGGG | 134 | 0.134 | No Hit |
| CAGAAATTTGAATGAAATATCGCCGGCGCTAGGCCATGCGATTCGAAAAG | 131 | 0.131 | No Hit |
| AAACTTACCAGGTCCAGACATAGTAAGGATTGACAGGTTGAGAGCCCTTT | 129 | 0.129 | No Hit |
| CCTCGTTCATGATCAATAATTGCAATGATCAATCCCCATCACGTCGGACT | 127 | 0.127 | No Hit |
| CTGCCAGTAGTCATATGCTTGTCTCAAAGATTAAGCCATGCATGTCTAAG | 121 | 0.121 | No Hit |
| CGGCCTTGCGACTATACTTCCCCCGGAATCCAGAAACTTTGGTTTCCCGT | 107 | 0.107 | No Hit |

## Kmer Content

| Sequence | Count | Obs/Exp Overall | Obs/Exp Max | Max Obs/Exp Position |
| --- | --- | --- | --- | --- |
| GGTGG | 11575 | 2.5908194 | 5.758634 | 60-64 |
| CTTCC | 12400 | 1.6552429 | 6.471361 | 1 |
| CCCCC | 5145 | 1.6061713 | 12.43709 | 1 |
| GGTCC | 7350 | 1.5711117 | 5.540484 | 8 |
| CTCCA | 10695 | 1.5038173 | 11.810997 | 1 |
| CTGGT | 10390 | 1.4522856 | 6.3737397 | 1 |
| CCTCC | 6945 | 1.4177364 | 5.0951953 | 1 |
| CCCCG | 4415 | 1.4103776 | 9.660052 | 1 |
| TCCAA | 13915 | 1.3476855 | 5.764495 | 2 |
| CCAGG | 5830 | 1.3126909 | 5.403786 | 5 |
| CTGGA | 8725 | 1.2846245 | 5.088356 | 1 |
| CCCCA | 5965 | 1.2826493 | 11.662997 | 1 |
| CCCTC | 6280 | 1.2819848 | 7.3488393 | 1 |
| CGCCG | 3885 | 1.2699715 | 5.648585 | 1 |
| CCAAT | 12995 | 1.2585821 | 7.159129 | 3 |
| CTTGT | 14075 | 1.2571973 | 5.0590763 | 1 |
| CCTGC | 5985 | 1.2502176 | 7.219185 | 1 |
| CTCGT | 9075 | 1.2396096 | 6.031986 | 1 |
| CCCAA | 8275 | 1.2256219 | 5.687386 | 1 |
| AATTG | 18785 | 1.217395 | 5.661427 | 5 |
| CTGCC | 5780 | 1.207395 | 5.11359 | 1 |
| CAATT | 19035 | 1.2055216 | 5.5021815 | 4 |
| CCTGG | 5425 | 1.15963 | 5.5404835 | 1 |
| CTTGG | 8235 | 1.1510656 | 5.3002677 | 1 |
| CCCGT | 5460 | 1.1405494 | 6.5173197 | 1 |
| GCGCG | 3315 | 1.1088803 | 6.2618113 | 2 |
| CTCCC | 5270 | 1.0758058 | 7.5448084 | 1 |
| CCTCG | 5075 | 1.0601261 | 5.6149216 | 1 |
| CGGCC | 3120 | 1.0198998 | 5.1778693 | 1 |
| CTCGC | 4865 | 1.0162588 | 8.322117 | 1 |
| GTCCC | 4795 | 1.0016363 | 5.013323 | 9 |
| CTCTG | 7320 | 0.99988335 | 5.1140747 | 1 |
| CTCCT | 7460 | 0.99581546 | 7.9450374 | 1 |
| CCCAC | 4545 | 0.9773078 | 5.470255 | 1 |
| CCCGG | 2875 | 0.93981147 | 6.2762046 | 1 |
| CTCCG | 4055 | 0.84705645 | 8.322117 | 1 |
| CCCTG | 3930 | 0.82094485 | 5.314122 | 1 |
| CCCCT | 3895 | 0.79511636 | 7.15287 | 1 |
| CGCGG | 2030 | 0.6790428 | 5.1378965 | 1 |
| CGGGC | 1820 | 0.608797 | 6.101252 | 1 |

Produced by FastQC (version 0.10.1)
