# Supplementary material for: Agalma: an automated phylogenomics workflow
Source: BMC Bioinformatics. 2013 Nov 19;14:330. doi: 10.1186/1471-2105-14-330 (PMC3840672; doi:10.1186/1471-2105-14-330)
Supplement: Additional file 1 — HTML report for assembly of the sample data sets. The HTML report for the assembly of the test data sets from raw reads. The tabular report (index.html) provides an overview across the five assemblies for the ingroup taxa, and includes links (in the Catalog ID column) to detailed reports for the assembly of each species. Fasta files for the annotated transcripts have been removed from the report to reduce file size. [file 1471-2105-14-330-S1.zip › tabular/SRX288432/index.html]

Craseoa lathetica


# *Craseoa lathetica*

> |  |  |
> | --- | --- |
> | **id** | SRX288432 |
> | **ncbi\_id** | 316205 |
> | **library\_id** | SRR871529 |
> | **library\_type** | TRANSCRIPTOMIC |
> | **sequencer** | Illumina HiSeq 2000 |
> | **seq\_center** | Dunnlab |
> | **sample\_prep** | Invitrogen Dynabeads mRNA DIRECT kit ; 1 round | Illumina TruSeq RNA Sample Prep Kit |

## Table of Contents

|  |  |  |
| --- | --- | --- |
| **Run 10** *sanitize* 2013-06-11T12:34:57.918478 node425 | - sanitize | ``` Wall Time (s)      :      1560.75 [sum] User Time (s)      :       476.97 [sum] System Time (s)    :       856.27 [sum] Memory (KB)        :       36,512 [max] Virtual Memory (KB):      297,840 [max] ``` |
| **Run 12** *insert\_size* 2013-06-11T13:01:01.124350 node425 | - insert\_size | ``` Wall Time (s)      :        30.08 [sum] User Time (s)      :        90.42 [sum] System Time (s)    :         6.44 [sum] Memory (KB)        :       43,808 [max] Virtual Memory (KB):      308,976 [max] ``` |
| **Run 13** *remove\_rrna* 2013-06-11T13:01:31.821194 node425 | - remove\_rrna | ``` Wall Time (s)      :      2781.94 [sum] User Time (s)      :     11997.03 [sum] System Time (s)    :       696.59 [sum] Memory (KB)        :       62,872 [max] Virtual Memory (KB):      332,388 [max] ``` |
| **Run 52** *assemble* 2013-06-19T10:21:44.894719 node533 | - assemble | ``` Wall Time (s)      :     41912.56 [sum] User Time (s)      :    184092.80 [sum] System Time (s)    :      9083.76 [sum] Memory (KB)        :       46,824 [max] Virtual Memory (KB):      313,084 [max] ``` |
| **Run 56** *postassemble* 2013-06-19T22:00:26.832656 node533 | - postassemble | ``` Wall Time (s)      :     12420.06 [sum] User Time (s)      :    181331.60 [sum] System Time (s)    :       472.01 [sum] Memory (KB)        :      378,032 [max] Virtual Memory (KB):      648,632 [max] ``` |
| **Run 58** *load* 2013-06-20T01:27:30.928107 node533 |  | ``` Wall Time (s)      :       154.83 [sum] User Time (s)      :         6.27 [sum] System Time (s)    :         1.69 [sum] Memory (KB)        :      148,268 [max] Virtual Memory (KB):      414,680 [max] ``` |

## sanitize (Run 10)

> Filters raw paired-end Illumina data to remove very low quality read pairs,
> read pairs with adapter sequences, and read pairs with highly skewed base
> composition. It then randomizes the order of reads in the files (applying
> the same order of randomization to each file in the pair) to make it simple
> to get random subsets of read pairs in later analyses. Finally, fastqc is
> run to profile the quality of the reads.

#### Illumina Filtering

|  |  |
| --- | --- |
| **Read pairs examined** | 38,233,199 |
| **Read pairs kept** | 31,086,355 |
| **Percent kept** | 81.3% |
| **Illumina quality threshold** | 28 |
| **Adapter fails** | 91,856 |
| **Quality fails** | 7,681,925 |
| **Base composition fails** | 2,396,996 |


#### FastQC reports

|  |  |
| --- | --- |
| 10.fastqc.1  ``` PASS Basic Statistics  PASS Per base sequence quality  PASS Per sequence quality scores  FAIL Per base sequence content  FAIL Per base GC content  PASS Per sequence GC content  WARN Per base N content  PASS Sequence Length Distribution  PASS Sequence Duplication Levels  WARN Overrepresented sequences  WARN Kmer Content ``` | 10.fastqc.2  ``` PASS Basic Statistics  PASS Per base sequence quality  PASS Per sequence quality scores  FAIL Per base sequence content  FAIL Per base GC content  PASS Per sequence GC content  PASS Per base N content  PASS Sequence Length Distribution  PASS Sequence Duplication Levels  WARN Overrepresented sequences  WARN Kmer Content ``` |

> FastQC is a tool from Babraham Bioinformatics that generates detailed
> quality diagnostics of NGS sequence data.

#### Resourse Usage

| Wall Time (s) | User Time (s) | System Time (s) | Memory (KB) | Virtual Memory (KB) |
| --- | --- | --- | --- | --- |
 1264.59 [sum] | 416.08 [sum] | 733.51 [sum] | 15,618,212 [max] | 16,120,260 [max] |

 Show/hide details

| Command | Stage | Wall Time (s) | User Time (s) | System Time (s) | Memory (KB) | Virtual Memory (KB) |
| --- | --- | --- | --- | --- | --- | --- |
| randomize | randomize | 171.46 | 47.78 | 17.56 | 15,618,212 | 16,120,260 |
| fastqc | fastqc | 3.12 | 3.62 | 0.20 | 349,544 | 6,134,776 |
| filter\_illumina | sanitize | 1090.01 | 364.68 | 715.74 | 1,340 | 16,100 |

 Back to TOC

## insert\_size (Run 12)

> Estimates the insert size distribution of paired-end Illumina data by assembling a subset
> of the data and mapping read pairs to it. The insert size does not include the adapters
> added during library preparation.

|  |  |
| --- | --- |
| **Mean insert size (bp)** | 262.23 |
| **Standard deviation (bp)** | 49.78 |

> A histogram of insert sizes.

#### Resourse Usage

| Wall Time (s) | User Time (s) | System Time (s) | Memory (KB) | Virtual Memory (KB) |
| --- | --- | --- | --- | --- |
 29.09 [sum] | 90.01 [sum] | 6.11 [sum] | 611,156 [max] | 2,137,452 [max] |

 Show/hide details

| Command | Stage | Wall Time (s) | User Time (s) | System Time (s) | Memory (KB) | Virtual Memory (KB) |
| --- | --- | --- | --- | --- | --- | --- |
| filter\_illumina | subset | 4.27 | 1.22 | 2.33 | 1,292 | 16,100 |
| velveth | subset\_oases.oases\_assemblies.k31 | 1.28 | 6.84 | 0.38 | 611,156 | 2,137,452 |
| velvetg | subset\_oases.oases\_assemblies.k31 | 4.50 | 11.56 | 0.16 | 178,416 | 1,387,128 |
| oases | subset\_oases.oases\_assemblies.k31 | 0.76 | 0.70 | 0.04 | 60,368 | 77,856 |
| velveth | subset\_oases.oases\_assemblies.k41 | 1.24 | 6.72 | 0.33 | 550,956 | 1,613,164 |
| velvetg | subset\_oases.oases\_assemblies.k41 | 2.69 | 7.25 | 0.09 | 144,364 | 1,647,312 |
| oases | subset\_oases.oases\_assemblies.k41 | 0.53 | 0.48 | 0.04 | 51,180 | 68,644 |
| velveth | subset\_oases.oases\_assemblies.k51 | 1.16 | 5.57 | 0.32 | 531,028 | 1,744,236 |
| velvetg | subset\_oases.oases\_assemblies.k51 | 1.70 | 4.69 | 0.07 | 110,384 | 1,646,064 |
| oases | subset\_oases.oases\_assemblies.k51 | 0.42 | 0.37 | 0.03 | 46,596 | 64,064 |
| velveth | subset\_oases.oases\_assemblies.k61 | 1.09 | 4.09 | 0.27 | 482,148 | 2,137,452 |
| velvetg | subset\_oases.oases\_assemblies.k61 | 1.40 | 4.21 | 0.06 | 112,148 | 1,263,608 |
| oases | subset\_oases.oases\_assemblies.k61 | 0.36 | 0.32 | 0.03 | 45,124 | 62,548 |
| filter\_illumina | stats\_subset | 0.53 | 0.10 | 0.22 | 1,288 | 16,100 |
| bowtie2-build | bowtie | 1.76 | 1.69 | 0.06 | 101,660 | 177,012 |
| bowtie2 | bowtie | 3.90 | 32.79 | 1.61 | 361,048 | 1,215,956 |
| samtools | bowtie\_to\_bam.bam | 0.51 | 0.49 | 0.02 | 3,968 | 20,764 |
| samtools | bowtie\_to\_bam.sort | 0.86 | 0.81 | 0.04 | 141,316 | 161,968 |
| samtools | bowtie\_to\_bam.index | 0.07 | 0.06 | 0.00 | 1,436 | 19,112 |
| insert\_stats | estimate\_insert | 0.07 | 0.05 | 0.01 | 1,292 | 16,072 |

 Back to TOC

## remove\_rrna (Run 13)

> Assembles and identifies ribosomal RNA (rRNA) sequences, removes read pairs
> that map to these rRNA sequences, and provides a variety of diagnostics about
> rRNA. A single exemplar sequence is presented for each type of rRNA that is
> found, but rRNA read pairs are excluded by mapping to a large set of rRNA
> transcripts that are derived from multiple assemblies over a range of data
> subset sizes.

|  |  |
| --- | --- |
| **Read pairs examined** | 31,086,355 |
| **Read pairs kept** | 24,690,259 |
| **Percent kept** | 79.4% |

#### large-mito-rRNA / 1 target(s) / 20,655 pairs removed (0.07%)

**Locus\_500000.1230\_Transcript\_1/1\_Confidence\_1.000\_Length\_926:**

|  |  |
| --- | --- |
| **Mean coverage** | 1376.7 |
| **Median coverage** | 1,089 |
| **Min coverage** | 54 |
| **Max coverage** | 3,665 |

  
  

```
  >large-mito-rRNA|Locus_500000.1230_Transcript_1/1_Confidence_1.000_Length_926|Run13|SRX288432
  ATTTTATAGAAAGTAATAATATAAATATGATTAAAATTAAATTAATTTCTGTAGTTTCTTATATTTTACTAGAATATTAT
  TAAGGTAATTCAATTTCTCATTTATAAAGGAAATAAATAATAAAACTTATAAAAAAATTATATTTATAAATATAATTTTT
  TGTACTAATCTAAATCAAGTAGAATATAATAATATAAATAAATTTATTTAAGGAACTCGGCAAAATAAAATATCGACTGT
  TTACCAAAAACATAGCTATAAATAAAATTTGAAGTGCAACCTGCCCAGTGGTTTTTTTAATATATTATATTAAATTATAA
  TAAAAAAAAAATTAAACGGCCGCGTTATTCCTGATCGTGAAAATGTAGCATAATAATTTGTCATTTAATTAGTGGAGAGT
  ATGAACGGTTTAACGAATATTTCACTGTCTTAAATAAATATTATATTAAATTTGAATAATAGTTAAGATACTATTTAATT
  TTGTAAGACGAAAAGACCCTATAGAGCTTTACTAATATTAAATATAAAAAAATGTTTTTTTATTGTTTGAGAATTTAGTT
  TAGTTGGGGAGACTGCCTTTTATTTTAAACGAAGGTATACAAAAATATTTATTGTATAATTTATAAATTTAACAATTATA
  AAAATAGGTATAAAATGACCCATTTTAAATATAAAAATATTTAAATGATTTATAAATAATACGCTACCTTAGGGATAACA
  GAATAATATTAATTTTGAGACCATATTGAATTTAATGTTTATTACCTCTATGTTGAATTAAGATATCCTAATGATTTGTA
  GCAGTCATTAAAGGTAGGTCTGTTCGACCTTTAAAATCTTACATGATTTGAGTTCATTCCGTTGTAAAACAGGAAGGTTT
  CTATCTACAAAACCCACATTTAAAAATAATTAGTACGAAAGGAATT
```

Top NCBI nt hit: gnl|BL\_ORD\_ID|160255 gi|161338541|emb|AM887994.1| Cryptolaria pectinata mitochondrial partial 16S rRNA gene (E-value: 3.45155e-89)

#### large-nuclear-rRNA / 1 target(s) / 1,080,400 pairs removed (3.48%)

**Locus\_100000.20\_Transcript\_1/1\_Confidence\_1.000\_Length\_3609:**

|  |  |
| --- | --- |
| **Mean coverage** | 11640.2 |
| **Median coverage** | 9,985 |
| **Min coverage** | 4 |
| **Max coverage** | 44,146 |

  
  

```
  >large-nuclear-rRNA|Locus_100000.20_Transcript_1/1_Confidence_1.000_Length_3609|Run13|SRX288432
  GATCAGACAAGACTACCCGCTGAATTTAAGCATATTAATAAGCGGAGGAAAAGAAACTAACAAGGATTCCCCTAGTAACG
  GCGAGTGAAGCGGGATCAGCTCAAACTTAAAATCTGCGTTGCTTGCAACGCCGAATTGTAGTCTAGAGATTCGTTTTCAA
  GGCGAATGCGCAGTACTTAAGTTGCTTGGAACGGCACATCGTAGAGGGTGACAATCCCGTACGTGGTACTGTGCATCGTT
  CACGATGCGTTTTCTATGAGTCGGGTTGCTTGGTAATGCAGCCCAAAATTGGAGGTAAACTCCTTCTAAAGCTAAATATT
  GGCACGAGACCGATAGCGAACAAGTACCGTGAGGGAAAGATGAAAAGCACTTTGAAAAGAAAGTTAATAGTACGTGAAAC
  CGTTAGGAGGGAAGCGCATGGAATTAGCAATGCACTGTCGAGATTCAGACGATCGGTGTTGAGTACGGACGTCGTACGGA
  TCCGAATGGACCGTTGGCGTTCGTCACTTAGTTCTGGTTGTCGCATTTCCCGGCGGTGTGCGTCAACAGGTATTGGAACC
  GGGTGATACGCCTCGCAAGAAGGTGGCTGGCTTCGGTCAGTGTTATAGCTTGTGGTGTGCTAGCTCGGATTCGATAGAGG
  TGTCGCAGCACATGCCCTCACGGGCTGGCTTCTGTTTCCTCGGTCTGGTGTGACCATAGCGGACTGCATGCAGTGCGTTT
  GAACTTCATCCGGCTGTCGGAGGCAAGAATGCACACTATGTGCTTAGGTTGTTGGCGGTCATATGGTTTCATGCGACCCG
  TCTTGTAACACGGACCAAGGAGTCTAACATGTGTGCGAGTCTTAGGGTGATTGAAACCCGCAGGCACAATGAAAGTAAAG
  GCTCTTCTGAGCTGAGGTGAGATCTCTTTGGCTTCGGTTGAAGAGCGCATCATCGACCGACCTATTCTAATCCTAGAAAG
  GTTTGAGTAAGAGCACATCTGTTGGGACCCGAAAGATGGTGAACTATGCTTGAGTAGGGCGAAGCCAGAGGAAACTCTGG
  TGGAGGCTCGTAGCGATTCTGACGTGCAAATCGATCGTCAAACTTGAGTATAGGGGCGAAAGACTAATCGAACCATCTAG
  TAGCTGGTTCCCTCCGAAGTTTCCCTTAGGATAGCTGGAACTCGGAACAGTTTTATCAGGTAAAGCGAATGATTAGAGGT
  CTTAGGGTTGAAACAACCTTAACCTATTCTCAAACTTTAAATTGGTAAGAAGCCCGACTTGCTCGACTGAAGTAGGGCAC
  AGAATGAGAGTTCTTAGTGGGCCATTTTTGGTAAGCAGAACTGGCGATGCGGGATGAACCGAACGCTGAGTTAAGGCGCC
  TAAATCGACGCTCATCAGACCCCACAAAAGGTGTTGGTTGATCCAGACAGCAGGACGGTGGCCATGGAAGTCGGAATCCG
  CTAAGGAGTGTGTAACAACACACCTGCCGAATCAACTAGCCCTGAAAATGGATGGCGCTCAAGCGTCGTGCCTATACTCA
  GCCGTCAGAGTAAATAGCGAAGCTCTGACGAGTAGGAGGGCGTGGGGGTCGTGACGCAGCCTTTGGCGTGAGCCTGGGTG
  AAACGGCCTCTAGTGAAGATCTTGGTGGTAGTAGCAAATATTCAAATGAGAACTTTGAAGACCGAAGTGGAGAAAGGTTC
  CATGTGAACAGCAGTTGGACATGGGTTAGTCGATCCTAAGAGATAGGGAAATTCCGTTTCAAAGTGTCCAATCTTGGACC
  ATCGATCGAAAGGGAATCGGGTTAAAATTCCCGAACCAGAACGTGGATATTCTACCCCTCCGGGGTTTAGATGTGCGGTA
  ACGCAACTGAACTCGGAAACGTCGGCAGGAGCCCTGGGAAGAGTTCTCTTTTCTTGTTAACGGCCTGACACCATGGAATC
  TGATTGCCAGGAGATATGGTTTGATGGCCGGTAAAGCACCACACTTCATGTGGTGTCCGGTGCGCTCCTGAAGGCCCTTG
  AAAATCCGAGGGAAAGAGTGATTTTCACGTCTGTTCGTACTCATAACCGCAGCAGGTCTCCAAGGTGAGCAGCCTCTGGT
  CGATAGAACAATGTAGGTAAGGGAAGTCGGCAAAATAGATCCGTAACTTCGGGAAAAGGATTGGCTCTAAGGGTTGGGTC
  TGTCGGGCTGAGACTTGAAGCGAGTGGATCCAACCTGGACTGGCTTTGGCCTCTCGGGGCTATGGTCGGACTGGGAAGGA
  ACTAGTCGTGGATTGGCCCAGCTATGCTCGCAAGGGCAGTTCGGCAGGCAATTAACAATCAACTTAGAACTGGTACGGAC
  AAGGGGAATCCGACTGTTTAATTAAAACAAAGCATTGCGATGGCCGGAAACGGTGTTGACGCAATGTGATTTCTGCCCAG
  TGCTCTGAATGTCAAAGTGAAGAAATTCAACCAAGCGCGGGTAAACGGCGGGAGTAACTATGACTCTCTTAAGGTAGCCA
  AATGCCTCGTCATCTAATTAGTGACGCGCATGAATGGATTAACGAGATTCCCACTGTCCCTATCTACTATCTAGCGAAAC
  CACAGCCAAGGGAACGGGCTTGGCAAAATCAGCGGGGAAAGAAGACCCTGTTGAGCTTGACTCTAGTCTGACTTTGTGAA
  AAGACATAGGAGGTGTAGAATAGGTGGGAGCAGTAATGCAAAAGTGAAATACCACTACTCTTATAGTTTTTTTACTTATT
  CGATTGAGCGGAAGCGAGCTTCACGGCTCATTTTCTAGAATTAAGGCCCCGTTGGCGGGTCGATCCGTGTCGAAGACACT
  GTCAGGTTGGGAGTTTGGCTGGGGCGGCACATCTGTCAAATGATAACGCAGGTGTCCTAAGGTGAGCTCAATGAGAACGG
  AAATCTCATGTAGAACAAAAGGGTAAAAGCTCACTTGATTTTGATTTTCAGTATGAATACAAACCGTGAAAGCGTGGCCT
  ATCGATCCTTTAGTCTTTAGGAGTTTTAAGCTAGAGGTGTCAGAAAAGTTACCACAGGGATAACTGGCTTGTGGCAGCCA
  AGCGTTCATAGCGACGTTGCTTTTTGATCCTTCGATGTCGGCTCTTCCTATCATTGCGAAGCAGAATTCGCCAAGTGTTG
  GATTGTTCACCCACTAATAGGGAACGTGAGCTGGGTTTAGACCGTCGTGAGACAGGTTAGTTTTACCCTACTGATGAAGT
  GTTGTTGCAATAGTAATTCTGCTCAGTACGAGAGGAACCGCAGATTCAGACAATTGGCATTTGCACTTGCTTGAAAAGGC
  AATGGTGCGAAGCTACCATCTGTTGGATTATGACTGAACGCCTCTAAGTCAGAATCCGTGCTAGAAAGCAATGATAATTA
  CCTCTGGATAATCTTAGGCGAATAAGAATAGAACAGCTTCTGTTGTTCCTGAATCTCATTGCACTGACCTAGAGAAAAAC
  TGGTGAGGTGCTGCAACTATCAAAATCTAAAATTTTCAGAGATAAATCCTATGCAGACGACTTAAACAAGAACGTGGTAT
  TGTAAAAAGCAGAGTAGCCTCTGTGCTACGATCTTCTGAGATTAAGCCTCTGTTCGTAGATTTGTAAAAAAACAAAAAGA
  ATAAAGAAG
```

Top NCBI nt hit: gnl|BL\_ORD\_ID|22504 gi|9187620|emb|AJ271027.1| Sequoiadendron giganteum partial 25S rRNA gene (E-value: 0.0)

#### small-mito-rRNA / no targets

#### small-nuclear-rRNA / 1 target(s) / 5,295,041 pairs removed (17.03%)

**Locus\_250000.1\_Transcript\_5/8\_Confidence\_0.222\_Length\_2461:**

|  |  |
| --- | --- |
| **Mean coverage** | 25396.4 |
| **Median coverage** | 9,126 |
| **Min coverage** | 117 |
| **Max coverage** | 113,110 |

  
  

```
  >small-nuclear-rRNA|Locus_250000.1_Transcript_5/8_Confidence_0.222_Length_2461|Run13|SRX288432
  AAGTAATGATTAAGAGGGACAATTGGGGGCATCCGTATTTCGTTGTCAGAGGTGAAATTCTTGGATTTACGAAAGACGAA
  CAACTGCGAAAGCACTTGCCAAGAGTGTTTTCATTAATCAAGAACGAAAGTTAGAGGATCGAAGACGATCAGATACCGTC
  CTAGTTCTAACCATAAACGATGTCGACTAGGGATCAGCGGGCGTTATCGTACGACCCCGTTGGCACCTTACGGGAAACCA
  AAGTTTCTGGATTCCGGGGGAAGTATAGTCGCAAGGCCGAAACTTAAAGGAATTGACGGAAGGGCACCACCAGGAGTGGA
  GCCTGTGGCTCAATTTGACTCAACACGGGAAAACTTACCAGGTCCAGACATAGTAAGGATTGACAGGTTGAGAGCCCTTT
  CTTGATTCTATGGGTGGTGGTGCATGGCCGTTCTTAGTTGGTGGAGTGATTTGTCTGGTTAATTCCGTTAACGAACGAGA
  CCTTAACCGGCTAAATAGTCATGCGATTCTCGAATCGTAACTGACTTCTTAGAGGGACTGTTGGTGTTTAACCAAAGTCA
  GGAAGGCAATAACAGGTCTGTGATGCCCTTAGATGTTCTGGGCCGCACGCGCGCTACACTGTCGGATTCAGCGAGTCTTA
  ACCTTAACCGAAAGGTTTGGGTAATCTTTTGAAAGTCCGACGTGATGGGGATTGATCATTGCAATTATTGATCATGAACG
  AGGAATTCCTAGTAAATGCGAGTCATCAGCTCGCGTTGATTACGTCCCTGCCCTTTGTACACACCGCCCGTCGCTACTAC
  CGATTGAATGGTTTAGTGAGATCTTCGGATTGGCACCGTCGCGGCCTCACGGAAGTGATGGTGCCGAAAAGTTGCTCAAA
  CTTGATCATTTAGAGGAAGTAAAAGTCGTAACAAGGTTTCCGTAGGTGAACCTGCGGAAGGATCATTACCGTTTGTCTTT
  TGACAACACCACTGTGAACTGTACCAAGCAGGTGGGGTAACGCTAATTGTGTTTTAAATGATCTCGATCGGCCCCACTTG
  TGCAACCATTTGTGTATTTTTACGCACTTGTTTCATCATGTATGCTTTGCTCTCTTGAGCTAAGCAAATCAAAAACGTGA
  CAACTTCTAACGGTGGATCTCTTGGCTCGTGCATCGATGAAGAACGTAGCCAGTTGCGATAAGTAGTGTGAATTGCAGAA
  TTCAGTGAATCATCGAATCTTTGAACGCAAATTGCGCTTTCTGGATATCCAGGGAGCATGCCTGTCTGAGCGTCGTTTAA
  TAATCTACACACCTCGGTGTGGGTAGGGAGGTCATGCGACTCTTCATTGAGACGCCTGTCTCTCGATGTTTCTGAACCTA
  GCGGTGTGCACTGTGTGTACTGCTTGGAAGGCTCAACTTGCTTCTGTCCTTCTTCAGCAGTGCATGTGATAACGCACTAG
  TGTTCAACTTCGGCATTAGCTGAAATCTTAATTTTGACCTCAGATCAGACAAGACTACCCGCTGAATTTAAGCATATTAA
  TAAGCGGAGGAAAAGAAACTAACAAGGATTCCCCTAGTAACGGCGAGTGAAGCGGGATCAGCTCAAACTTAAAATCTGCG
  TTGCTTGCAACGCCGAATTGTAGTCTAGAGATTCGTTTTCAAGGCGAATGCGCAGTACTTAAGTTGCTTGGAACGGCACA
  TCGTAGAGGGTGACAATCCCGTACGTGGTACTGTGCATCGTTCACGATGCGTTTTCTATGAGTCGGGTTGCTTGGTAATG
  CAGCCCAAAATTGGAGGTAAACTCCTTCTAAAGCTAAATATTGGCACGAGACCGATAGCGAACAAGTACCGTGAGGGAAA
  GATGAAAAGCACTTTGAAAAGAAAGTTAATAGTACGTGAAACCGTTAGGAGGGAAGCGCATGGAATTAGCAATGCACTGT
  CGAGATTCAGACGATCGGTGTTGAGTACGGACGTCGTACGGATCCGAATGGACCGTTGGCGTTCGTCACTTAGTTCTGGT
  TGTCGCATTTCCCGGCGGTGTGCGTCAACAGGTATTGGAACCGGGTGATACGCCTCGCAAGAAGGTGGCTGGCTTCGGTC
  AGTGTTATAGCTTGTGGTGTGCTAGCTCGGATTCGATAGAGGTGTCGCAGCACATGCCCTCACGGGCTGGCTTCTGTTTC
  CTCGGTCTGGTGTGACCATAGCGGACTGCATGCAGTGCGTTTGAACTTCATCCGGCTGTCGGAGGCAAGAATGCACACTA
  TGTGCTTAGGTTGTTGGCGGTCATATGGTTTCATGCGACCCGTCTTGTAACACGGACCAAGGAGTCTAACATGTGTGCGA
  GTCTTAGGGTGATTGAAACCCGCAGGCACAATGAAAGTAAAGGCTCTTCTGAGCTGAGGTGAGATCTCTTTGGCTTCGGT
  TGAAGAGCGCATCATCGACCGACCTATTCTAATCCTAGAAAGGTTTGAGTAAGAGCACATC
```

Top NCBI nt hit: gnl|BL\_ORD\_ID|17783 gi|5931695|emb|AJ133549.1| Bellonella unicolor 18S rRNA gene (E-value: 0.0)

#### Resourse Usage

| Wall Time (s) | User Time (s) | System Time (s) | Memory (KB) | Virtual Memory (KB) |
| --- | --- | --- | --- | --- |
 2772.23 [sum] | 11991.90 [sum] | 695.94 [sum] | 3,208,620 [max] | 3,223,424 [max] |

 Show/hide details

| Command | Stage | Wall Time (s) | User Time (s) | System Time (s) | Memory (KB) | Virtual Memory (KB) |
| --- | --- | --- | --- | --- | --- | --- |
| filter\_illumina | subset\_assemblies.500 | 0.02 | 0.01 | 0.01 | 1,292 | 16,100 |
| velveth | subset\_assemblies.500.oases\_assemblies.k61 | 0.05 | 0.35 | 0.07 | 203,220 | 1,965,680 |
| velvetg | subset\_assemblies.500.oases\_assemblies.k61 | 0.05 | 0.18 | 0.02 | 67,076 | 1,679,584 |
| oases | subset\_assemblies.500.oases\_assemblies.k61 | 0.02 | 0.00 | 0.00 | 1,312 | 35,980 |
| filter\_illumina | subset\_assemblies.1000 | 0.04 | 0.01 | 0.02 | 1,296 | 16,100 |
| velveth | subset\_assemblies.1000.oases\_assemblies.k61 | 0.04 | 0.18 | 0.07 | 203,248 | 1,834,876 |
| velvetg | subset\_assemblies.1000.oases\_assemblies.k61 | 0.05 | 0.21 | 0.02 | 67,276 | 1,548,796 |
| oases | subset\_assemblies.1000.oases\_assemblies.k61 | 0.01 | 0.00 | 0.00 | 1,560 | 36,272 |
| filter\_illumina | subset\_assemblies.2500 | 0.09 | 0.03 | 0.06 | 1,296 | 16,100 |
| velveth | subset\_assemblies.2500.oases\_assemblies.k61 | 0.06 | 0.21 | 0.06 | 205,920 | 1,507,224 |
| velvetg | subset\_assemblies.2500.oases\_assemblies.k61 | 0.08 | 0.45 | 0.02 | 67,892 | 1,680,484 |
| oases | subset\_assemblies.2500.oases\_assemblies.k61 | 0.02 | 0.01 | 0.00 | 2,524 | 36,772 |
| filter\_illumina | subset\_assemblies.5000 | 1.02 | 0.06 | 0.12 | 1,292 | 16,100 |
| velveth | subset\_assemblies.5000.oases\_assemblies.k61 | 0.09 | 0.35 | 0.07 | 216,416 | 1,705,500 |
| velvetg | subset\_assemblies.5000.oases\_assemblies.k61 | 0.11 | 0.58 | 0.03 | 68,904 | 1,681,508 |
| oases | subset\_assemblies.5000.oases\_assemblies.k61 | 0.03 | 0.02 | 0.00 | 3,668 | 38,460 |
| filter\_illumina | subset\_assemblies.10000 | 0.48 | 0.11 | 0.24 | 1,292 | 16,100 |
| velveth | subset\_assemblies.10000.oases\_assemblies.k61 | 0.16 | 0.80 | 0.07 | 228,580 | 1,533,528 |
| velvetg | subset\_assemblies.10000.oases\_assemblies.k61 | 0.17 | 0.94 | 0.03 | 71,088 | 1,355,964 |
| oases | subset\_assemblies.10000.oases\_assemblies.k61 | 0.04 | 0.03 | 0.01 | 5,472 | 39,876 |
| filter\_illumina | subset\_assemblies.25000 | 0.96 | 0.28 | 0.59 | 1,296 | 16,100 |
| velveth | subset\_assemblies.25000.oases\_assemblies.k61 | 2.74 | 1.53 | 0.12 | 278,040 | 2,106,748 |
| velvetg | subset\_assemblies.25000.oases\_assemblies.k61 | 0.37 | 1.77 | 0.03 | 77,576 | 1,637,168 |
| oases | subset\_assemblies.25000.oases\_assemblies.k61 | 0.09 | 0.07 | 0.01 | 11,692 | 42,012 |
| filter\_illumina | subset\_assemblies.50000 | 1.85 | 0.61 | 1.09 | 1,296 | 16,100 |
| velveth | subset\_assemblies.50000.oases\_assemblies.k61 | 0.58 | 2.62 | 0.19 | 348,920 | 1,592,204 |
| velvetg | subset\_assemblies.50000.oases\_assemblies.k61 | 0.70 | 2.19 | 0.07 | 88,360 | 1,639,564 |
| oases | subset\_assemblies.50000.oases\_assemblies.k61 | 0.18 | 0.14 | 0.02 | 22,648 | 44,396 |
| filter\_illumina | subset\_assemblies.100000 | 3.67 | 1.21 | 2.33 | 1,296 | 16,100 |
| velveth | subset\_assemblies.100000.oases\_assemblies.k61 | 1.12 | 4.77 | 0.30 | 482,144 | 1,613,164 |
| velvetg | subset\_assemblies.100000.oases\_assemblies.k61 | 1.41 | 4.18 | 0.07 | 110,140 | 1,263,608 |
| oases | subset\_assemblies.100000.oases\_assemblies.k61 | 0.37 | 0.31 | 0.04 | 45,116 | 62,572 |
| filter\_illumina | subset\_assemblies.250000 | 9.09 | 3.03 | 5.82 | 1,292 | 16,100 |
| velveth | subset\_assemblies.250000.oases\_assemblies.k61 | 2.74 | 11.78 | 0.60 | 844,796 | 1,675,780 |
| velvetg | subset\_assemblies.250000.oases\_assemblies.k61 | 3.94 | 10.45 | 0.13 | 175,612 | 1,532,788 |
| oases | subset\_assemblies.250000.oases\_assemblies.k61 | 1.00 | 0.88 | 0.10 | 114,148 | 131,668 |
| filter\_illumina | subset\_assemblies.500000 | 17.97 | 5.95 | 11.45 | 1,292 | 16,100 |
| velveth | subset\_assemblies.500000.oases\_assemblies.k61 | 5.22 | 21.98 | 1.08 | 1,384,972 | 2,304,540 |
| velvetg | subset\_assemblies.500000.oases\_assemblies.k61 | 9.08 | 25.91 | 0.22 | 262,552 | 1,372,392 |
| oases | subset\_assemblies.500000.oases\_assemblies.k61 | 2.16 | 1.99 | 0.16 | 231,888 | 249,408 |
| filter\_illumina | subset\_assemblies.1000000 | 35.23 | 12.07 | 22.24 | 1,292 | 16,100 |
| velveth | subset\_assemblies.1000000.oases\_assemblies.k61 | 10.29 | 45.15 | 1.79 | 1,998,500 | 2,713,996 |
| velvetg | subset\_assemblies.1000000.oases\_assemblies.k61 | 21.91 | 66.39 | 0.43 | 441,916 | 1,523,780 |
| oases | subset\_assemblies.1000000.oases\_assemblies.k61 | 7.46 | 4.62 | 0.33 | 478,008 | 495,528 |
| makeblastdb | blast\_rrna | 0.03 | 0.00 | 0.01 | 8,172 | 44,988 |
| blastn | blast\_rrna | 6.09 | 5.82 | 0.29 | 175,600 | 1,980,684 |
| blastn | blast\_nt | 1.28 | 1.27 | 0.18 | 131,488 | 2,092,444 |
| bowtie2-build | bowtie | 0.82 | 0.26 | 0.05 | 98,636 | 175,944 |
| bowtie2 | bowtie | 693.73 | 10951.61 | 79.32 | 311,136 | 1,173,596 |
| samtools | bowtie\_to\_bam.bam | 208.52 | 203.66 | 3.83 | 3,404 | 20,108 |
| samtools | bowtie\_to\_bam.sort | 224.86 | 221.00 | 1.97 | 629,692 | 752,544 |
| samtools | bowtie\_to\_bam.index | 11.23 | 10.95 | 0.25 | 940 | 18,744 |
| samtools | bam\_pileup | 243.26 | 207.25 | 1.67 | 107,296 | 124,200 |
| samtools | bam\_extract\_ids.large-nuclear-rRNA | 21.34 | 4.96 | 0.38 | 2,076 | 18,748 |
| samtools | bam\_extract\_ids.small-nuclear-rRNA | 75.37 | 22.52 | 1.51 | 2,080 | 18,748 |
| samtools | bam\_extract\_ids.large-mito-rRNA | 0.19 | 0.10 | 0.01 | 2,068 | 18,748 |
| exclude | exclude\_ids | 1142.75 | 128.08 | 556.30 | 3,208,620 | 3,223,424 |

 Back to TOC

## assemble (Run 52)

> Assembles reads into transcripts, processes the assembly, and generates
> assembly diagnostics. Read pairs are first filtered at a more stringent
> mean quality threshold. Assemblies are then performed over a range of
> data subset sizes, which provides an indication of how sequencing effort
> impacts assembly results.

#### Illumina Filtering

|  |  |
| --- | --- |
| **Read pairs examined** | 24,690,259 |
| **Read pairs kept** | 20,235,317 |
| **Percent kept** | 82.0% |
| **Illumina quality threshold** | 33 |
| **Adapter fails** | 0 |
| **Quality fails** | 6,089,251 |
| **Base composition fails** | 0 |

#### Resourse Usage

| Wall Time (s) | User Time (s) | System Time (s) | Memory (KB) | Virtual Memory (KB) |
| --- | --- | --- | --- | --- |
 34025.90 [sum] | 184062.75 [sum] | 9063.42 [sum] | 39,331,468 [max] | 40,949,144 [max] |

 Show/hide details

| Command | Stage | Wall Time (s) | User Time (s) | System Time (s) | Memory (KB) | Virtual Memory (KB) |
| --- | --- | --- | --- | --- | --- | --- |
| filter\_illumina | quality\_filter | 551.15 | 81.54 | 459.11 | 1,336 | 16,100 |
| trinity | trinity.20235317 | 25563.31 | 90180.22 | 4319.63 | 39,331,468 | 40,949,144 |
| parallel | trinity.20235317 | 7911.44 | 93800.99 | 4284.67 | 1,363,004 | 5,237,244 |

 Back to TOC

## postassemble (Run 56)

> Cleans transcripts to remove any rRNA or vector sequences, then selects a
> single exemplar transcript for each gene. Vector sequences could include
> untrimmed adapters or plasmids (we sometimes find sequences in our data for the
> protein expression vectors used to manufacture the sample preparation enzymes).
> Raw reads are mapped back to the exemplars to estimate coverage and assign RPKM
> values. Finally, transcripts are annotated with blastx hits against SwissProt.

#### Assemblies

| Method | Read pairs | Genes | Mean Length (bp) | N50 Length (bp) | Links to assembly files |
| --- | --- | --- | --- | --- | --- |
| trinity | 20,235,317 | 69527 | 718.589152416 | 833 | [annotated transcripts], [rrna], [vectors] |

##### trinity

|  |  |
| --- | --- |
| **Read pairs** | 20,235,317 |
| **Pairs mapped** | 52.8% |
| **Pairs discordant** | 3.0% |
| **Unpaired reads mapped** | 63.4% |

Number of exemplar transcripts in full assembly with blastx hits: 13,886


#### Resourse Usage

| Wall Time (s) | User Time (s) | System Time (s) | Memory (KB) | Virtual Memory (KB) |
| --- | --- | --- | --- | --- |
 11870.91 [sum] | 180788.13 [sum] | 470.71 [sum] | 394,016 [max] | 1,248,028 [max] |

 Show/hide details

| Command | Stage | Wall Time (s) | User Time (s) | System Time (s) | Memory (KB) | Virtual Memory (KB) |
| --- | --- | --- | --- | --- | --- | --- |
| makeblastdb | clean\_rrna.assembly\_20235317\_trinity | 0.04 | 0.01 | 0.01 | 8,168 | 45,008 |
| blastn | clean\_rrna.assembly\_20235317\_trinity | 14.96 | 120.31 | 18.14 | 174,276 | 232,344 |
| blastn | clean\_univec.assembly\_20235317\_trinity | 15.81 | 128.49 | 24.36 | 170,844 | 233,992 |
| dustmasker | dustmasker.assembly\_20235317\_trinity | 21.87 | 20.55 | 1.16 | 8,268 | 43,708 |
| bowtie2-build | coverage.assembly\_20235317\_trinity | 42.41 | 41.54 | 0.61 | 209,080 | 253,956 |
| bowtie2 | coverage.assembly\_20235317\_trinity | 647.39 | 10016.66 | 97.09 | 394,016 | 1,248,028 |
| coverage | coverage.assembly\_20235317\_trinity | 89.02 | 37.13 | 4.94 | 10,900 | 26,736 |
| blastx | nr\_annotate.assembly\_20235317\_trinity | 11039.41 | 170423.45 | 324.40 | 222,076 | 362,576 |

 Back to TOC
