# Supplementary figures and images for: Agalma: an automated phylogenomics workflow (part 2 of 2)
Source: BMC Bioinformatics. 2013 Nov 19;14:330. doi: 10.1186/1471-2105-14-330 (PMC3840672; doi:10.1186/1471-2105-14-330)

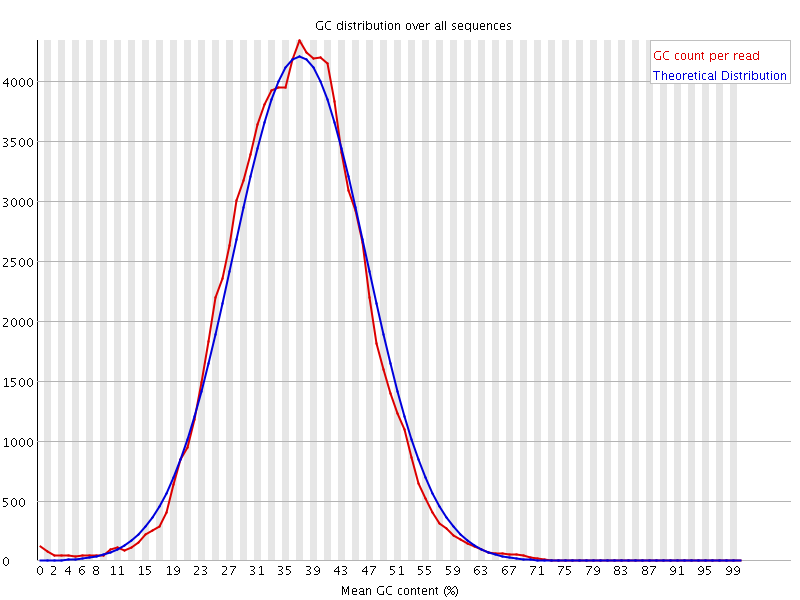

Supplement: Additional file 1 — HTML report for assembly of the sample data sets. The HTML report for the assembly of the test data sets from raw reads. The tabular report (index.html) provides an overview across the five assemblies for the ingroup taxa, and includes links (in the Catalog ID column) to detailed reports for the assembly of each species. Fasta files for the annotated transcripts have been removed from the report to reduce file size. [file 1471-2105-14-330-S1.zip › tabular/SRX288430/9.fastqc.2/Images/per_sequence_gc_content.png]

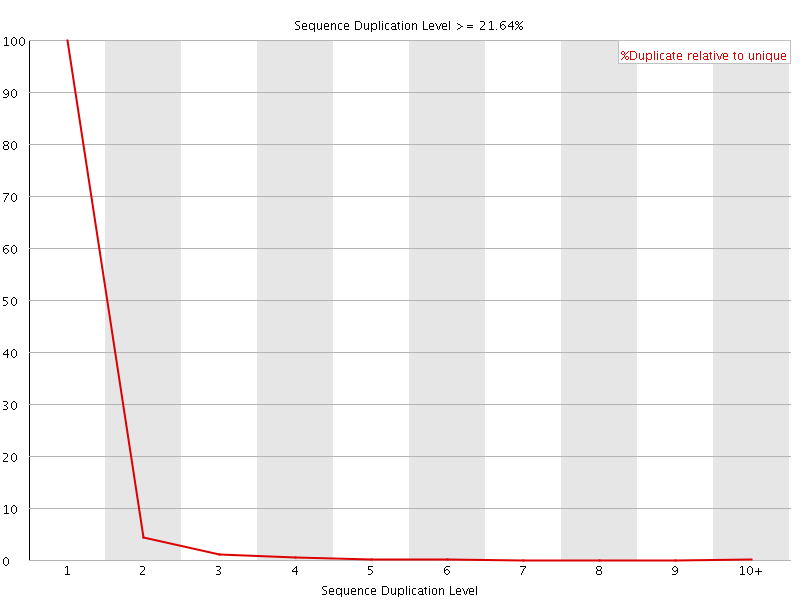

Supplement: Additional file 1 — HTML report for assembly of the sample data sets. The HTML report for the assembly of the test data sets from raw reads. The tabular report (index.html) provides an overview across the five assemblies for the ingroup taxa, and includes links (in the Catalog ID column) to detailed reports for the assembly of each species. Fasta files for the annotated transcripts have been removed from the report to reduce file size. [file 1471-2105-14-330-S1.zip › tabular/SRX288430/9.fastqc.2/Images/duplication_levels.png]

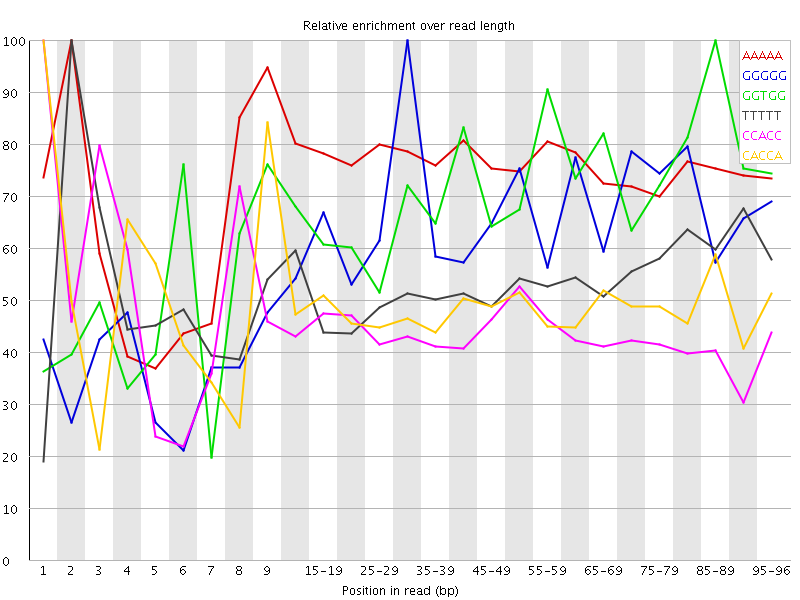

Supplement: Additional file 1 — HTML report for assembly of the sample data sets. The HTML report for the assembly of the test data sets from raw reads. The tabular report (index.html) provides an overview across the five assemblies for the ingroup taxa, and includes links (in the Catalog ID column) to detailed reports for the assembly of each species. Fasta files for the annotated transcripts have been removed from the report to reduce file size. [file 1471-2105-14-330-S1.zip › tabular/SRX288430/9.fastqc.2/Images/kmer_profiles.png]

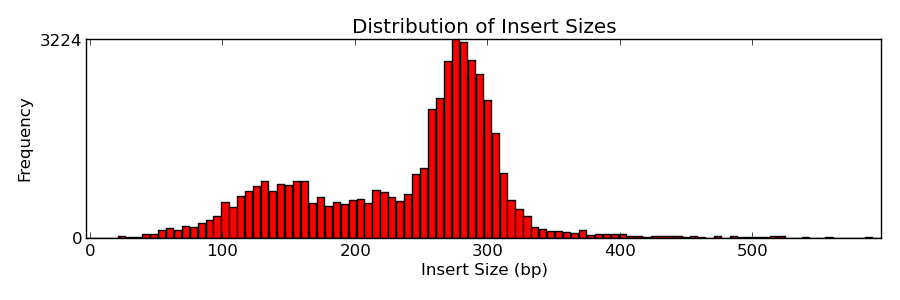

Supplement: Additional file 1 — HTML report for assembly of the sample data sets. The HTML report for the assembly of the test data sets from raw reads. The tabular report (index.html) provides an overview across the five assemblies for the ingroup taxa, and includes links (in the Catalog ID column) to detailed reports for the assembly of each species. Fasta files for the annotated transcripts have been removed from the report to reduce file size. [file 1471-2105-14-330-S1.zip › tabular/SRX288430/16.insert.hist.png]

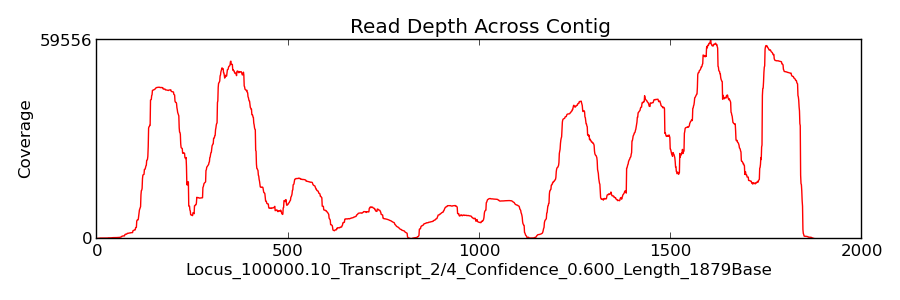

Supplement: Additional file 1 — HTML report for assembly of the sample data sets. The HTML report for the assembly of the test data sets from raw reads. The tabular report (index.html) provides an overview across the five assemblies for the ingroup taxa, and includes links (in the Catalog ID column) to detailed reports for the assembly of each species. Fasta files for the annotated transcripts have been removed from the report to reduce file size. [file 1471-2105-14-330-S1.zip › tabular/SRX288430/17.Locus_100000.10_Transcript_2_4_Confidence_0.600_Length_1879.hist.png]

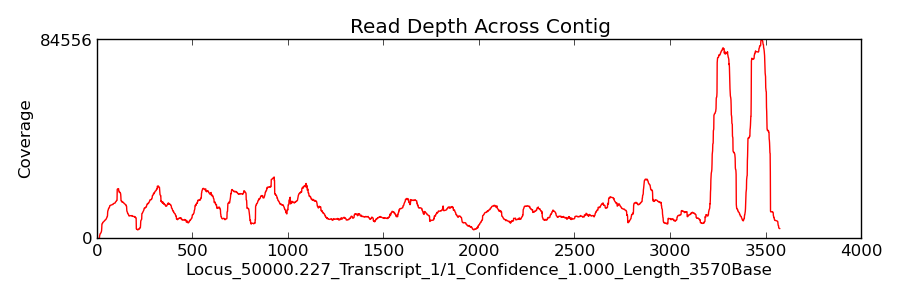

Supplement: Additional file 1 — HTML report for assembly of the sample data sets. The HTML report for the assembly of the test data sets from raw reads. The tabular report (index.html) provides an overview across the five assemblies for the ingroup taxa, and includes links (in the Catalog ID column) to detailed reports for the assembly of each species. Fasta files for the annotated transcripts have been removed from the report to reduce file size. [file 1471-2105-14-330-S1.zip › tabular/SRX288430/17.Locus_50000.227_Transcript_1_1_Confidence_1.000_Length_3570.hist.png]

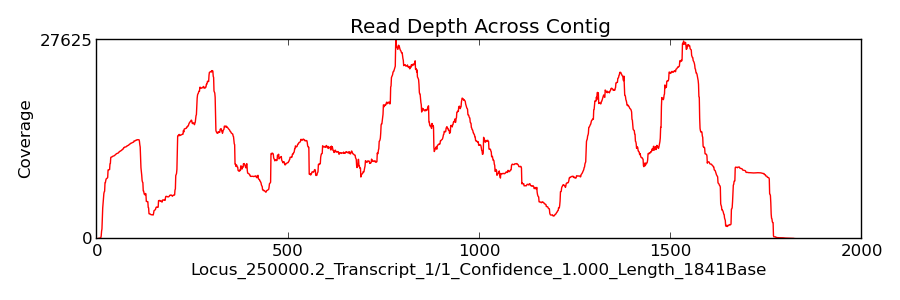

Supplement: Additional file 1 — HTML report for assembly of the sample data sets. The HTML report for the assembly of the test data sets from raw reads. The tabular report (index.html) provides an overview across the five assemblies for the ingroup taxa, and includes links (in the Catalog ID column) to detailed reports for the assembly of each species. Fasta files for the annotated transcripts have been removed from the report to reduce file size. [file 1471-2105-14-330-S1.zip › tabular/SRX288430/17.Locus_250000.2_Transcript_1_1_Confidence_1.000_Length_1841.hist.png]

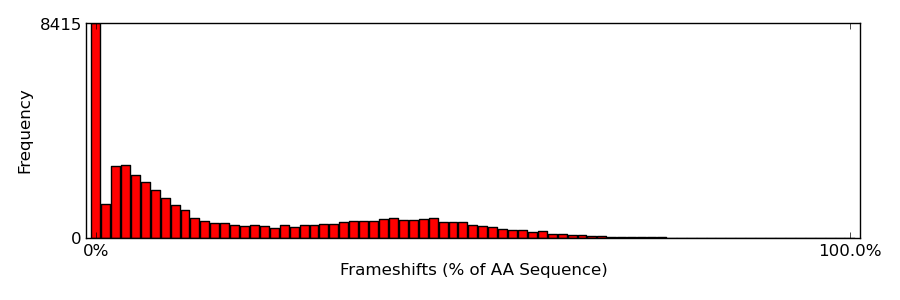

Supplement: Additional file 2 — HTML report for phylogenetic analyses. The HTML report for the phylogenetic analysis of the sample data. [file 1471-2105-14-330-S2.zip › AgalmaExampleTree/60.frameshifts.hist_sequences.png]

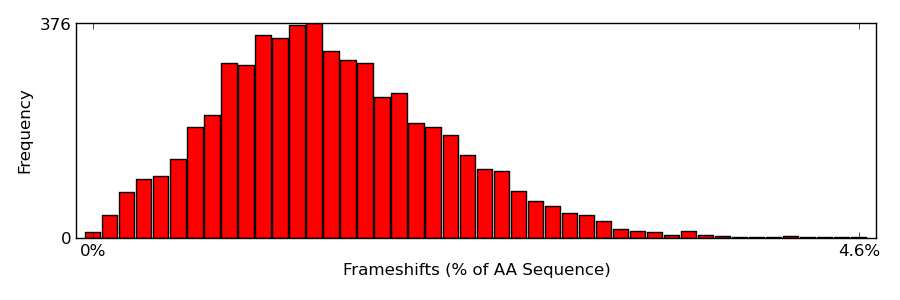

Supplement: Additional file 2 — HTML report for phylogenetic analyses. The HTML report for the phylogenetic analysis of the sample data. [file 1471-2105-14-330-S2.zip › AgalmaExampleTree/60.frameshifts.hist_clusters.png]

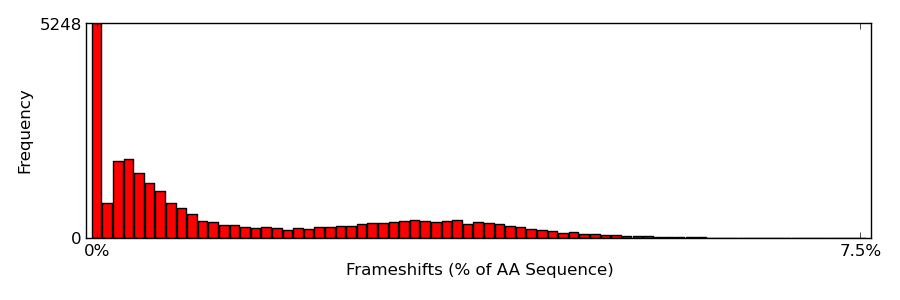

Supplement: Additional file 2 — HTML report for phylogenetic analyses. The HTML report for the phylogenetic analysis of the sample data. [file 1471-2105-14-330-S2.zip › AgalmaExampleTree/65.frameshifts.hist_sequences.png]

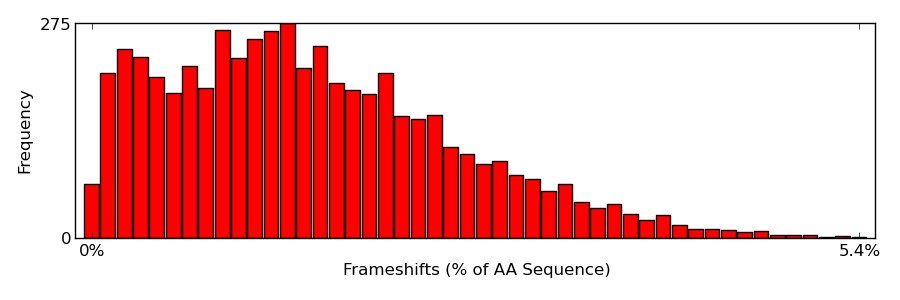

Supplement: Additional file 2 — HTML report for phylogenetic analyses. The HTML report for the phylogenetic analysis of the sample data. [file 1471-2105-14-330-S2.zip › AgalmaExampleTree/65.frameshifts.hist_clusters.png]

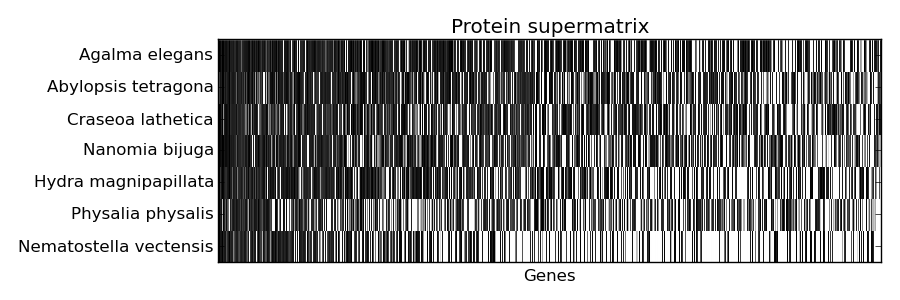

Supplement: Additional file 2 — HTML report for phylogenetic analyses. The HTML report for the phylogenetic analysis of the sample data. [file 1471-2105-14-330-S2.zip › AgalmaExampleTree/65.prot_supermatrix.png]

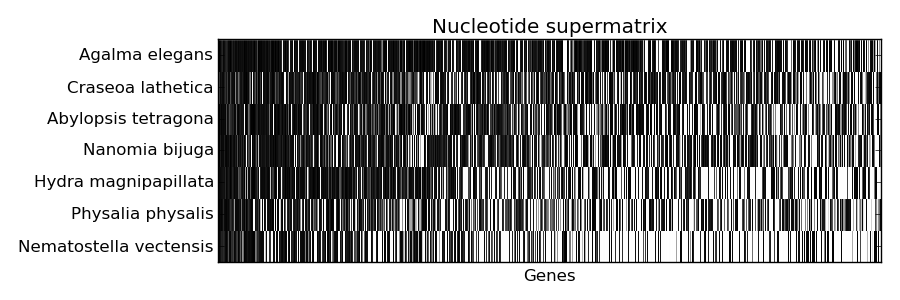

Supplement: Additional file 2 — HTML report for phylogenetic analyses. The HTML report for the phylogenetic analysis of the sample data. [file 1471-2105-14-330-S2.zip › AgalmaExampleTree/65.nuc_supermatrix.png]

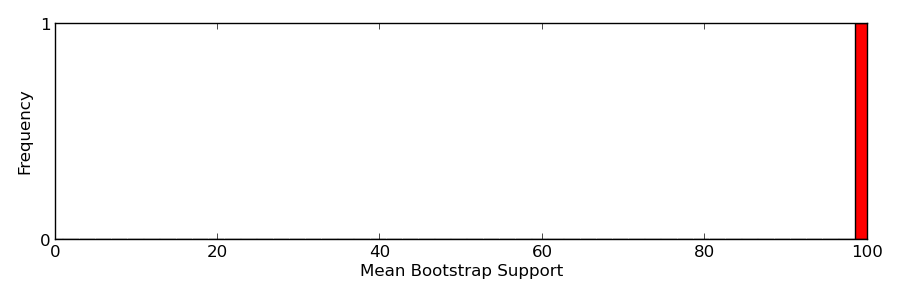

Supplement: Additional file 2 — HTML report for phylogenetic analyses. The HTML report for the phylogenetic analysis of the sample data. [file 1471-2105-14-330-S2.zip › AgalmaExampleTree/69.mean_supports.png]
