# Supplementary material for: Agalma: an automated phylogenomics workflow
Source: BMC Bioinformatics. 2013 Nov 19;14:330. doi: 10.1186/1471-2105-14-330 (PMC3840672; doi:10.1186/1471-2105-14-330)
Supplement: Additional file 2 — HTML report for phylogenetic analyses. The HTML report for the phylogenetic analysis of the sample data. [file 1471-2105-14-330-S2.zip › AgalmaExampleTree/index.html]

AgalmaExampleTree


# *AgalmaExampleTree*

> |  |  |
> | --- | --- |
> | **id** | AgalmaExampleTree |

## Table of Contents

|  |  |  |
| --- | --- | --- |
| **Run 59** *homologize* 2013-06-20T10:10:35.699322 node502 | - homologize | ``` Wall Time (s)      :     28444.75 [sum] User Time (s)      :    443263.13 [sum] System Time (s)    :       256.96 [sum] Memory (KB)        :      236,960 [max] Virtual Memory (KB):      505,276 [max] ``` |
| **Run 60** *multalign* 2013-06-20T18:04:45.268853 node502 | - multalign | ``` Wall Time (s)      :    185305.93 [sum] User Time (s)      :   2820393.03 [sum] System Time (s)    :      2791.37 [sum] Memory (KB)        :      453,212 [max] Virtual Memory (KB):      722,492 [max] ``` |
| **Run 63** *genetree* 2013-06-24T09:08:45.971322 node409 | - genetree | ``` Wall Time (s)      :      5294.13 [sum] User Time (s)      :     71896.19 [sum] System Time (s)    :       250.13 [sum] Memory (KB)        :       43,124 [max] Virtual Memory (KB):      310,228 [max] ``` |
| **Run 64** *treeprune* 2013-06-24T10:37:07.478573 node409 | - treeprune | ``` Wall Time (s)      :       220.64 [sum] User Time (s)      :        56.85 [sum] System Time (s)    :         3.69 [sum] Memory (KB)        :       54,412 [max] Virtual Memory (KB):      320,752 [max] ``` |
| **Run 65** *multalign* 2013-06-24T10:40:51.810980 node409 | - multalign | ``` Wall Time (s)      :     65052.53 [sum] User Time (s)      :    860509.90 [sum] System Time (s)    :      2306.00 [sum] Memory (KB)        :      262,132 [max] Virtual Memory (KB):      531,404 [max] ``` |
| **Run 69** *genetree* 2013-07-01T10:09:48.085955 smp010 | - genetree | ``` Wall Time (s)      :     51481.28 [sum] User Time (s)      :    805559.58 [sum] System Time (s)    :        50.91 [sum] Memory (KB)        :       58,856 [max] Virtual Memory (KB):      325,920 [max] ``` |

## homologize (Run 59)

> Identifies homologous sequences across datasets. Takes assembly or group of
> assemblies and prepares a set of comprehensive comparisons between them. First
> an all by all BLAST is run with a stringent threshold, and the hits which match
> above a given score are used as edges between two transcripts which then form a
> graph. This graph is the basis of a series of comparative scores.

Total sequences: 104174

| Load ID | Catalog ID | Species | NCBI ID | ITIS ID |
| --- | --- | --- | --- | --- |
| 3 | NCBI\_HYDMAG | Hydra magnipapillata | 6085 | 50845 |
| 4 | JGI\_NEMVEC | Nematostella vectensis | 45351 | 52498 |
| 49 | SRX288431 | Physalia physalis | 168775 | None |
| 50 | SRX288276 | Abylopsis tetragona | 316209 | None |
| 54 | SRX288285 | Agalma elegans | 316166 | None |
| 57 | SRX288430 | Nanomia bijuga | 168759 | None |
| 58 | SRX288432 | Craseoa lathetica | 316205 | None |

> Summary of all species processed.

#### Resourse Usage

| Wall Time (s) | User Time (s) | System Time (s) | Memory (KB) | Virtual Memory (KB) |
| --- | --- | --- | --- | --- |
 27983.29 [sum] | 443174.40 [sum] | 255.17 [sum] | 121,208 [max] | 1,371,964 [max] |

 Show/hide details

| Command | Stage | Wall Time (s) | User Time (s) | System Time (s) | Memory (KB) | Virtual Memory (KB) |
| --- | --- | --- | --- | --- | --- | --- |
| makeblastdb | prepare\_blast | 17.45 | 7.08 | 0.12 | 9,596 | 47,052 |
| parallel | run\_blast | 27960.48 | 443160.47 | 223.40 | 121,208 | 196,004 |
| mcl | mcl\_cluster | 5.36 | 6.85 | 31.64 | 103,140 | 1,371,964 |

 Back to TOC

## multalign (Run 60)

> Applies sampling and length filters to each cluster of homologous sequences.
>
> Uses an all-by-all BLAST alignment of sequences within each cluster to trim
> sequence ends not included in any HSP.
>
> Creates multiple sequence alignments for each cluster of homologous sequences
> using Macse, a translation aware aligner that accounts for frameshifts and stop
> codons; see doi:10.1371/journal.pone.0022594

#### Distribution of Frameshifts (by sequences)

#### Distribution of Frameshifts (by clusters)

#### Distribution of Frameshifts (by species)

| species | Frameshifts (% of AA Sequence) |
| --- | --- |
| Abylopsis tetragona | 1.3% |
| Physalia physalis | 1.8% |
| Nanomia bijuga | 1.8% |
| Agalma elegans | 1.7% |
| Nematostella vectensis | 0.2% |
| Hydra magnipapillata | 0.3% |
| Craseoa lathetica | 1.7% |

#### Resourse Usage

| Wall Time (s) | User Time (s) | System Time (s) | Memory (KB) | Virtual Memory (KB) |
| --- | --- | --- | --- | --- |
 178159.39 [sum] | 2815373.02 [sum] | 753.18 [sum] | 19,212 [max] | 758,632 [max] |

 Show/hide details

| Command | Stage | Wall Time (s) | User Time (s) | System Time (s) | Memory (KB) | Virtual Memory (KB) |
| --- | --- | --- | --- | --- | --- | --- |
| makeblastdb | trim\_sequences | 0.04 | 0.01 | 0.01 | 8,144 | 45,008 |
| tblastx | trim\_sequences | 0.20 | 0.16 | 0.24 | 19,212 | 758,632 |
| macse | align\_sequences | 178159.13 | 2815372.86 | 752.92 | 11,324 | 422,948 |
| Gblocks | cleanup\_alignments | 0.02 | 0.00 | 0.01 | 984 | 10,480 |

 Back to TOC

## genetree (Run 63)

> Builds gene trees for each set of homologous sequences, it builds a
> phylogenetic tree using the maximum likelihood optimality criterion as
> implemented in RAxML; see http://www.exelixis-lab.org/ and
> doi:10.1093/bioinformatics/btl446

#### Resourse Usage

| Wall Time (s) | User Time (s) | System Time (s) | Memory (KB) | Virtual Memory (KB) |
| --- | --- | --- | --- | --- |
 6.47 [sum] | 102.64 [sum] | 0.05 [sum] | 9,476 [max] | 1,156,208 [max] |

 Show/hide details

| Command | Stage | Wall Time (s) | User Time (s) | System Time (s) | Memory (KB) | Virtual Memory (KB) |
| --- | --- | --- | --- | --- | --- | --- |
| raxml | genetrees | 6.47 | 102.64 | 0.05 | 9,476 | 1,156,208 |

 Back to TOC

## treeprune (Run 64)

> For each gene tree generated in genetree, it prunes the tree to
> include only one representative sequence per taxon when sequences
> form a monophyletic group (here called 'monophyly masking'). It then
> prunes the monophyly-masked tree into maximally inclusive subtrees with
> no more than one sequence per taxon (here called 'paralogy pruning').

| Cluster Size | Frequency |
| --- | --- |
| 3 | 1791 |
| 4 | 2114 |
| 5 | 1695 |
| 6 | 1275 |
| 7 | 475 |

> Distribution of the number of orthologs in each gene cluster.

 Back to TOC

## multalign (Run 65)

> Applies sampling and length filters to each cluster of homologous sequences.
>
> Uses an all-by-all BLAST alignment of sequences within each cluster to trim
> sequence ends not included in any HSP.
>
> Creates multiple sequence alignments for each cluster of homologous sequences
> using Macse, a translation aware aligner that accounts for frameshifts and stop
> codons; see doi:10.1371/journal.pone.0022594

#### Distribution of Frameshifts (by sequences)

#### Distribution of Frameshifts (by clusters)

#### Distribution of Frameshifts (by species)

| species | Frameshifts (% of AA Sequence) |
| --- | --- |
| Abylopsis tetragona | 1.2% |
| Physalia physalis | 1.7% |
| Nanomia bijuga | 1.8% |
| Agalma elegans | 1.6% |
| Nematostella vectensis | 0.2% |
| Hydra magnipapillata | 0.2% |
| Craseoa lathetica | 1.7% |


> Images of the protein/nucleotide supermatrices, ordered by most
> complete taxa, then most complete gene. Grayscale shading indicates the
> completeness of the gene in that column, where black is complete and
> white is incomplete.

#### Resourse Usage

| Wall Time (s) | User Time (s) | System Time (s) | Memory (KB) | Virtual Memory (KB) |
| --- | --- | --- | --- | --- |
 60009.27 [sum] | 857750.40 [sum] | 436.51 [sum] | 18,012 [max] | 628,640 [max] |

 Show/hide details

| Command | Stage | Wall Time (s) | User Time (s) | System Time (s) | Memory (KB) | Virtual Memory (KB) |
| --- | --- | --- | --- | --- | --- | --- |
| makeblastdb | trim\_sequences | 0.05 | 0.01 | 0.01 | 8,156 | 45,004 |
| tblastx | trim\_sequences | 0.24 | 0.19 | 0.28 | 18,012 | 628,640 |
| macse | align\_sequences | 60008.94 | 857750.20 | 436.22 | 11,320 | 422,948 |
| Gblocks | cleanup\_alignments | 0.03 | 0.00 | 0.00 | 896 | 10,480 |

 Back to TOC

## genetree (Run 69)

> Builds gene trees for each set of homologous sequences, it builds a
> phylogenetic tree using the maximum likelihood optimality criterion as
> implemented in RAxML; see http://www.exelixis-lab.org/ and
> doi:10.1093/bioinformatics/btl446

> Histograms of mean bootstrap supports within each tree.

> Maximum-likelihood tree for the supermatrix.

#### Resourse Usage

| Wall Time (s) | User Time (s) | System Time (s) | Memory (KB) | Virtual Memory (KB) |
| --- | --- | --- | --- | --- |
 50828.31 [sum] | 804907.25 [sum] | 50.47 [sum] | 3,693,016 [max] | 5,639,696 [max] |

 Show/hide details

| Command | Stage | Wall Time (s) | User Time (s) | System Time (s) | Memory (KB) | Virtual Memory (KB) |
| --- | --- | --- | --- | --- | --- | --- |
| raxml | genetrees | 50828.31 | 804907.25 | 50.47 | 3,693,016 | 5,639,696 |

 Back to TOC
