# Supplementary material for: Agalma: an automated phylogenomics workflow
Source: BMC Bioinformatics. 2013 Nov 19;14:330. doi: 10.1186/1471-2105-14-330 (PMC3840672; doi:10.1186/1471-2105-14-330)
Supplement: Additional file 1 — HTML report for assembly of the sample data sets. The HTML report for the assembly of the test data sets from raw reads. The tabular report (index.html) provides an overview across the five assemblies for the ingroup taxa, and includes links (in the Catalog ID column) to detailed reports for the assembly of each species. Fasta files for the annotated transcripts have been removed from the report to reduce file size. [file 1471-2105-14-330-S1.zip › tabular/SRX288430/index.html]

Nanomia bijuga


# *Nanomia bijuga*

> |  |  |
> | --- | --- |
> | **id** | SRX288430 |
> | **ncbi\_id** | 168759 |
> | **library\_id** | SRR871527 |
> | **library\_type** | TRANSCRIPTOMIC |
> | **sequencer** | Illumina HiSeq 2000 |
> | **seq\_center** | Dunnlab |
> | **sample\_prep** | Trizol \| Invitrogen Dynabeads mRNA Purification Kit ; 2 rounds \| Illumina TruSeq RNA Sample Prep Kit |

## Table of Contents

|  |  |  |
| --- | --- | --- |
| **Run 9** *sanitize* 2013-06-11T12:34:28.432527 node422 | - sanitize | ``` Wall Time (s)      :      2073.69 [sum] User Time (s)      :       634.01 [sum] System Time (s)    :      1118.54 [sum] Memory (KB)        :       36,516 [max] Virtual Memory (KB):      297,836 [max] ``` |
| **Run 16** *insert\_size* 2013-06-11T13:09:03.149728 node422 | - insert\_size | ``` Wall Time (s)      :        29.85 [sum] User Time (s)      :        85.75 [sum] System Time (s)    :         6.49 [sum] Memory (KB)        :       43,824 [max] Virtual Memory (KB):      309,004 [max] ``` |
| **Run 17** *remove\_rrna* 2013-06-11T13:09:33.797436 node422 | - remove\_rrna | ``` Wall Time (s)      :      2374.77 [sum] User Time (s)      :     12170.95 [sum] System Time (s)    :       953.60 [sum] Memory (KB)        :       68,072 [max] Virtual Memory (KB):      336,728 [max] ``` |
| **Run 53** *assemble* 2013-06-19T10:21:44.909941 node426 | - assemble | ``` Wall Time (s)      :     36138.40 [sum] User Time (s)      :    165351.03 [sum] System Time (s)    :      7132.82 [sum] Memory (KB)        :       46,628 [max] Virtual Memory (KB):      312,800 [max] ``` |
| **Run 55** *postassemble* 2013-06-19T20:24:12.792157 node426 | - postassemble | ``` Wall Time (s)      :     11162.33 [sum] User Time (s)      :    162573.04 [sum] System Time (s)    :       530.87 [sum] Memory (KB)        :      326,244 [max] Virtual Memory (KB):      597,788 [max] ``` |
| **Run 57** *load* 2013-06-19T23:30:19.141029 node426 |  | ``` Wall Time (s)      :       120.05 [sum] User Time (s)      :         5.09 [sum] System Time (s)    :         1.46 [sum] Memory (KB)        :      133,956 [max] Virtual Memory (KB):      400,432 [max] ``` |

## sanitize (Run 9)

> Filters raw paired-end Illumina data to remove very low quality read pairs,
> read pairs with adapter sequences, and read pairs with highly skewed base
> composition. It then randomizes the order of reads in the files (applying
> the same order of randomization to each file in the pair) to make it simple
> to get random subsets of read pairs in later analyses. Finally, fastqc is
> run to profile the quality of the reads.

#### Illumina Filtering

|  |  |
| --- | --- |
| **Read pairs examined** | 52,533,443 |
| **Read pairs kept** | 39,953,762 |
| **Percent kept** | 76.1% |
| **Illumina quality threshold** | 28 |
| **Adapter fails** | 267,995 |
| **Quality fails** | 14,085,652 |
| **Base composition fails** | 2,087,117 |

#### FastQC reports

|  |  |
| --- | --- |
| 9.fastqc.1   ``` PASS Basic Statistics  PASS Per base sequence quality  PASS Per sequence quality scores  FAIL Per base sequence content  FAIL Per base GC content  PASS Per sequence GC content  WARN Per base N content  PASS Sequence Length Distribution  PASS Sequence Duplication Levels  WARN Overrepresented sequences  WARN Kmer Content ``` | 9.fastqc.2   ``` PASS Basic Statistics  PASS Per base sequence quality  PASS Per sequence quality scores  FAIL Per base sequence content  FAIL Per base GC content  PASS Per sequence GC content  PASS Per base N content  PASS Sequence Length Distribution  WARN Sequence Duplication Levels  WARN Overrepresented sequences  WARN Kmer Content ``` |

> FastQC is a tool from Babraham Bioinformatics that generates detailed
> quality diagnostics of NGS sequence data.

#### Resourse Usage

| Wall Time (s) | User Time (s) | System Time (s) | Memory (KB) | Virtual Memory (KB) |
| --- | --- | --- | --- | --- |
 1677.78 [sum] | 552.88 [sum] | 954.08 [sum] | 21,267,476 [max] | 21,655,492 [max] |

 Show/hide details

| Command | Stage | Wall Time (s) | User Time (s) | System Time (s) | Memory (KB) | Virtual Memory (KB) |
| --- | --- | --- | --- | --- | --- | --- |
| randomize | randomize | 235.64 | 58.29 | 23.71 | 21,267,476 | 21,655,492 |
| fastqc | fastqc | 3.12 | 3.64 | 0.17 | 352,360 | 6,200,312 |
| filter\_illumina | sanitize | 1439.02 | 490.94 | 930.20 | 1,336 | 16,100 |

 Back to TOC

## insert\_size (Run 16)

> Estimates the insert size distribution of paired-end Illumina data by assembling a subset
> of the data and mapping read pairs to it. The insert size does not include the adapters
> added during library preparation.

|  |  |
| --- | --- |
| **Mean insert size (bp)** | 242.82 |
| **Standard deviation (bp)** | 78.05 |

> A histogram of insert sizes.

#### Resourse Usage

| Wall Time (s) | User Time (s) | System Time (s) | Memory (KB) | Virtual Memory (KB) |
| --- | --- | --- | --- | --- |
 28.88 [sum] | 85.33 [sum] | 6.18 [sum] | 525,244 [max] | 1,875,308 [max] |

 Show/hide details

| Command | Stage | Wall Time (s) | User Time (s) | System Time (s) | Memory (KB) | Virtual Memory (KB) |
| --- | --- | --- | --- | --- | --- | --- |
| filter\_illumina | subset | 3.84 | 1.18 | 2.28 | 1,292 | 16,100 |
| velveth | subset\_oases.oases\_assemblies.k31 | 1.23 | 6.07 | 0.30 | 525,244 | 1,809,772 |
| velvetg | subset\_oases.oases\_assemblies.k31 | 4.49 | 10.66 | 0.12 | 173,520 | 1,650,532 |
| oases | subset\_oases.oases\_assemblies.k31 | 0.68 | 0.61 | 0.04 | 53,904 | 71,396 |
| velveth | subset\_oases.oases\_assemblies.k41 | 1.22 | 5.75 | 0.30 | 477,508 | 1,547,628 |
| velvetg | subset\_oases.oases\_assemblies.k41 | 2.82 | 7.17 | 0.09 | 144,616 | 1,649,396 |
| oases | subset\_oases.oases\_assemblies.k41 | 0.55 | 0.47 | 0.05 | 48,640 | 66,120 |
| velveth | subset\_oases.oases\_assemblies.k51 | 1.36 | 6.77 | 0.29 | 464,888 | 1,875,308 |
| velvetg | subset\_oases.oases\_assemblies.k51 | 1.90 | 5.10 | 0.07 | 110,152 | 1,205,172 |
| oases | subset\_oases.oases\_assemblies.k51 | 0.46 | 0.38 | 0.05 | 46,044 | 63,464 |
| velveth | subset\_oases.oases\_assemblies.k61 | 1.40 | 5.88 | 0.26 | 429,396 | 1,875,308 |
| velvetg | subset\_oases.oases\_assemblies.k61 | 1.63 | 4.67 | 0.10 | 110,036 | 1,647,884 |
| oases | subset\_oases.oases\_assemblies.k61 | 0.40 | 0.33 | 0.04 | 45,100 | 62,560 |
| filter\_illumina | stats\_subset | 0.45 | 0.10 | 0.22 | 1,292 | 16,100 |
| bowtie2-build | bowtie | 1.92 | 1.79 | 0.07 | 102,844 | 177,468 |
| bowtie2 | bowtie | 3.20 | 27.19 | 1.80 | 482,868 | 1,301,692 |
| samtools | bowtie\_to\_bam.bam | 0.51 | 0.48 | 0.01 | 4,468 | 21,140 |
| samtools | bowtie\_to\_bam.sort | 0.69 | 0.64 | 0.04 | 140,536 | 161,252 |
| samtools | bowtie\_to\_bam.index | 0.06 | 0.05 | 0.00 | 1,880 | 19,500 |
| insert\_stats | estimate\_insert | 0.06 | 0.04 | 0.01 | 1,292 | 16,072 |

 Back to TOC

## remove\_rrna (Run 17)

> Assembles and identifies ribosomal RNA (rRNA) sequences, removes read pairs
> that map to these rRNA sequences, and provides a variety of diagnostics about
> rRNA. A single exemplar sequence is presented for each type of rRNA that is
> found, but rRNA read pairs are excluded by mapping to a large set of rRNA
> transcripts that are derived from multiple assemblies over a range of data
> subset sizes.

|  |  |
| --- | --- |
| **Read pairs examined** | 39,953,762 |
| **Read pairs kept** | 35,622,853 |
| **Percent kept** | 89.2% |

#### large-mito-rRNA / 1 target(s) / 1,602,065 pairs removed (4.01%)

**Locus\_100000.10\_Transcript\_2/4\_Confidence\_0.600\_Length\_1879:**

|  |  |
| --- | --- |
| **Mean coverage** | 21145.3 |
| **Median coverage** | 14,400 |
| **Min coverage** | 53 |
| **Max coverage** | 59,556 |

  
  

```
  >large-mito-rRNA|Locus_100000.10_Transcript_2/4_Confidence_0.600_Length_1879|Run17|SRX288430
  AATTTAGTTTAAGGTTTTTTCCTTTTAAATTTTAAATCTTTACATTTAAAATCTTTATGTTTTTCTTTTAACATTTTACT
  TAAATAAATTTCACAAGAAACTAGCTATATCCATATTCGTTTAGTTTTTCGCTGCTAATCTTAAATTTTCTAAAAATTTT
  TCAACATTTATTAGTTATTTCTTCAAGTATCTAAGATTAGATCATATGGTTTCGAGTTAGGAAGTTTATAAAGTTACTTT
  TTAAATTTTACTTCATTTTATTTTGTATAATGGATTTACAAAAGGTATATGATTATCAATAATTGTAATTGTTTGTTTAT
  AATTTCCTTCACAGTACTTTTTATATAAATGATTAGTCAATAATTCTTTTATTTTCATTCTTTTTCTATTTCATTCGCCA
  TTACTTTAGAATCGTTCTTTTTTTTATACTAAAATGTTTTAATTCTTTTATAAATTCCTTTTTAGGAAAATCTTATTTTT
  TTTTATCTAATCTTTTATATAAAAAGTACTGTGAAGGAAATTATAAACAAACAATTACAATTATTGATAATCATATACCT
  TTTGTATAATGGATTTACAAAATAAAATGAAGTAAAATTTAAAAAGTAACTTTATAAACTTCCTAACTCGAAACCATATG
  ATCTAATCTTAGATACTTGAAGAAATAACTAATAAATGTTGAAAAATTTTTAGAAAATTTAAGATTAGCAGCGAAAAACT
  AAACGAATATGGATATAGCTAGTTTCTTGTGAAATTTATTTAAGTAAAATGTTAAAAGAAAAACATAAAGATTTTAAATG
  TAAAGATTTAAAATTTAAAAGGAATTTAAAATTTAAAAGGAAAAAACCTTAAACTAAATTTAATATAAAAAAATTAATTA
  TTAATAATAAATTTTTAAAAAATAGAATTTTGGCTTAGAAACAGCCATATTTTAAAGAAAACGTAGTAGTTCACCTTTTT
  AAAAACATAAATAATAAATTTAAATTAATTTAAAAAATTTGTAAGTGGTAACAAGATATTTTACAAAAGAAATTGAAAAA
  TGTAAAAGAAATAATGTAAATATGAATAAAACAAATTTATTTCCAAAGAATTCTATTAAATTTATAAAATAGATAAATAC
  AAAAGAAATTGAAAAATGTAAAAGAAAATGAGAAAAATTGACGATAAAAAAAAAAGACAAATAAAAAATTGTATTATCTA
  AAAAAAGAGGAAATTAAAAAAATAAAAATTCTATATAAGGAACTCGGCAAAATAAAATATCGACTGTTTACCAAAAACAT
  AGCCAAGAATAAAATTCAAGGTGTAACCTGCCCAGTGGTTAATAATTAGAATAATAAACTTAAAATTTAACTAAACGGAT
  GCGGTATCCTAACCGTAATAAAGTAGCATAATCATTCGCCACTTAATTGGTGGATAGTATGAACGGTTAAACGAATATTT
  ACCTGTCTTATATAGAAAATTATGAAATTAAAATAATAGTAAAGATGCTATTTAAAATTGTAAGACGAAAAGACCCTATA
  GAGCTTAACTATTTGCTTTTTTATTAAAATAATCATAGAAAATTATAATAAAAAAAGCTAGGTAGTTTAGTTGGGGCGAC
  TACCTTTTATTAATATCAAAGGTAAACAAAATAATTTATATATTTATTGTATAATCCATCTGTTTAACAATTACAAAAGT
  AGGTAATAATGACCCGTTATTATTAAAAAAGAAAAATAACGATTAATAGATAAAAGCTACCTTAGGGATAACAGGATAAT
  TTTAATTTAGAGACCATATCAAAATTAAAGTTTGTCACCTCTATGTTGAATTAAGATATCCTTATGAAGCAGAATTTATA
  AAGGGTAGGTCTGTTCGACCTTTAAAATCTTACATGATT
```

Top NCBI nt hit: gnl|BL\_ORD\_ID|160295 gi|161338581|emb|AM888344.1| Zygophylax levinseni mitochondrial partial 16S rRNA gene, isolated from Pen Duick Escarpment, Morocco (E-value: 6.56584e-144)

#### large-nuclear-rRNA / 1 target(s) / 2,114,224 pairs removed (5.29%)

**Locus\_50000.227\_Transcript\_1/1\_Confidence\_1.000\_Length\_3570:**

|  |  |
| --- | --- |
| **Mean coverage** | 15992.0 |
| **Median coverage** | 11,861 |
| **Min coverage** | 1 |
| **Max coverage** | 84,556 |

  
  

```
  >large-nuclear-rRNA|Locus_50000.227_Transcript_1/1_Confidence_1.000_Length_3570|Run17|SRX288430
  CTTAGATCAGACAAGACTACCCGCTGAATTTAAGCATATTAATAAGCGGAGGAAAAGAAACTAACAAGGATTCCCTCAGT
  AACGGCGAGTGAAGCGGGACCAGCTCAAACTTAAAATCTCTGTTGCTTGCAACAGCGAATTGTAGTCTCGAGAAGCGTTT
  TCAAGGCGAATGCGCAGTACTTAAGTTGCTTGGAACGGCACATCGTAGAGGGTGACAATCCCGTACGTGGTACTGTGCAT
  CGTTCACGATGCGCTTTCTATGAGTCGGGTTGCTTGGTAATGCAGCCCAAATTTGGAGGTAAACTCCTTCTAAAGCTAAA
  TATTGGCACGAGACCGATAGCGAACAAGTACCGTGAGGGAAAGATGAAAAGCACTTTGAAAAGAAAGTTAATAGTACGTG
  AAACCGTTAGGAGGGAAGCGCATGGAATTAGCAATGCACTGTCGAGATTCAGATGATCGTTGCTTAGTACGGGCGTCGTA
  CGGATCTGAATGGACCGTTGGTGTTCGTCACTGAGTACTGGTTGTCGCATTTCCCGTCAGTGTGCGTCAACAGGTGTTGG
  AATCGGGTGATACGCCTCGCAAGAAGGTGGCTGGTTTCGATCAGTGTTATAGCTTGTGATGTGCTAGCTCGAATTCGACA
  GAGGAGTCGCAGCACATGCCTTTCGGGGCTGGCTTCTGTTTCCTCAGTCTGGCGTGACCATAGCGGACTGCATGCAGTGC
  GTTTGAACTTCGTCCGGCTGTCGGTTGCATGAATGCACACTATGTGCTTAGGTTGTTGGCGGTCATATGGTTTCATGCGA
  CCCGTCTTGGAACACGGACCAAGGAGTCTAACATGTGTGCGAGTCTTAGGGTGATTGAAACCCACAGGCATAATGAAAGT
  AAAGGCTCTTCTGAGCTGAGGTGAGATCTCTTTGATTTCGGTTGGAGAGCGCATCATCGACCGACCTATTCTACTCCTAG
  AAAGGTTTGAGTAAGAGCACATCTGTTGGGACCCGAAAGATGGTGAACTATGCTTGAGTAGGGCGAAGCCAGAGGAAACT
  CTGGTGGAGGCTCGTAGCGATTCTGACGTGCAAATCGATCGTCAAACTTGAGTATAGGGGCGAAAGACTAATCGAACCAT
  CTAGTAGCTGGTTCCCTCCGAAGTTTCCCTTAGGATAGCTGGAACTCGGAACAGTTTTATCAGGTAAAGCGAATGATTAG
  AGGTCTTAGGGTTGAAACAACCTTAACCTATTCTCAAACTTTAAATTGGTAAGAAGCCCGACTTGCTCGACTGAAGTAGG
  GCACAGAATGAGAGTTCTTAGTGGGCCATTTTTGGTAAGCAGAACTGGCGATGCGGGATGAACCGAACGCTGAGTTAAGG
  CGCCTAAATCGACGCTCATCAGACCCCACAAAAGGTGTTGGTTGATCTAGACAGCAGGACGGTGGCCATGGAAGTCGGAA
  TCCGCTAAGGAGTGTGTAACAACACACCTGCCGAATCAACTAGCCCTGAAAATGGATGGCGCTCAAGCGTCGTGCCTATA
  CTCAGCCGTCAGAGTAAATAGCGAAGCTCTGACGAGTAGGAGGGCGTGGGGGTCGTGACGCAGCCTTTGGCGTGAGCCTG
  GGTGAAACGGCCTCTAGTGAAGATCTTGGTGGTAGTAGCAAATATTCAAATGAGAACTTTGAAGACCGAAGTGGAGAAAG
  GTTCCATGTGAACAGCAGTTGGACATGGGTTAGTCGATCCTAAGAGATAGGGAAACTCCGTTTTAAAGTGTCCAATCTTG
  GACCATCGATCGAAAGGGAATCGGGTTAAAATTCCCGAACCAGAACGTGGATATTCTACCCCTTCGGGGTTTAGATGTGC
  GGTAACGCAACTGAACTCGGAGACGTCGGCAGGAGCCCTGGGAAGAGTTCTCTTTTCTTGTTAACGGCCTGACACCATGG
  AATCTGATTGCCAGGAGATATGGTTTGATGGCCGGTAAAGCACCACACTTCATGTGGTGTCCGGTGCGCTCCTGAAGGCC
  CTTGAAAATCCGAGGGAAAGATTGATTTTCACGTCTGTTCGTACTCATAACCGCAGCAGGTCTCCAAGGTGAGCAGCCTC
  TGGTCGATAGAACAATGTAGGTAAGGGAAGTCGGCAAAATAGATCCGTAACTTCGGGAAAAGGATTGGCTCTAAGGGTTG
  GGTCTGTCGGGCTGAGACTTGAAGCGAGTGGATCCAACCTGGACTGGCTTTGGCCTCTCGGGGCTATCGTCGGACTGGGA
  AGGAACTATTCGTGGATTGGCCCAGCTATGCTCGCAAGAGCAGTTCGGCAGGCAATTAACAATCAACTTAGAACTGGTAC
  GGACAAGGGGAATCCGACTGTTTAATTAAAACAAAGCATTGCGATGGTCGGAAACGATGTTGACGCAATGTGATTTCTGC
  CCAGTGCTCTGAATGTCAAAGTGAAGAAATTCAACCAAGCGCGGGTAAACGGCGGGAGTAACTATGACTCTCTTAAGGTA
  GCCAAATGCCTCGTCATCTAATTAGTGACGCGCATGAATGGATTAACGAGATTCCCACTGTCCCTATCTACTATCTAGCG
  AAACCACAGCCAAGGGAACGGGCTTGGCAAAATCAGCGGGGAAAGAAGACCCTGTTGAGCTTGACTCTAGTCTGACTTTG
  TGAAAAGACATAGGAGGTGTAGGATAGGTGGGAGCAGTAATGCAAAAGTGAAATACCACTACTCTTATAGTTTTTTTACT
  TATTCGATTGAGCGGAAGCGAGCTTCACGGCTCATTTTCTAGAATTAAGGCCCCATTGGCGGGTCGATCCGTGTCGAAGA
  CACTGTCAGGTTGGGAGTTTGGCTGGGGCGGCACATCTGTCAAATGATAACGCAGGTGTCCTAAGGTGAGCTCAATGAGA
  ACGGAAATCTCATGTAGAACAAAAGGGTAAAAGCTCACTTGATTTTGATTTTCAGTATGAATACAAACTGTGAAAGCATG
  GCCTATCGATCCTTTAGTCTTTAGGAGTTTTAAGCTAGAGGTGTCAGAAAAGTTACCACAGGGATAACTGGCTTGTGGCA
  GCCAAGCGTTCATAGCGACGTTGCTTTTTGATCCTTCGATGTCGGCTCTTCCTATCATTGTGAAGCAGAATTCACCAAGT
  GTTGGATTGTTCACCCACTAATAGGGAACGTGAGCTGGGTTTAGACCGTCGTGAGACAGGTTAGTTTTACCCTACTGATG
  AAGTGTTGTTGCAATAGTAATTCTGCTCAGTACGAGAGGAACCGCAGATTCAGACAATTGGCATTTGCACTTGCTTGAAA
  AGGCAATGGTGCGAAGCTACCATCTGTTGGATTATGACTGAACGCCTCTAAGTCAGAATCCGTGCTAGAAAGCAATGATA
  ATTACCTCTGGATAATCTTAGGCGAATAAGAATAGAACAGCTTCAGTTGTTCCTGAATCTCAATGCACTGACCTAGAGAA
  AAACTAGTGAGGTGCTGTCATTATCAAATTCTAAAATTTTCAGAGATAAATCCTATGCAGACGACTTAAACAAGAACGTG
  GTATTGTAAAAAGCAGAGTAGCCTCTGTGCTACGATCTTCTGAGATTAAG
```

Top NCBI nt hit: gnl|BL\_ORD\_ID|169594 gi|169643235|emb|AM943655.1| Zygosaccharomyces rouxii 5S rRNA gene, 18S rRNA gene, ITS1, 5.8S rRNA gene, ITS2 and 25S rRNA gene, strain CBS 732 (E-value: 0.0)

#### small-mito-rRNA / no targets

#### small-nuclear-rRNA / 1 target(s) / 614,620 pairs removed (1.54%)

**Locus\_250000.2\_Transcript\_1/1\_Confidence\_1.000\_Length\_1841:**

|  |  |
| --- | --- |
| **Mean coverage** | 12421.6 |
| **Median coverage** | 11,853 |
| **Min coverage** | 1 |
| **Max coverage** | 27,625 |

  
  

```
  >small-nuclear-rRNA|Locus_250000.2_Transcript_1/1_Confidence_1.000_Length_1841|Run17|SRX288430
  CCTGGTTGATCCTGCCAGTAGTCATATGCTTGTCTCAAAGATTAAGCCATGCATGTCTAAGTATAAGCACTTGTACTGTG
  AAACTGCGAATGGCTCATTAAATCAGTTATCGTTTATTTGATTGTACTTTACTACATGGATACCTGTGGTAATTCTAGAG
  CTAATACATGCGAAAAGTCCCGACTTCTGGAAGGGATGTATTTATTAGATTAAAAACCAATGCGAGTCTTCGGGCTCGTT
  TTCTTGGTGATTCATGATAACTTTTCGAATCGCATGGCCTAGAGCCGGCGATATTTCATTCAAATTTCTGCCCTATCAAC
  TGTCGATGGTAAGGTAGTGGCTTACCATGGTTATAACGGGTGACGGAGAATTAGGGTTCGATTCCGGAGAGGGAGCCTGA
  GAAACGGCTACCACATCTAAGGAAGGCAGCAGGCTCGAAAATTACCCAATCCCGACTCGGGGAGGTAGTGACAAGAAATA
  ACGATACGGGGTCTTAATAGGTCTCGCAATTGGAATGAGTACAATTTAAATCCTTTAACGAGGATCAATTGGAGGGCAAG
  TCTGGTGCCAGCAGCCGCGGTAATTCCAGCTCCAATAGCGTATATTAAAGTTGTTGCAGTTAAAAAGCTCGTAGTTGGAT
  TTCGGAGTGGGCCAGTTGGTCCGCCGCAAGGTGTGTTACTGACTGGTTTGCTCTTCTTCGCAAAGACTGCGTGTGCTCTT
  GAGTGAGTGTGCGTAGGATTTACGACGTTTACTTTGAAAAAATTAGAGTGTTCAAAGCAGGCTATTGCTTGAATACATGA
  GCATGGAATAATGGAATAGGACTTTGGTCCCATTTTGTTGGTTTCTAGGGCCGAAGTAATGATTAAGAGGGACAATTGGG
  GGCATCCGTATTTCGTTGTCAGAGGTGAAATTCTTGGATTTACGAAAGACGAACAACTGCGAAAGCACTTGCCAAGAGTG
  TTTTCATTAATCAAGAACGAAAGTTAGAGGATCGAAGACGATCAGATACCGTCCTAGTTCTAACCATAAACGATGTCGAC
  TAGGGATCAGCGGGCGTTATCGTACGACCCCGTTGGCACCTTACGGGAAACCAAAGTTTTTGGATTCCGGGGGAAGTATG
  GTTGCAAAGCTGAAACTTAAAGGAATTGACGGAAGGGCACCACCAGGAGTGGAGCCTGTGGCTCAATTTGACTCAACACG
  GGAAAACTTACCAGGTCCAGACATAGTAAGGATTGACAGGTTGAGAGCCCTTTCTTGATTCTATGGGTGGTGGTGCATGG
  CCGTTCTTAGTTGGTGGAGTGATTTGTCTGGTTAATTCCGTTAACGAACGAGACCTTAACCGGCTAAATAGTCATGCGAT
  TCTCGAATCGTAGCTGACTTCTTAGAGGGACTGTTGGTGTTTAACCAAAGTCAGGAAGGCAATAACAGGTCTGTGATGCC
  CTTAGATGTTCTGGGCCGCACGCGCGCTACACTGTCGGATTCAACGAGTCTTAACCTTAACCGAAAGGTTTGGGTAATCT
  TTTGAAAGTCCGACGTGATGGGGATTGATCATTGCAATTATTGATCATGAACGAGGAATTCCTAGTAAATGCGAGTCATC
  AGCTCGCGTTGATTACGTCCCTGCCCTTTGTACACACCGCCCGTCGCTACTACCGATTGAATGGTTTAGTGAGATCTTCG
  GATTGGCACCGTCGCGGCCTAACGGAAGTGATGGTGCCGAAAAGTTGCTCAAACTTGATCATTTAGAGGAAGTAAAAGTC
  GTAACAAGGTTTCCGTAGGTGAACCTGCGGAAGGATCATTACCGTTTGTCTCTTGACAAAACCACTGTGAATCGTACTGA
  C
```

Top NCBI nt hit: gnl|BL\_ORD\_ID|17796 gi|5931791|emb|AJ133551.1| Virgularia juncea 18S rRNA gene (E-value: 0.0)

#### Resourse Usage

| Wall Time (s) | User Time (s) | System Time (s) | Memory (KB) | Virtual Memory (KB) |
| --- | --- | --- | --- | --- |
 2366.27 [sum] | 12164.09 [sum] | 952.95 [sum] | 2,160,856 [max] | 2,695,044 [max] |

 Show/hide details

| Command | Stage | Wall Time (s) | User Time (s) | System Time (s) | Memory (KB) | Virtual Memory (KB) |
| --- | --- | --- | --- | --- | --- | --- |
| filter\_illumina | subset\_assemblies.500 | 0.03 | 0.00 | 0.01 | 1,292 | 16,100 |
| velveth | subset\_assemblies.500.oases\_assemblies.k61 | 0.04 | 0.14 | 0.07 | 199,220 | 1,834,608 |
| velvetg | subset\_assemblies.500.oases\_assemblies.k61 | 0.04 | 0.14 | 0.02 | 66,940 | 631,008 |
| oases | subset\_assemblies.500.oases\_assemblies.k61 | 0.02 | 0.00 | 0.00 | 1,248 | 19,588 |
| filter\_illumina | subset\_assemblies.1000 | 0.05 | 0.01 | 0.02 | 1,292 | 16,100 |
| velveth | subset\_assemblies.1000.oases\_assemblies.k61 | 0.05 | 0.20 | 0.07 | 203,452 | 1,572,732 |
| velvetg | subset\_assemblies.1000.oases\_assemblies.k61 | 0.06 | 0.31 | 0.02 | 67,276 | 1,679,868 |
| oases | subset\_assemblies.1000.oases\_assemblies.k61 | 0.01 | 0.00 | 0.00 | 1,556 | 28,076 |
| filter\_illumina | subset\_assemblies.2500 | 0.10 | 0.03 | 0.06 | 1,296 | 16,100 |
| velveth | subset\_assemblies.2500.oases\_assemblies.k61 | 0.08 | 0.51 | 0.08 | 208,520 | 1,573,276 |
| velvetg | subset\_assemblies.2500.oases\_assemblies.k61 | 0.08 | 0.26 | 0.03 | 67,892 | 1,680,484 |
| oases | subset\_assemblies.2500.oases\_assemblies.k61 | 0.03 | 0.01 | 0.01 | 2,528 | 36,772 |
| filter\_illumina | subset\_assemblies.5000 | 0.27 | 0.06 | 0.11 | 1,292 | 16,100 |
| velveth | subset\_assemblies.5000.oases\_assemblies.k61 | 0.10 | 0.39 | 0.07 | 216,188 | 2,098,720 |
| velvetg | subset\_assemblies.5000.oases\_assemblies.k61 | 0.12 | 0.48 | 0.03 | 68,924 | 1,681,512 |
| oases | subset\_assemblies.5000.oases\_assemblies.k61 | 0.04 | 0.01 | 0.01 | 3,656 | 37,812 |
| filter\_illumina | subset\_assemblies.10000 | 0.45 | 0.11 | 0.22 | 1,292 | 16,100 |
| velveth | subset\_assemblies.10000.oases\_assemblies.k61 | 0.17 | 0.80 | 0.11 | 230,384 | 2,100,648 |
| velvetg | subset\_assemblies.10000.oases\_assemblies.k61 | 0.18 | 0.64 | 0.03 | 71,060 | 1,290,428 |
| oases | subset\_assemblies.10000.oases\_assemblies.k61 | 0.12 | 0.03 | 0.01 | 5,468 | 39,876 |
| filter\_illumina | subset\_assemblies.25000 | 0.98 | 0.29 | 0.55 | 1,292 | 16,100 |
| velveth | subset\_assemblies.25000.oases\_assemblies.k61 | 0.33 | 1.16 | 0.13 | 268,920 | 2,106,748 |
| velvetg | subset\_assemblies.25000.oases\_assemblies.k61 | 0.42 | 1.49 | 0.05 | 77,480 | 1,493,428 |
| oases | subset\_assemblies.25000.oases\_assemblies.k61 | 0.12 | 0.08 | 0.01 | 11,724 | 42,156 |
| filter\_illumina | subset\_assemblies.50000 | 1.86 | 0.52 | 1.15 | 1,292 | 16,100 |
| velveth | subset\_assemblies.50000.oases\_assemblies.k61 | 0.60 | 2.42 | 0.19 | 317,172 | 1,526,668 |
| velvetg | subset\_assemblies.50000.oases\_assemblies.k61 | 0.81 | 2.74 | 0.06 | 88,244 | 1,241,944 |
| oases | subset\_assemblies.50000.oases\_assemblies.k61 | 0.20 | 0.16 | 0.03 | 22,696 | 44,472 |
| filter\_illumina | subset\_assemblies.100000 | 4.36 | 1.16 | 2.33 | 1,296 | 16,100 |
| velveth | subset\_assemblies.100000.oases\_assemblies.k61 | 1.24 | 5.73 | 0.23 | 429,392 | 2,137,452 |
| velvetg | subset\_assemblies.100000.oases\_assemblies.k61 | 1.69 | 4.70 | 0.08 | 118,188 | 1,645,672 |
| oases | subset\_assemblies.100000.oases\_assemblies.k61 | 0.41 | 0.34 | 0.03 | 45,096 | 62,560 |
| filter\_illumina | subset\_assemblies.250000 | 9.35 | 2.92 | 5.59 | 1,292 | 16,100 |
| velveth | subset\_assemblies.250000.oases\_assemblies.k61 | 2.88 | 12.59 | 0.48 | 698,412 | 2,200,068 |
| velvetg | subset\_assemblies.250000.oases\_assemblies.k61 | 4.54 | 11.34 | 0.15 | 175,304 | 1,328,600 |
| oases | subset\_assemblies.250000.oases\_assemblies.k61 | 1.04 | 0.91 | 0.09 | 112,908 | 130,344 |
| filter\_illumina | subset\_assemblies.500000 | 18.28 | 5.66 | 11.23 | 1,292 | 16,100 |
| velveth | subset\_assemblies.500000.oases\_assemblies.k61 | 5.74 | 27.06 | 0.80 | 1,083,004 | 2,042,280 |
| velvetg | subset\_assemblies.500000.oases\_assemblies.k61 | 9.21 | 26.62 | 0.23 | 264,024 | 1,716,224 |
| oases | subset\_assemblies.500000.oases\_assemblies.k61 | 2.67 | 2.02 | 0.15 | 226,744 | 244,264 |
| filter\_illumina | subset\_assemblies.1000000 | 37.73 | 11.39 | 23.51 | 1,292 | 16,100 |
| velveth | subset\_assemblies.1000000.oases\_assemblies.k61 | 12.93 | 58.87 | 1.53 | 1,402,808 | 2,695,044 |
| velvetg | subset\_assemblies.1000000.oases\_assemblies.k61 | 20.33 | 63.27 | 0.50 | 437,844 | 1,812,092 |
| oases | subset\_assemblies.1000000.oases\_assemblies.k61 | 4.77 | 4.30 | 0.38 | 457,196 | 474,644 |
| makeblastdb | blast\_rrna | 0.03 | 0.01 | 0.01 | 8,172 | 44,988 |
| blastn | blast\_rrna | 10.70 | 10.26 | 0.35 | 215,852 | 2,004,124 |
| blastn | blast\_nt | 1.37 | 1.48 | 0.15 | 137,716 | 2,092,444 |
| bowtie2-build | bowtie | 0.70 | 0.49 | 0.05 | 98,728 | 176,016 |
| bowtie2 | bowtie | 719.27 | 11346.86 | 83.33 | 730,144 | 1,198,924 |
| samtools | bowtie\_to\_bam.bam | 140.30 | 135.66 | 2.37 | 3,392 | 20,100 |
| samtools | bowtie\_to\_bam.sort | 142.07 | 137.87 | 1.09 | 643,364 | 766,652 |
| samtools | bowtie\_to\_bam.index | 6.65 | 6.47 | 0.15 | 940 | 18,744 |
| samtools | bam\_pileup | 91.81 | 86.83 | 1.05 | 53,088 | 71,148 |
| samtools | bam\_extract\_ids.large-nuclear-rRNA | 16.96 | 8.68 | 0.57 | 2,068 | 18,748 |
| samtools | bam\_extract\_ids.small-nuclear-rRNA | 5.84 | 2.60 | 0.19 | 2,072 | 18,748 |
| samtools | bam\_extract\_ids.large-mito-rRNA | 13.63 | 7.49 | 0.45 | 2,072 | 18,748 |
| exclude | exclude\_ids | 1072.44 | 167.50 | 812.72 | 2,160,856 | 2,175,644 |

 Back to TOC

## assemble (Run 53)

> Assembles reads into transcripts, processes the assembly, and generates
> assembly diagnostics. Read pairs are first filtered at a more stringent
> mean quality threshold. Assemblies are then performed over a range of
> data subset sizes, which provides an indication of how sequencing effort
> impacts assembly results.

#### Illumina Filtering

|  |  |
| --- | --- |
| **Read pairs examined** | 31,396,792 |
| **Read pairs kept** | 31,396,792 |
| **Percent kept** | 100.0% |
| **Illumina quality threshold** | 33 |
| **Adapter fails** | 0 |
| **Quality fails** | 0 |
| **Base composition fails** | 0 |

#### Resourse Usage

| Wall Time (s) | User Time (s) | System Time (s) | Memory (KB) | Virtual Memory (KB) |
| --- | --- | --- | --- | --- |
 30298.79 [sum] | 165329.26 [sum] | 7115.79 [sum] | 42,515,052 [max] | 44,132,732 [max] |

 Show/hide details

| Command | Stage | Wall Time (s) | User Time (s) | System Time (s) | Memory (KB) | Virtual Memory (KB) |
| --- | --- | --- | --- | --- | --- | --- |
| filter\_illumina | quality\_filter | 856.25 | 110.05 | 729.73 | 1,340 | 16,100 |
| trinity | trinity.31396792 | 23348.48 | 91573.40 | 2944.03 | 42,515,052 | 44,132,732 |
| parallel | trinity.31396792 | 6094.06 | 73645.81 | 3442.02 | 1,365,444 | 5,239,644 |

 Back to TOC

## postassemble (Run 55)

> Cleans transcripts to remove any rRNA or vector sequences, then selects a
> single exemplar transcript for each gene. Vector sequences could include
> untrimmed adapters or plasmids (we sometimes find sequences in our data for the
> protein expression vectors used to manufacture the sample preparation enzymes).
> Raw reads are mapped back to the exemplars to estimate coverage and assign RPKM
> values. Finally, transcripts are annotated with blastx hits against SwissProt.

#### Assemblies

| Method | Read pairs | Genes | Mean Length (bp) | N50 Length (bp) | Links to assembly files |
| --- | --- | --- | --- | --- | --- |
| trinity | 31,396,792 | 54831 | 813.225328737 | 1011 | [annotated transcripts], [rrna], [vectors] |

##### trinity

|  |  |
| --- | --- |
| **Read pairs** | 31,396,792 |
| **Pairs mapped** | 72.9% |
| **Pairs discordant** | 2.0% |
| **Unpaired reads mapped** | 79.4% |

Number of exemplar transcripts in full assembly with blastx hits: 13,152


#### Resourse Usage

| Wall Time (s) | User Time (s) | System Time (s) | Memory (KB) | Virtual Memory (KB) |
| --- | --- | --- | --- | --- |
 10689.90 [sum] | 162106.72 [sum] | 529.68 [sum] | 390,404 [max] | 1,233,908 [max] |

 Show/hide details

| Command | Stage | Wall Time (s) | User Time (s) | System Time (s) | Memory (KB) | Virtual Memory (KB) |
| --- | --- | --- | --- | --- | --- | --- |
| makeblastdb | clean\_rrna.assembly\_31396792\_trinity | 0.04 | 0.01 | 0.01 | 8,168 | 45,008 |
| blastn | clean\_rrna.assembly\_31396792\_trinity | 12.79 | 93.66 | 16.45 | 168,496 | 230,356 |
| blastn | clean\_univec.assembly\_31396792\_trinity | 13.33 | 90.81 | 23.12 | 168,692 | 231,872 |
| dustmasker | dustmasker.assembly\_31396792\_trinity | 19.08 | 17.96 | 0.97 | 8,260 | 43,572 |
| bowtie2-build | coverage.assembly\_31396792\_trinity | 39.57 | 38.70 | 0.49 | 206,480 | 243,168 |
| bowtie2 | coverage.assembly\_31396792\_trinity | 1174.50 | 18371.78 | 162.02 | 390,404 | 1,233,908 |
| coverage | coverage.assembly\_31396792\_trinity | 169.18 | 69.99 | 10.84 | 8,568 | 24,388 |
| blastx | nr\_annotate.assembly\_31396792\_trinity | 9261.41 | 143423.81 | 315.78 | 214,808 | 359,716 |

 Back to TOC
