# Supplementary material for: Agalma: an automated phylogenomics workflow
Source: BMC Bioinformatics. 2013 Nov 19;14:330. doi: 10.1186/1471-2105-14-330 (PMC3840672; doi:10.1186/1471-2105-14-330)
Supplement: Additional file 1 — HTML report for assembly of the sample data sets. The HTML report for the assembly of the test data sets from raw reads. The tabular report (index.html) provides an overview across the five assemblies for the ingroup taxa, and includes links (in the Catalog ID column) to detailed reports for the assembly of each species. Fasta files for the annotated transcripts have been removed from the report to reduce file size. [file 1471-2105-14-330-S1.zip › tabular/SRX288285/index.html]

Agalma elegans


# *Agalma elegans*

> |  |  |
> | --- | --- |
> | **id** | SRX288285 |
> | **ncbi\_id** | 316166 |
> | **library\_id** | SRR871526 |
> | **library\_type** | TRANSCRIPTOMIC |
> | **sequencer** | Illumina HiSeq 2000 |
> | **seq\_center** | Dunnlab |
> | **sample\_prep** | Trizol \| Illumina TruSeq RNA Sample Prep Kit RNA Purification Beads ; 2 rounds \| Illumina TruSeq RNA Sample Prep Kit |

## Table of Contents

|  |  |  |
| --- | --- | --- |
| **Run 11** *sanitize* 2013-06-11T12:59:00.184170 smp010 | - sanitize | ``` Wall Time (s)      :      3624.41 [sum] User Time (s)      :      1022.73 [sum] System Time (s)    :      2365.35 [sum] Memory (KB)        :       36,212 [max] Virtual Memory (KB):      297,840 [max] ``` |
| **Run 21** *insert\_size* 2013-06-11T13:59:28.788572 smp010 | - insert\_size | ``` Wall Time (s)      :        61.13 [sum] User Time (s)      :       149.20 [sum] System Time (s)    :        10.32 [sum] Memory (KB)        :       43,548 [max] Virtual Memory (KB):      308,984 [max] ``` |
| **Run 22** *remove\_rrna* 2013-06-11T14:00:32.599898 smp010 | - remove\_rrna | ``` Wall Time (s)      :      4224.69 [sum] User Time (s)      :     18411.20 [sum] System Time (s)    :      2252.66 [sum] Memory (KB)        :       67,808 [max] Virtual Memory (KB):      336,740 [max] ``` |
| **Run 45** *assemble* 2013-06-18T21:08:43.133242 node541 | - assemble | ``` Wall Time (s)      :     39046.43 [sum] User Time (s)      :    243254.12 [sum] System Time (s)    :      9178.01 [sum] Memory (KB)        :       46,712 [max] Virtual Memory (KB):      312,984 [max] ``` |
| **Run 51** *postassemble* 2013-06-19T07:59:36.548722 node541 | - postassemble | ``` Wall Time (s)      :     15621.78 [sum] User Time (s)      :    230744.28 [sum] System Time (s)    :       690.15 [sum] Memory (KB)        :      385,972 [max] Virtual Memory (KB):      658,736 [max] ``` |
| **Run 54** *load* 2013-06-19T12:20:02.746636 node541 |  | ``` Wall Time (s)      :       106.93 [sum] User Time (s)      :         6.05 [sum] System Time (s)    :         1.71 [sum] Memory (KB)        :      157,364 [max] Virtual Memory (KB):      423,844 [max] ``` |

## sanitize (Run 11)

> Filters raw paired-end Illumina data to remove very low quality read pairs,
> read pairs with adapter sequences, and read pairs with highly skewed base
> composition. It then randomizes the order of reads in the files (applying
> the same order of randomization to each file in the pair) to make it simple
> to get random subsets of read pairs in later analyses. Finally, fastqc is
> run to profile the quality of the reads.

#### Illumina Filtering

|  |  |
| --- | --- |
| **Read pairs examined** | 53,998,182 |
| **Read pairs kept** | 49,584,637 |
| **Percent kept** | 91.8% |
| **Illumina quality threshold** | 28 |
| **Adapter fails** | 55,487 |
| **Quality fails** | 4,403,926 |
| **Base composition fails** | 1,231,920 |

#### FastQC reports

|  |  |
| --- | --- |
| 11.fastqc.1   ``` PASS Basic Statistics  PASS Per base sequence quality  PASS Per sequence quality scores  FAIL Per base sequence content  FAIL Per base GC content  WARN Per sequence GC content  PASS Per base N content  PASS Sequence Length Distribution  PASS Sequence Duplication Levels  PASS Overrepresented sequences  WARN Kmer Content ``` | 11.fastqc.2   ``` PASS Basic Statistics  PASS Per base sequence quality  PASS Per sequence quality scores  FAIL Per base sequence content  FAIL Per base GC content  WARN Per sequence GC content  PASS Per base N content  PASS Sequence Length Distribution  PASS Sequence Duplication Levels  PASS Overrepresented sequences  WARN Kmer Content ``` |

> FastQC is a tool from Babraham Bioinformatics that generates detailed
> quality diagnostics of NGS sequence data.

#### Resourse Usage

| Wall Time (s) | User Time (s) | System Time (s) | Memory (KB) | Virtual Memory (KB) |
| --- | --- | --- | --- | --- |
 3091.60 [sum] | 872.81 [sum] | 2053.29 [sum] | 21,845,248 [max] | 22,223,968 [max] |

 Show/hide details

| Command | Stage | Wall Time (s) | User Time (s) | System Time (s) | Memory (KB) | Virtual Memory (KB) |
| --- | --- | --- | --- | --- | --- | --- |
| randomize | randomize | 197.83 | 90.19 | 44.46 | 21,845,248 | 22,223,968 |
| fastqc | fastqc | 8.61 | 7.37 | 0.40 | 593,252 | 7,200,740 |
| filter\_illumina | sanitize | 2885.16 | 775.25 | 2008.43 | 1,328 | 16,104 |

 Back to TOC

## insert\_size (Run 21)

> Estimates the insert size distribution of paired-end Illumina data by assembling a subset
> of the data and mapping read pairs to it. The insert size does not include the adapters
> added during library preparation.

|  |  |
| --- | --- |
| **Mean insert size (bp)** | 271.60 |
| **Standard deviation (bp)** | 98.44 |

> A histogram of insert sizes.

#### Resourse Usage

| Wall Time (s) | User Time (s) | System Time (s) | Memory (KB) | Virtual Memory (KB) |
| --- | --- | --- | --- | --- |
 59.25 [sum] | 148.46 [sum] | 9.79 [sum] | 575,072 [max] | 1,400,144 [max] |

 Show/hide details

| Command | Stage | Wall Time (s) | User Time (s) | System Time (s) | Memory (KB) | Virtual Memory (KB) |
| --- | --- | --- | --- | --- | --- | --- |
| filter\_illumina | subset | 6.35 | 1.65 | 4.06 | 1,284 | 16,104 |
| velveth | subset\_oases.oases\_assemblies.k31 | 2.98 | 11.28 | 0.59 | 575,072 | 1,400,144 |
| velvetg | subset\_oases.oases\_assemblies.k31 | 9.21 | 21.64 | 0.25 | 197,900 | 1,103,972 |
| oases | subset\_oases.oases\_assemblies.k31 | 1.47 | 1.17 | 0.09 | 53,940 | 71,384 |
| velveth | subset\_oases.oases\_assemblies.k41 | 2.35 | 9.60 | 0.59 | 542,228 | 1,334,608 |
| velvetg | subset\_oases.oases\_assemblies.k41 | 6.46 | 15.57 | 0.20 | 157,120 | 1,077,596 |
| oases | subset\_oases.oases\_assemblies.k41 | 1.04 | 0.92 | 0.10 | 49,100 | 66,576 |
| velveth | subset\_oases.oases\_assemblies.k51 | 2.36 | 8.61 | 0.50 | 504,700 | 1,334,608 |
| velvetg | subset\_oases.oases\_assemblies.k51 | 4.55 | 11.69 | 0.13 | 130,916 | 1,174,668 |
| oases | subset\_oases.oases\_assemblies.k51 | 0.90 | 0.79 | 0.07 | 46,068 | 63,484 |
| velveth | subset\_oases.oases\_assemblies.k61 | 2.58 | 7.93 | 0.41 | 462,960 | 1,269,072 |
| velvetg | subset\_oases.oases\_assemblies.k61 | 3.68 | 9.35 | 0.15 | 109,928 | 1,155,208 |
| oases | subset\_oases.oases\_assemblies.k61 | 1.32 | 0.72 | 0.07 | 45,124 | 62,536 |
| filter\_illumina | stats\_subset | 0.77 | 0.15 | 0.41 | 1,276 | 16,104 |
| bowtie2-build | bowtie | 5.45 | 5.29 | 0.10 | 103,464 | 178,076 |
| bowtie2 | bowtie | 5.55 | 40.33 | 1.95 | 287,912 | 938,900 |
| samtools | bowtie\_to\_bam.bam | 0.72 | 0.66 | 0.03 | 4,420 | 21,200 |
| samtools | bowtie\_to\_bam.sort | 1.27 | 0.95 | 0.06 | 136,352 | 155,256 |
| samtools | bowtie\_to\_bam.index | 0.14 | 0.07 | 0.01 | 1,832 | 19,500 |
| insert\_stats | estimate\_insert | 0.10 | 0.07 | 0.02 | 1,272 | 16,076 |

 Back to TOC

## remove\_rrna (Run 22)

> Assembles and identifies ribosomal RNA (rRNA) sequences, removes read pairs
> that map to these rRNA sequences, and provides a variety of diagnostics about
> rRNA. A single exemplar sequence is presented for each type of rRNA that is
> found, but rRNA read pairs are excluded by mapping to a large set of rRNA
> transcripts that are derived from multiple assemblies over a range of data
> subset sizes.

|  |  |
| --- | --- |
| **Read pairs examined** | 49,584,637 |
| **Read pairs kept** | 49,389,381 |
| **Percent kept** | 99.6% |

#### large-mito-rRNA / no targets

#### large-nuclear-rRNA / 1 target(s) / 120,652 pairs removed (0.24%)

**Locus\_500000.2613\_Transcript\_1/1\_Confidence\_1.000\_Length\_3582:**

|  |  |
| --- | --- |
| **Mean coverage** | 2033.5 |
| **Median coverage** | 1,770 |
| **Min coverage** | 70 |
| **Max coverage** | 5,863 |

  
  

```
  >large-nuclear-rRNA|Locus_500000.2613_Transcript_1/1_Confidence_1.000_Length_3582|Run22|SRX288285
  CTCAGATCAGACAAGACTACCCGCTGAATTTAAGCATATTAATAAGCGGAGGAAAAGAAACTAACAAGGATTCCCCTAGT
  AACGGCGAGTGAAGCGGGAACAGCTCAAACTTAAAATCTCCGTTGCTTGCAACGGCGAATTGTAGTCTCGAGAAGCGTTT
  TCAAGGCGAATGCGCAGTACTTAAGTTGCTTGGAACGGCACATCGTAGAGGGTGACAATCCCGTACGTGGTACTGTGCAT
  CGTTCACGATGCGCTTTCTATGAGTCGGGTTGCTTGGTAATGCAGCCCAAATTGGGAGGTAAACTCCTTCTAAAGCTAAA
  TATTGGCACGAGACCGATAGCGAACAAGTACCGTGAGGGAAAGATGAAAAGCACTTTGAAAAGAAAGTTAATAGTACGTG
  AAACCGTTAGGAGGGAAGCGCATGGAATTAGCAATGCACTGTCGAGATTCAGACGATCGGTGCTCAGTACGGGCGTCGTA
  CGGATCCGAATGGACCGTTGGCATTCGTCACTTAGTACTGGTTGTCGCATTTCCCGTCAGTGTGCGTCAACAGGTGTTGG
  AATCGGGTGATACGCCTCGCAAGAAGGTGGCTGGTTTCGATCAGTGTTATAGCTTGCGATGTGCTAGCTCGGATCCGACA
  GAGGTGTCGCAGCACATGCCCTCACGGGCTGGCTTCTGTTTCCTCAGTCTTGCGTGACCATAGTGGACTGCGTGCAGTGC
  GCTTGAACTTCGTCGGGCTGTCGGAGGCATGAATGCACACTATGTGCTTAGGTTGTTGGCGGTCATATGGTTTCATGCGA
  CCCGTCTTGTAACACGGACCAAGGAGTCTAACATGTGTGCGAGTCTTAGGGTGATTGAAACCCGCAGGCACAATGAAAGT
  AAAGGCTCTTTTGAGCTGACGTGAGATATCTTTGACTTCGGTTGGAGATCGCATCATGGACCGACCTATTCTACTCCTAG
  AAAGGTTTGAGTAAGAGCACATCTGTTGGGACCCGAAAGATGGTGAACTATGCTTGAGTAGGGCGAAGCCAGAGGAAACT
  CTGGTGGAGGCTCGTAGCGATTCTGACGTGCAAATCGATCGTCAAACTTGAGTATAGGGGCGAAAGACTAATCGAACCAT
  CTAGTAGCTGGTTCCCTCCGAAGTTTCCCTTAGGATAGCTGGAACTCGGAACAGTTTTATCAGGTAAAGCGAATGATTAG
  AGGTCTTAGGGTTGAAACAACCTTAACCTATTCTCAAACTTTAAATTGGTAAGAAGCCCGACTTGCTCGACTGAAGTAGG
  GCACAGAATGAGAGTTCTTAGTGGGCCATTTTTGGTAAGCAGAACTGGCGATGCGGGATGAACCGAACGCTGAGTTAAGG
  CGCCTAAATCGACGCTCATCAGACCCCACAAAAGGTGTTGGTTGATCTAGACAGCAGGACGGTGGCCATGGAAGTCGGAA
  TCCGCTAAGGAGTGTGTAACAACACACCTGCCGAATCAACTAGCCCTGAAAATGGATGGCGCTCAAGCGTCGTGCCTATA
  CTCAGCCGTCAGAGTAAATAGCGAAGCTCTGACGAGTAGGAGGGCGTGGGGGTCGTGACGCAGCCTTTGGCGTGAGCCTG
  GGTGAAACGGCCTCTAGTGAAGATCTTGGTGGTAGTAGCAAATATTCAAATGAGAACTTTGAAGACCGAAGTGGAGAAAG
  GTTCCATGTGAACAGCAGTTGGACATGGGTTAGTCGATCCTAAGAGATAGGGAAACTCCGTTTTAAAGTGCACAATCTTG
  TGCCATCGATCGAAAGGGAATCGGGTTAAAATTCCCGAACCAGAACGTGGATATTCTACCCCTTCGGGGTTTAGATGTGC
  GGTAACGCAACTGAACTCGGAGACGTCGGCAGGAGCCCTGGGAAGAGTTCTCTTTTCTTGTTAACGGCCTGACACCATGG
  AATCTGATTGCCAGGAGATATGGTTTGATGGCCGGTAAAGCACCACACTTCATGTGGTGTCCGGTGCGCTCCTGAAGGCC
  CTTGAAAATCCGAGGGAAAGATTGATTTTCACGTCTGTTCGTACTCATAACCGCAGCAGGTCTCCAAGGTGAGCAGCCTC
  TGGTCGATAGAACAATGTAGGTAAGGGAAGTCGGCAAAATAGATCCGTAACTTCGGGAAAAGGATTGGCTCTAAGGGTTG
  GGTCTGTCGGGCTGAGACTTGAAGCGAGTGGATCCAACCTGGACTGGCTTTGGCCTCTCGGGGCTATGGTCGGACTGGGA
  AGGAACTATTCGTGGATTGGCCCAGCTATGCTCGAAAGAGCAGTTCGGCAGGCAATTAACAATCAACTTAGAACTGGTAC
  GGACAAGGGGAATCCGACTGTTTAATTAAAACAAAGCATTGCGATGGTCGGAAACGATGTTGACGCAATGTGATTTCTGC
  CCAGTGCTCTGAATGTCAAAGTGAAGAAATTCAACCAAGCGCGGGTAAACGGCGGGAGTAACTATGACTCTCTTAAGGTA
  GCCAAATGCCTCGTCATCTAATTAGTGACGCGCATGAATGGATTAACGAGATTCCCACTGTCCCTATCTACTATCTAGCG
  AAACCACAGCCAAGGGAACGGGCTTGGCAAAATCAGCGGGGAAAGAAGACCCTGTTGAGCTTGACTCTAGTCTGACTTTG
  TGAAAAGACATAGGAGGTGTAGGATAGGTGGGAGCATTATGCAAAAGTGAAATACCACTACTCTTATAGTTTTTTTACTT
  ATTCGATTGAGCGGAAGCGAGCTTCACGGCTCATTTTCTAGAATTAAGGCCCCATTGGCGGGTCGATCCGTGTCGAAGAC
  ACTGTCAGGTTGGGAGTTTGGCTGGGGCGGCACATCTGTCAAATGATAACGCAGGTGTCCTAAGGTGAGCTCAATGAGAA
  CGGAAATCTCATGTAGAACAAAAGGGTAAAAGCTCACTTGATTTTGATTTTCAGTATGAATACAAACTGTGAAAGCATGG
  CCTATCGATCCTTTAGTCTTTAGGAGTTTTAAGCTAGAGGTGTCAGAAAAGTTACCACAGGGATAACTGGCTTGTGGCAG
  CCAAGCGTTCATAGCGACGTTGCTTTTTGATCCTTCGATGTCGGCTCTTCCTATCATTGTGAAGCAGAATTCACCAAGTG
  TTGGATTGTTCACCCACTAATAGGGAACGTGAGCTGGGATTAGACCGTCGTGAGACAGGTTAGTTTTACCCTACTGATGA
  AGTGTTGTTGCAATAGTAATTCTGCTCAGTACGAGAGGAACCGCAGATTCAGACAATTGGCATTTGCACTTGCTTGAAAA
  GGCAATGGTGCGAAGCTACCATCTGTTGGATTATGACTGAACGCCTCTAAGTCAGAATCCGTGCTAGAAAGCAATGATAA
  TTACCTCTGGATAATCTTAGGCGAATAAGAATAGAACAGCTTTAGTTGTTCCTGAATCTCAATGCACTGACCTAGAGAAA
  AACTAGTGAGGTGCTGCAACTATCAAAATCTAAAATTTTCAGAGATAAATCCTATGCAGACGACTTAAACAAGAACGTGG
  TATTGTAAAAAGCAGAGTAGCCTCTGTGCTACGATCTTCTGAGATTAAGCCTCTGTTCGTAG
```

Top NCBI nt hit: gnl|BL\_ORD\_ID|119158 gi|111606983|emb|AM181000.1| Sycon capricorn partial 28S rRNA gene, specimen voucher G316187 (Queensland Museum) (E-value: 0.0)

#### small-mito-rRNA / no targets

#### small-nuclear-rRNA / 1 target(s) / 74,604 pairs removed (0.15%)

**Locus\_500000.374\_Transcript\_1/1\_Confidence\_1.000\_Length\_1845:**

|  |  |
| --- | --- |
| **Mean coverage** | 1939.9 |
| **Median coverage** | 2,054 |
| **Min coverage** | 2 |
| **Max coverage** | 3,148 |

  
  

```
  >small-nuclear-rRNA|Locus_500000.374_Transcript_1/1_Confidence_1.000_Length_1845|Run22|SRX288285
  CCTGCCAGTAGTCATATGCTTGTCTCAAAGATTAAGCCATGCATGTCTAAGTATAAGCACTTGTACTGTGAAACTGCGAA
  TGGCTCATTAAATCAGTTATCGTTTATTTGATTGTACTTTACTACATGGATACCTGTGGTAATTCTAGAGCTAATACATG
  CGAAAAATCCCAACTTCTGGAAGGGATGTATTTATTAGATTAAAAACCAATGCGAATTTTCGGATTCGTTTTCTTGGTGA
  TTCATGATAACTTTTCGAATCGCATGGCCTAGAGCCGGCGATATTTCATTCAAATTTCTGCCCTATCAACTGTCGATGGT
  AAGGTAGTGGCTTACCATGGTTATAACGGGTGACGGAGAATTAGGGTTCGATTCCGGAGAGGGAGCCTGAGAAACGGCTA
  CCACATCTAAGGAAGGCAGCAGGCTCGAAAATTACCCAATCCCGACTCGGGGAGGTAGTGACAAGAAATAACGATACGGG
  GTCTTAATAGGTCTCGCAATTGGAATGAGTACAATTTAAATCCTTTAACGAGGATCAATTGGAGGGCAAGTCTGGTGCCA
  GCAGCCGCGGTAATTCCAGCTCCAATAGCGTATATTAAAGTTGTTGCAGTTAAAAAGCTCGTAGTTGGATTTCGGAGTGG
  GCCAGTTGGTCCGCCGCAAGGTGTGTTACTGACTGGTTTGCTCTTCTTCGCAAAGACTGCGTGTGCTCTTGAGTGAGTGT
  GCGTAGGATTTACGACGTTTACTTTGAAAAAATTAGAGTGTTCAAAGCAGGCTATTGCTTGAATACATGAGCATGGAATA
  ATGGAATAGGACTTTGGTCCCATTTTGTTGGTTTCTAGGGCCGAAGTAATGATTAAGAGGGACAATTGGGGGCATCCGTA
  TTTCGTTGTCAGAGGTGAAATTCTTGGATTTACGAAAGACGAACAACTGCGAAAGCACTTGCCAAGAGTGTTTTCATTAA
  TCAAGAACGAAAGTTAGAGGATCGAAGACGATCAGATACCGTCCTAGTTCTAACCATAAACGATGTCGACTAGGGATCAG
  CGGGCGTTATCGTACGACCCCGTTGGCACCTTACGGGAAACCAAAGTTTTTGGATTCCGGGGGAAGTATGGTTGCAAAGC
  TGAAACTTAAAGGAATTGACGGAAGGGCACCACCAGGAGTGGAGCCTGTGGCTCAATTTGACCCAACACGGGAAAACTTA
  CCAGGTCCAGACATAGTAAGGATTGACAGGTTGAGAGCCCTTTCTTGATTCTATGGGTGGTGGTGCATGGCCGTTCTTAG
  TTGGTGGAGTGATTTGTCTGGTTAATTCCGTTAACGAACGAGACCTTAACCGGCTAAATAGTCATGCGATTCTCGAATCG
  TAGCTGACTTCTTAGAGGGACTGTTGGTGTTTAACCAAAGTCAGGAAGGCAATAACAGGTCTGTGATGCCCTTAGATGTT
  CTGGGCCGCACGCGCGCTACACTGTCGGATTCAGCGAGTCTTAACCTTAACCGAAAGGTTTGGGTAATCTTTTGAAAGTC
  CGACGTGATGGGGATTGATCATTGCAATTATTGATCATGAACGAGGAATTCCTAGTAAATGCGAGTCATCAGCTCGCGTT
  GATTACGTCCCTGCCCTTTGTACACACCGCCCGTCGCTACTACCGATTGAATGGTTTAGTGAGATCTTCGGATTGGCACC
  GTCGCGGCCTCACGGAAGTGTTGGTGCCGAAAAGTTGCTCAAACTTGATCATTTAGAGGAAGTAAAAGTCGTAACAAGGT
  TTCCGTAGGTGAACCTGCGGAAGGATCATTACCGTTTGTCTTTTGACAACACCACTGTGAATTGTACCAAGCAGGTGGGG
  TAACG
```

Top NCBI nt hit: gnl|BL\_ORD\_ID|17790 gi|5931716|emb|AJ133559.1| Corynactis sp. 18S rRNA gene (E-value: 0.0)

#### Resourse Usage

| Wall Time (s) | User Time (s) | System Time (s) | Memory (KB) | Virtual Memory (KB) |
| --- | --- | --- | --- | --- |
 4211.22 [sum] | 18400.44 [sum] | 2251.34 [sum] | 1,605,760 [max] | 2,157,076 [max] |

 Show/hide details

| Command | Stage | Wall Time (s) | User Time (s) | System Time (s) | Memory (KB) | Virtual Memory (KB) |
| --- | --- | --- | --- | --- | --- | --- |
| filter\_illumina | subset\_assemblies.500 | 0.05 | 0.01 | 0.02 | 1,288 | 16,104 |
| velveth | subset\_assemblies.500.oases\_assemblies.k61 | 0.13 | 0.59 | 0.10 | 201,276 | 1,228,372 |
| velvetg | subset\_assemblies.500.oases\_assemblies.k61 | 0.05 | 0.34 | 0.01 | 1,360 | 148,096 |
| oases | subset\_assemblies.500.oases\_assemblies.k61 | 0.01 | 0.00 | 0.01 | 1,224 | 20,612 |
| filter\_illumina | subset\_assemblies.1000 | 0.07 | 0.02 | 0.04 | 1,288 | 16,104 |
| velveth | subset\_assemblies.1000.oases\_assemblies.k61 | 0.13 | 0.56 | 0.12 | 203,592 | 1,490,784 |
| velvetg | subset\_assemblies.1000.oases\_assemblies.k61 | 0.26 | 2.01 | 0.03 | 67,104 | 655,856 |
| oases | subset\_assemblies.1000.oases\_assemblies.k61 | 0.02 | 0.01 | 0.00 | 1,544 | 19,872 |
| filter\_illumina | subset\_assemblies.2500 | 0.16 | 0.04 | 0.10 | 1,284 | 16,104 |
| velveth | subset\_assemblies.2500.oases\_assemblies.k61 | 0.18 | 0.59 | 0.11 | 209,180 | 1,235,376 |
| velvetg | subset\_assemblies.2500.oases\_assemblies.k61 | 0.91 | 2.24 | 0.04 | 67,788 | 918,492 |
| oases | subset\_assemblies.2500.oases\_assemblies.k61 | 0.04 | 0.01 | 0.01 | 2,508 | 20,492 |
| filter\_illumina | subset\_assemblies.5000 | 0.46 | 0.08 | 0.20 | 1,288 | 16,104 |
| velveth | subset\_assemblies.5000.oases\_assemblies.k61 | 0.22 | 0.87 | 0.14 | 217,568 | 1,492,480 |
| velvetg | subset\_assemblies.5000.oases\_assemblies.k61 | 0.39 | 2.34 | 0.05 | 68,824 | 1,040,860 |
| oases | subset\_assemblies.5000.oases\_assemblies.k61 | 0.06 | 0.03 | 0.01 | 3,648 | 37,816 |
| filter\_illumina | subset\_assemblies.10000 | 0.90 | 0.14 | 0.43 | 1,288 | 16,104 |
| velveth | subset\_assemblies.10000.oases\_assemblies.k61 | 0.48 | 1.20 | 0.15 | 233,528 | 1,297,804 |
| velvetg | subset\_assemblies.10000.oases\_assemblies.k61 | 0.49 | 1.79 | 0.05 | 70,944 | 1,126,064 |
| oases | subset\_assemblies.10000.oases\_assemblies.k61 | 0.09 | 0.05 | 0.02 | 5,460 | 39,880 |
| filter\_illumina | subset\_assemblies.25000 | 1.83 | 0.40 | 0.98 | 1,284 | 16,104 |
| velveth | subset\_assemblies.25000.oases\_assemblies.k61 | 0.71 | 2.27 | 0.23 | 277,596 | 1,300,804 |
| velvetg | subset\_assemblies.25000.oases\_assemblies.k61 | 1.02 | 3.73 | 0.06 | 77,372 | 1,386,908 |
| oases | subset\_assemblies.25000.oases\_assemblies.k61 | 0.21 | 0.16 | 0.02 | 11,720 | 42,152 |
| filter\_illumina | subset\_assemblies.50000 | 3.03 | 0.78 | 2.05 | 1,288 | 16,104 |
| velveth | subset\_assemblies.50000.oases\_assemblies.k61 | 2.36 | 4.16 | 0.28 | 343,056 | 1,379,180 |
| velvetg | subset\_assemblies.50000.oases\_assemblies.k61 | 1.97 | 5.76 | 0.11 | 88,144 | 1,134,996 |
| oases | subset\_assemblies.50000.oases\_assemblies.k61 | 0.40 | 0.34 | 0.05 | 22,700 | 44,468 |
| filter\_illumina | subset\_assemblies.100000 | 6.82 | 1.59 | 4.04 | 1,284 | 16,104 |
| velveth | subset\_assemblies.100000.oases\_assemblies.k61 | 3.31 | 8.08 | 0.48 | 462,956 | 1,334,604 |
| velvetg | subset\_assemblies.100000.oases\_assemblies.k61 | 4.10 | 10.53 | 0.17 | 109,920 | 1,206,796 |
| oases | subset\_assemblies.100000.oases\_assemblies.k61 | 0.80 | 0.71 | 0.07 | 45,128 | 62,536 |
| filter\_illumina | subset\_assemblies.250000 | 15.32 | 3.77 | 10.32 | 1,288 | 16,104 |
| velveth | subset\_assemblies.250000.oases\_assemblies.k61 | 6.45 | 18.54 | 0.86 | 771,676 | 1,593,832 |
| velvetg | subset\_assemblies.250000.oases\_assemblies.k61 | 10.33 | 24.72 | 0.26 | 170,872 | 1,098,060 |
| oases | subset\_assemblies.250000.oases\_assemblies.k61 | 2.30 | 1.92 | 0.17 | 112,900 | 130,336 |
| filter\_illumina | subset\_assemblies.500000 | 31.31 | 7.37 | 20.78 | 1,288 | 16,104 |
| velveth | subset\_assemblies.500000.oases\_assemblies.k61 | 13.64 | 41.03 | 1.45 | 1,209,036 | 2,157,076 |
| velvetg | subset\_assemblies.500000.oases\_assemblies.k61 | 22.04 | 56.91 | 0.58 | 287,004 | 1,140,104 |
| oases | subset\_assemblies.500000.oases\_assemblies.k61 | 5.48 | 4.29 | 0.40 | 226,804 | 244,332 |
| filter\_illumina | subset\_assemblies.1000000 | 58.90 | 14.74 | 41.10 | 1,288 | 16,104 |
| velveth | subset\_assemblies.1000000.oases\_assemblies.k61 | 21.54 | 80.55 | 2.57 | 1,605,760 | 2,154,208 |
| velvetg | subset\_assemblies.1000000.oases\_assemblies.k61 | 48.15 | 120.11 | 1.31 | 580,308 | 1,419,892 |
| oases | subset\_assemblies.1000000.oases\_assemblies.k61 | 10.14 | 8.95 | 0.95 | 457,240 | 474,696 |
| makeblastdb | blast\_rrna | 0.12 | 0.01 | 0.01 | 8,132 | 44,992 |
| blastn | blast\_rrna | 18.77 | 16.96 | 0.66 | 188,656 | 1,541,696 |
| blastn | blast\_nt | 1.27 | 2.90 | 0.24 | 126,588 | 1,611,360 |
| bowtie2-build | bowtie | 0.54 | 0.30 | 0.07 | 98,488 | 175,904 |
| bowtie2 | bowtie | 1480.40 | 17549.11 | 182.55 | 228,492 | 904,316 |
| samtools | bowtie\_to\_bam.bam | 11.12 | 10.64 | 0.23 | 3,344 | 19,968 |
| samtools | bowtie\_to\_bam.sort | 9.08 | 8.64 | 0.21 | 249,604 | 270,164 |
| samtools | bowtie\_to\_bam.index | 0.73 | 0.55 | 0.03 | 884 | 18,748 |
| samtools | bam\_pileup | 5.45 | 5.15 | 0.05 | 4,576 | 21,316 |
| samtools | bam\_extract\_ids.large-nuclear-rRNA | 0.99 | 0.91 | 0.06 | 2,032 | 18,752 |
| samtools | bam\_extract\_ids.small-nuclear-rRNA | 0.62 | 0.56 | 0.04 | 2,028 | 18,752 |
| exclude | exclude\_ids | 2404.88 | 370.37 | 1976.26 | 99,072 | 113,948 |

 Back to TOC

## assemble (Run 45)

> Assembles reads into transcripts, processes the assembly, and generates
> assembly diagnostics. Read pairs are first filtered at a more stringent
> mean quality threshold. Assemblies are then performed over a range of
> data subset sizes, which provides an indication of how sequencing effort
> impacts assembly results.

#### Illumina Filtering

|  |  |
| --- | --- |
| **Read pairs examined** | 46,234,620 |
| **Read pairs kept** | 46,234,620 |
| **Percent kept** | 100.0% |
| **Illumina quality threshold** | 33 |
| **Adapter fails** | 0 |
| **Quality fails** | 0 |
| **Base composition fails** | 0 |

#### Resourse Usage

| Wall Time (s) | User Time (s) | System Time (s) | Memory (KB) | Virtual Memory (KB) |
| --- | --- | --- | --- | --- |
 36233.47 [sum] | 243226.56 [sum] | 9160.74 [sum] | 46,747,752 [max] | 48,365,428 [max] |

 Show/hide details

| Command | Stage | Wall Time (s) | User Time (s) | System Time (s) | Memory (KB) | Virtual Memory (KB) |
| --- | --- | --- | --- | --- | --- | --- |
| filter\_illumina | quality\_filter | 1208.83 | 170.17 | 1028.33 | 1,340 | 16,100 |
| trinity | trinity.46234620 | 26993.82 | 135564.74 | 3621.55 | 46,747,752 | 48,365,428 |
| parallel | trinity.46234620 | 8030.82 | 107491.66 | 4510.87 | 1,390,836 | 5,237,244 |

 Back to TOC

## postassemble (Run 51)

> Cleans transcripts to remove any rRNA or vector sequences, then selects a
> single exemplar transcript for each gene. Vector sequences could include
> untrimmed adapters or plasmids (we sometimes find sequences in our data for the
> protein expression vectors used to manufacture the sample preparation enzymes).
> Raw reads are mapped back to the exemplars to estimate coverage and assign RPKM
> values. Finally, transcripts are annotated with blastx hits against SwissProt.

#### Assemblies

| Method | Read pairs | Genes | Mean Length (bp) | N50 Length (bp) | Links to assembly files |
| --- | --- | --- | --- | --- | --- |
| trinity | 46,234,620 | 66317 | 922.196872597 | 1283 | [annotated transcripts], [rrna], [vectors] |

##### trinity

|  |  |
| --- | --- |
| **Read pairs** | 46,234,620 |
| **Pairs mapped** | 74.8% |
| **Pairs discordant** | 1.7% |
| **Unpaired reads mapped** | 79.6% |

Number of exemplar transcripts in full assembly with blastx hits: 14,146


#### Resourse Usage

| Wall Time (s) | User Time (s) | System Time (s) | Memory (KB) | Virtual Memory (KB) |
| --- | --- | --- | --- | --- |
 15011.25 [sum] | 230138.96 [sum] | 688.72 [sum] | 413,528 [max] | 1,259,456 [max] |

 Show/hide details

| Command | Stage | Wall Time (s) | User Time (s) | System Time (s) | Memory (KB) | Virtual Memory (KB) |
| --- | --- | --- | --- | --- | --- | --- |
| makeblastdb | clean\_rrna.assembly\_46234620\_trinity | 0.03 | 0.01 | 0.01 | 8,164 | 45,008 |
| blastn | clean\_rrna.assembly\_46234620\_trinity | 13.18 | 108.08 | 19.00 | 169,372 | 227,676 |
| blastn | clean\_univec.assembly\_46234620\_trinity | 14.24 | 112.83 | 24.30 | 169,948 | 229,072 |
| dustmasker | dustmasker.assembly\_46234620\_trinity | 24.29 | 23.03 | 1.12 | 8,280 | 43,704 |
| bowtie2-build | coverage.assembly\_46234620\_trinity | 50.44 | 49.54 | 0.59 | 221,028 | 270,000 |
| bowtie2 | coverage.assembly\_46234620\_trinity | 1714.06 | 26877.80 | 225.38 | 413,528 | 1,259,456 |
| coverage | coverage.assembly\_46234620\_trinity | 249.76 | 103.33 | 15.28 | 10,496 | 26,340 |
| blastx | nr\_annotate.assembly\_46234620\_trinity | 12945.25 | 202864.34 | 403.05 | 231,072 | 377,764 |

 Back to TOC
